# Liquid-Phase Synthesis of 2′-Methyl-RNA on a Homostar Support through Organic-Solvent Nanofiltration

**DOI:** 10.1002/chem.201501001

**Published:** 2015-05-26

**Authors:** Piers R J Gaffney, Jeong F Kim, Irina B Valtcheva, Glynn D Williams, Mike S Anson, Andrew M Buswell, Andrew G Livingston

**Affiliations:** [a] Department of Chemical Engineering, Imperial College South Kensington Campus, London, SW7 2AZ (UK) E-mail: a.livingston@imperial.ac.uk; [b] GSK Medicines Research Centre Gunnels Wood Road, Stevenage, Herts, SG1 2NY (UK)

**Keywords:** homostar, liquid-phase synthesis, nanofiltration, nucleic acids, RNA, solid-phase synthesis

## Abstract

Due to the discovery of RNAi, oligonucleotides (oligos) have re-emerged as a major pharmaceutical target that may soon be required in ton quantities. However, it is questionable whether solid-phase oligo synthesis (SPOS) methods can provide a scalable synthesis. Liquid-phase oligo synthesis (LPOS) is intrinsically scalable and amenable to standard industrial batch synthesis techniques. However, most reported LPOS strategies rely upon at least one precipitation per chain extension cycle to separate the growing oligonucleotide from reaction debris. Precipitation can be difficult to develop and control on an industrial scale and, because many precipitations would be required to prepare a therapeutic oligonucleotide, we contend that this approach is not viable for large-scale industrial preparation. We are developing an LPOS synthetic strategy for 2′-methyl RNA phosphorothioate that is more amenable to standard batch production techniques, using organic solvent nanofiltration (OSN) as the critical scalable separation technology. We report the first LPOS-OSN preparation of a 2′-Me RNA phosphorothioate 9-mer, using commercial phosphoramidite monomers, and monitoring all reactions by HPLC, ^31^P NMR spectroscopy and MS.

## Introduction

Oligonucleotides (oligos) have re-emerged as a major pharmaceutical target due to the unprecedented opportunity for controlling protein expression mediated by short RNA oligomers (ca. 20 nucleotides long) through RNA interference (RNAi) with small interfering RNA (siRNA) or micro-RNA (miRNA), and these have in turn re-invigorated research in the field of anti-sense oligonucleotides (ASO/AS-ON).[1–3] Excitement rose with the recent demonstration of safe and effective delivery of oligos in humans.[4] This imperative has underlined the need for scalable methods of RNA synthesis. Today the overwhelming majority of oligos are prepared using solid-phase oligo synthesis (SPOS), but this is very challenging to scale up.[5] We are developing a liquid-phase oligo synthesis (LPOS) synthetic strategy that will be more amenable to standard batch production techniques than SPOS,[6] using organic-solvent nanofiltration (OSN) as the critical scalable technology for separating the growing oligo from all other reagents.[7] We now report the LPOS-OSN preparation of a 2′-methyl RNA phosphorothioate 9-mer, monitoring all reactions by HPLC, ^31^P NMR spectroscopy and MS.

The defining characteristic of SPOS is the ease of separation of the growing oligo from excess reagents: the solid synthesis support bed/column is simply washed with solvent to remove any molecular species not covalently attached to it. SPOS has been scaled up to 1–2 kg per batch,[5] and the largest trial of the new generation of RNAi therapies required a few kg of oligo.[5] Thus it is expected that 100s of kilograms of oligo might be required annually to treat rare diseases, and possibly tons for major ones. The leading companies in the field have claimed that SPOS can be extended to yet larger scales.[8] However, the specialized equipment is demanding and expensive to use in an industrial setting, and we believe that SPOS, even with major advances, is incapable of approaching the 100 kg scale per batch, because of the challenge of completely and reproducibly washing large beds of synthesis support.[9a] Therefore a very serious gap is expected to open between oligo supply and demand that will restrict this otherwise promising new mode of therapy. Consequently, a new method of oligo production is urgently required.

Early on scalability was identified as the Achilles’ heel of SPOS, and LPOS has long been proposed to overcome this problem.[10] However, the critical question that must be addressed in any LPOS strategy is how to separate the growing oligo from excess reagents and byproducts. So far, amongst the alternative strategies reported for the synthesis of oligos, chromatography has been dismissed as too time-consuming, solvent intensive, and inefficient.[11] Whilst approaches including size-exclusion chromatography[12] and extraction[13] have been proposed to overcome this separation problem, precipitation of polymer-supported oligo has been explored much more widely. Initially DNA oligos supported on poly(styrene) (the same solid-phase support as had recently been used by Merryfield for peptide synthesis)[14] were assembled by means of Khorana’s phosphodiester approach.[10, 15] Subsequent authors explored poly(vinyl alcohol) (PVA)[16, 17] and cellulose[18] as supports, but poly(ethylene glycol) came to dominate this strategy wherein the oligonucleotidyl-PEG was usually precipitated with diethyl ether.[17, 19–21] Recently a discrete, non-polymeric synthesis support was developed in which four oligo chains were grown simultaneously around a pentaerythritol core, and the products were then precipitated from methanol.[22, 23]

Whilst approaches to oligo synthesis based upon precipitation or crystallisation are in theory scalable, it is questionable whether this would be truly practical. Process development of industrial precipitation is often time-consuming and labour-intensive, with the conditions being unique to each compound.[9b] During Bonora’s preparation of 10 mg DNA 20-mer,[20] 79 separate precipitations and crystallisations were required. Furthermore, it is inevitable that material will always be lost to incomplete separation: on the PEG support it was found that losses, starting around 1 % per cycle, became more significant as the increasing solubility of the growing oligo began to overwhelm the polymer-driven phase separation;[20] during the preparation of an RNA 5-mer on the small pentaerythritol support, the coupling cycle yield only averaged 85 % for a 54 % overall yield, which would be unacceptable for commercial production.[23]

The use of membrane-based technologies for the separation of synthetic biopolymers has been little explored, despite their evident potential to realise scalable liquid-phase approaches to valuable targets. In the first such synthesis, Bayer and Mutter bound peptides to mono-methyl PEG-10 000 (mPEG) that was purified by ultrafiltration.[24] This approach was repeated for oligos by the same laboratory, using the now obsolete phosphodiester coupling strategy and either PVA or PEG-10 000 polymeric supports.[17] However, there was no further development of this approach for either biopolymer. Alternation between chain extension in organic solvent and diafiltration in water after each chain extension cycle probably makes this strategy impractical.

**Figure 1:**
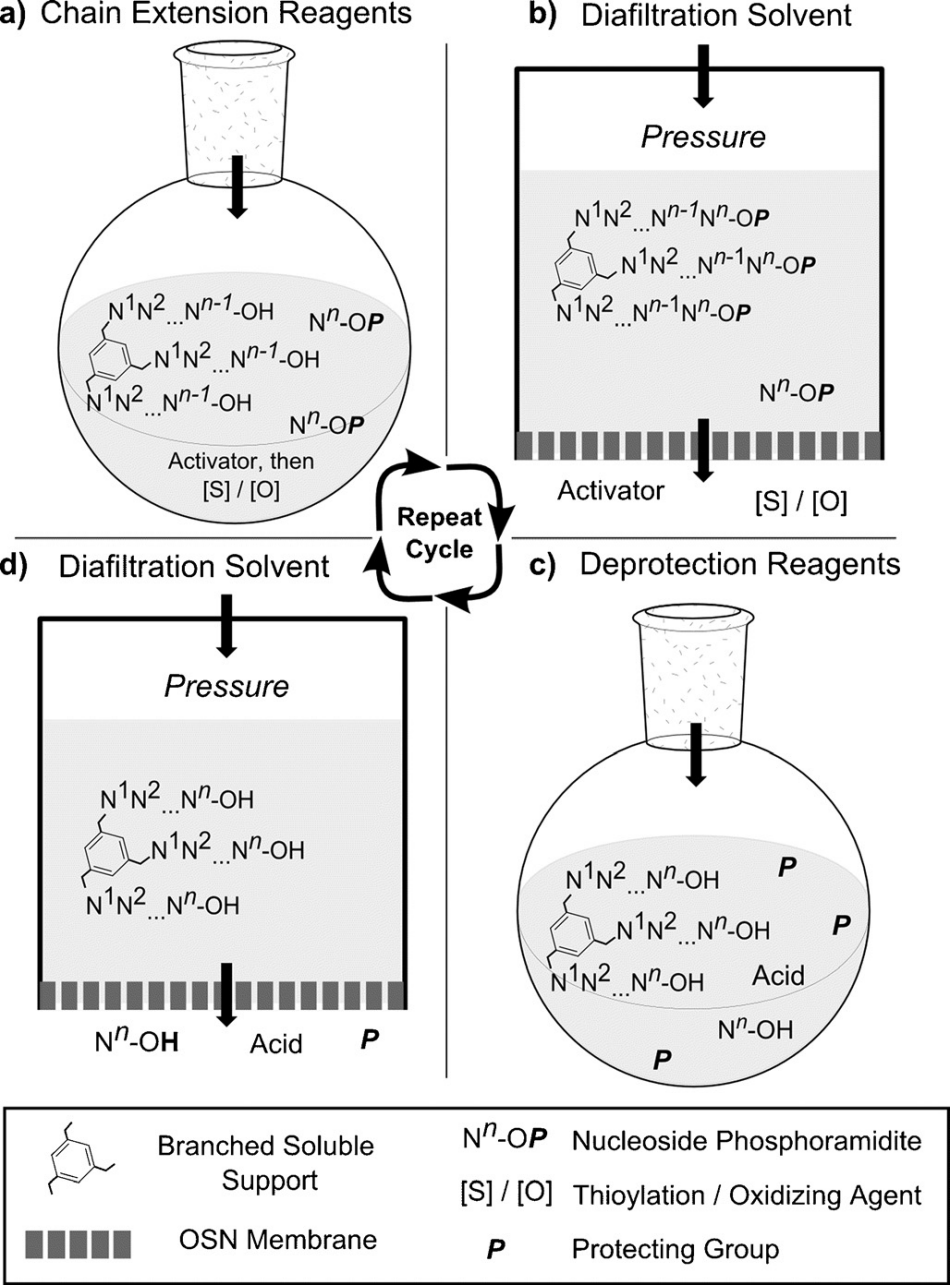
The LPOS-OSN concept: a) Chain extension reaction; b) diafiltration by OSN to remove excess reagents; c) 5′-*O* deprotection; d) diafiltration by OSN to remove excess reagents, then repeat cycle to the desired length.

We postulated that organic-solvent nanofiltration (OSN) could fulfil the critical separation role in an LPOS strategy conducted entirely in organic solution, Figure 1. Furthermore, since organic-solvent stable nanofiltration (as opposed to ultrafiltration) membranes are now available, we proposed that a smaller, discrete synthesis support could be used. During OSN, solutes are separated by size exclusion and geometric selection as they pass through a membrane possessing nanometer-scale permeation pathways. Solutes that cannot pass through the membrane are said to be *rejected* and remain in the upstream *retentate*. This scalable technology is fully compatible with a pharmaceutical industry batch reactor. We further postulated that LPOS-OSN would provide an excellent platform for monitoring the ongoing oligo synthesis, by means of sampling using a simple liquid draw-off. Although in principle it is also possible to monitor chain extension progress with SPOS, we are unaware of any report of such a procedure. This is most likely due to the difficulty of engineering repeated access to beds of solid support in large diameter, pressurized steel columns (columns for preparing 1–2 kg oligo by SPOS have diameters 50–100 cm and pressure ratings of 15–20 bar). Ready access to samples, although providing the opportunity to optimize reactions and to rescue failed steps, is of marginal value on the small synthetic scales regularly produced today. However, in the future, during the preparation of tens of kilograms to tons of oligos, it would be economically unacceptable to risk the complete loss of such large batches of very expensive building blocks without critical quality control.

### Selection of LPOS-OSN materials

**Scheme 1:**
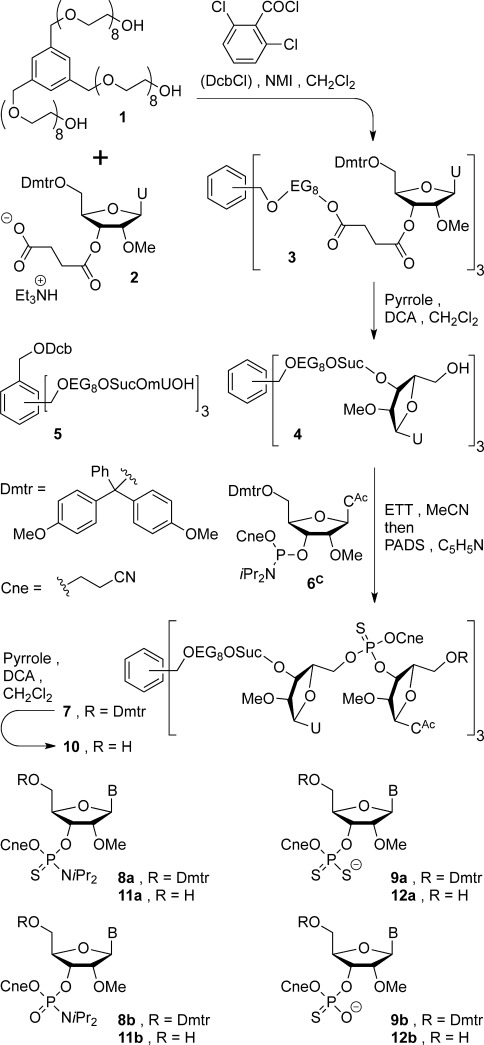
Homostar loading and synthesis of oligonucleotidyl homostars.

Building on our earlier experience with membrane-enhanced peptide synthesis (MEPS) in organic solvent (DMF),[25] we initially explored OSN separation of mPEG-5000-supported dinucleotides.[26] However, even α,ω-bis(dinucleotidyl)-PEG-10 000 had too low a rejection for practical LPOS-OSN. Therefore we instead adopted monodisperse tris(octagol) homostar **1** (a homostar is a star polymer in which all the arms are identical) as a branched LPOS support, see Scheme 1.[27] We hypothesised that this would have three advantages: 1) branching should inhibit threading of the supported oligo into the membrane permeation pathways, and therefore increase membrane rejection of the construct;[28] 2) with three oligos growing around one hub, the molecular weight will rise by three nucleotides per cycle, rapidly increasing the overall size, and hence the rejection of the tris(oligonucleotidyl) support with oligo length; and 3) since the tris(oligonucleotidyl) support is a discrete species, HPLC and mass spectral (MS) analyses of real-time synthetic quality should be feasible.

We next required an OSN membrane compatible with acetonitrile, the solvent in which phosphoramidite couplings are typically conducted. The membrane should also be compatible with feed mixtures containing typical oligo coupling, oxidation/thioylation, capping and 5′-*O*-unblocking reagents. Furthermore, the membrane must be able to permeate nucleotide monomer debris after oligo chain extension; these species are the largest molecular weight debris generated during the synthesis cycle. To meet these challenges we developed a new class of OSN membrane (PBI-17DBX), prepared from poly(benzimidazole) and cross-linked with *para*-dibromoxylene. PBI-17DBX is very resistant to chemical degradation and gave highly reproducible performance in CH_3_CN, whilst being open enough to allow species of similar size to nucleotide monomers to permeate.[29]

Second- and third-generation therapeutic oligos most commonly contain either 2′-deoxy or 2′-modified nucleosides (e.g.; CH_3_O-, CH_3_OCH_2_CH_2_O-, F-), as well as more complex locked/bridged ribose analogues.[30–32] For this reason we elected to focus on nucleic acid analogues for our LPOS-OSN test sequence, instead of native RNA. Adoption of LPOS-OSN by other groups would be encouraged if this technology was compatible with commercial building blocks and common protective group combinations. Therefore we selected readily available 2′-methoxy nucleosides, activated as their 2-cyanoethyl (Cne) *N*,*N*-diisopropylphosphoramidites, carrying 5′-*O*-(4,4′-dimethoxytriphenylmethyl) (Dmtr) temporary protection and with various amides blocking the exocyclic amino groups of the nucleobases.[5] We also selected the widely used, first-generation phosphorothioate modification as a target for this pilot project because the debris from thioylation reagents is likely to be a more severe test of LPOS-OSN purification than common oxidants (e.g. iodine–pyridine–water, or *tert*-butyl hydroperoxide).

All previous LPOS studies, except for that of Lonnberg,[23] have concerned the synthesis of DNA oligos, which are easier to prepare than RNA. Furthermore, with the exception of Bonora’s DNA 20-mer prepared using numerous precipitations and crystallisations,[19] the largest oligo prepared by LPOS to date using iterative synthesis is a DNA 10-mer, through H-phosphonate coupling;[21] a DNA phosphorothioate 15-mer has also been reported, but this was constructed using dimer building blocks.[20] As a challenging target for this new LPOS-OSN technology, we set out to synthesise a 2′-methyl RNA phosphorothioate 9-mer section of the M23D ASO.[33] To assess the performance of the support and phosphoramidite chemistry in this new environment, we planned to undertake global deprotection at both the 5-mer (four chain extensions) and 9-mer (eight chain extensions) stages.

## Results and Discussion

Homostar **1** was first condensed with 4.5 equivalents 5′-Dmtr-2′-methyl-3′-succinyl uridine (**2**, Dmtr-mU-Suc-OH), see Scheme 1. Classical activation with 8 equivalents *N*,*N*′-diisopropyl carbodiimide, in addition to catalytic 4-(dimethylamino)pyridine (DMAP, 0.2 equiv) in THF, was incomplete with excess uridine succinate being consumed as the acyl urea. To maximize the analytical potential of LPOS-OSN it is highly desirable to drive loading of the synthesis support to completion to give a homogeneous product, as well as to avoid waste of expensive excess nucleoside on a large scale. Thus, condensation of 4.5 equivalents uridine succinate (**2**) with homostar **1** was initiated with more reactive 2,6-dichlorobenzoyl chloride (DcbCl) and *N*-methyl imidazole (NMI),[34] after which no PEG-terminal hydroxyls remained. The resultant tris-Dmtr-ether (**3**) was then detritylated with dichloroacetic acid (DCA), using pyrrole as a cation scavenger,[35] to provide fully loaded homostar **4** in 80 % yield over the two steps, ready to commence the chain extension cycle. At this stage a small amount of Dcb-ester (**5**) was separated chromatographically from **4**; although this contaminant would not affect oligo synthesis at all, in this study it was removed to simplify HPLC analysis of chain extension.

The loaded synthesis support **4** (1.24 g) was next chain extended with 5′-Dmtr-2′-methyl *N*-acetylcytidine (Dmtr-mC) phosphoramidite **6^C^** (1.5 equiv per OH) to mUmC homostar **7** under typical conditions, see Scheme 1: ethylthiotetrazole (ETT, 3 equiv per OH) in CH_3_CN, 35 min, then phenylacetyl disulfide (PADS) in pyridine, 30 min, monitoring by HPLC (see Scheme 1 and Supporting Information). For this pilot study unusually long times were used for both coupling and thioylation so that the reactions could be sampled and monitored in real time before moving on to the next process. For this reason, the widely used ETT (p*K*_a_ 4.3, 0.25 m in CH_3_CN) was selected as the activator, firstly because it is a compromise that provides higher activity than classical tetrazole (p*K*_a_ 4.8), but less than 4-nitrophenyl tetrazole (NPT, p*K*_a_ 3.7).[36] Secondly, in larger scale couplings with 2′-methyl phosphoramidites, 0.5 m ETT has proved effective over 5–15 min reaction times,[37] so 0.25 m ETT is commensurate with our longer reactions. Furthermore, although NPT has been reported to give very high coupling yields with 2′-methyl phosphoramidites,[38] we were concerned that the greater acidity of this activator than ETT would exacerbate contamination from double coupling during the long reaction times used here.

During SPOS mass transfer occurs between the bulk solution and the solid support. For fast reactions, such as phosphoramidite coupling, mass transfer is the rate-limiting step.[9a, 24] Thus, if the same chemistry is used in both cases, yields in LPOS are expected to be higher than in SPOS. Consequently, in an LPOS strategy capping should not be as critical as in SPOS, and this step was omitted simplifying the process during pilot study development. Indeed, it is hoped that assay of the chain extension reaction will in future permit identification of otherwise economically catastrophic failed couplings on very large scales, and provide the opportunity to repeat the reaction. However, if capping were implemented before the assay, it would then be impossible to recover a failed coupling.

Once chain extension and thioylation were complete, the crude mixture was then diluted with CH_3_CN, and poured directly into the OSN apparatus (see Supporting Information). The rig was pressurized with nitrogen to force solvent and solutes through the PBI-17DBX membrane, a process termed “diafiltration”. A constant volume of retentate was maintained throughout diafiltration. Thus, the efficiency of OSN can be related to how many retentate system volumes, or “diavolumes”, must be permeated to achieve a given degree of purification of the retentate.

**Figure 2:**
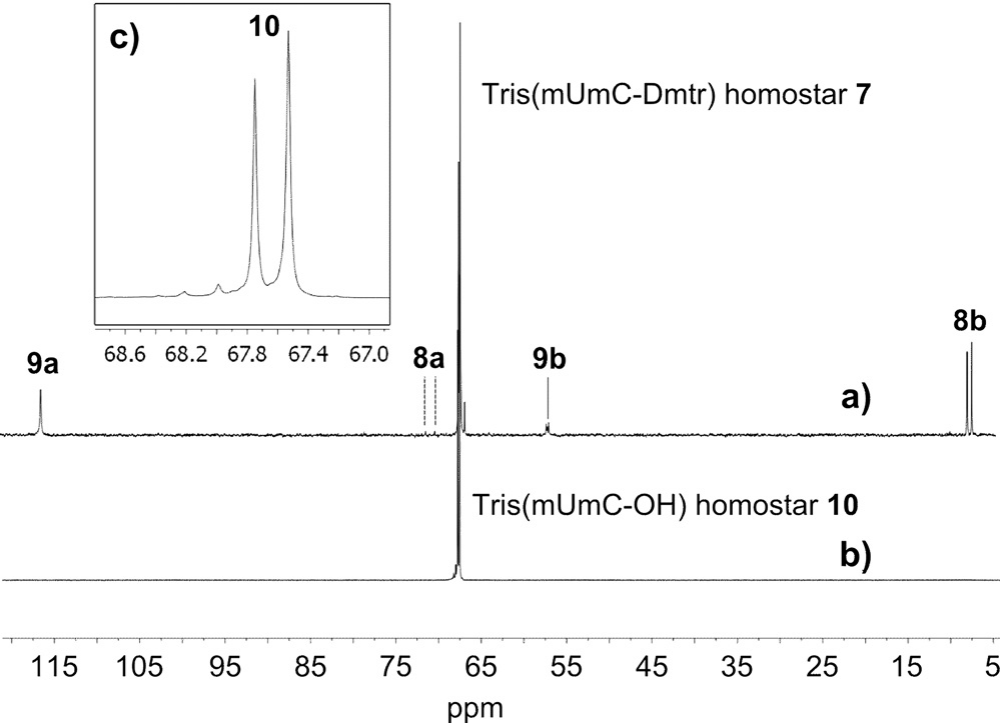
OSN of dinucleotidyl homostar monitored by ^31^P NMR spectroscopy: a) Tris(mUmC-Dmtr) homostar 7 after permeating 12 diavolumes of CH_3_CN—apart from the homostar, amidates 8 a and 8 b, and thioate salts 9 a and 9 b are present; b) tris(mUmC-OH) homostar 10 after permeating 5 diavolumes 1 % DCA-CH_3_CN and 10 diavolumes CH_3_CN; c) expansion of b) exhibiting the two diastereoisomers of the internucleotide linkage, plus a low level of possible *N*-deacetylation of cytosine (see Supporting Information).

After 12 diavolumes, all small molecules had been removed from the retentate. However, along with the desired Dmtr-dinucleotidyl homostar (**7**), most of the building block related species, consisting of a mixture of amidates (**8**) and thioate salts (**9**), were retained (corresponding to N^*n*^-O***P*** in Figure 1). Notably, the proportion of phosphoryl species **8 b** and **9 b** compared to thioyl derivatives **8 a** and **9 a** (P=O vs. P=S), determined by ^31^P NMR spectroscopy of the mixture (see Figure 2 a), increased substantially when the amount of PADS was reduced from 10 to 3 equivalents per 5′-OH. Indeed, thioamidate **8 a** was almost undetectable when the intermediate phosphite was thioylated with 3 equivalents PADS, although amidate **8 b** rose to between 15 and 25 % of the ^31^P NMR signal integral intensity of product **7**.

The crude tris(mUmC-Dmtr) homostar **7** was washed from the OSN rig and re-dissolved in CH_2_Cl_2_. To this were added pyrrole then DCA, and after 30 min the detritylation was complete by HPLC. Unlike in SPOS, in which the detritylation equilibrium is driven to completion by flushing the Dmtr^+^ cation away from 5′-OH oligo bound to the solid support, in solution phase a scavenger (here pyrrole[35]) is necessary to ensure total unblocking. Otherwise even small amounts of mono-tritylated homostar would be carried through to the next cycle where (even after capping) subsequent detritylation would lead to *n*−1 short-mers. It had been anticipated that at this stage the smaller fragments from the excess building block (Dmtr-pyrrole, and 5′-OH amidates **11** and thioates **12**, now corresponding to ***P*** and N^*n*^-OH in Figure 1) would then permeate, but they did not. Suspecting that ion exchange could occur between the protonatable PBI membrane surface and thioate salts **12**, 1 vol % DCA was added to the first five diavolumes. After a total of 15 diavolumes had permeated the product tris(mUmC-OH) homostar **10** was then of a similar purity to that achieved by flash chromatography. However, the detritylated amidates (**11**) were slower to permeate than the thioate salts **12**, and thioamidate **11 a** exhibited substantially greater rejection than amidate **11 b**. Thus by reducing the excess of PADS from 10 to 3 equivalents, when very little or no thioamidate **11 a** formed, the purity of dinucleotidyl homostar **10** was maximised (Figure 2 b). Dmtr-pyrrole was the only contaminant significantly rejected by PBI 17DBX (see HPLC in Supporting Information). Thus, although Dmtr-pyrrole probably does not interfere with subsequent couplings, this was removed by precipitation of tris(mUmC-OH) homostar **10** in diethyl ether so that an accurate mass recovery could be determined; apart from Dmtr-pyrrole no other species could be detected in the supernatant by ^1^H or ^31^P NMR spectroscopy. After the first chain extension cycle a moderate 75 % yield of tris(mUmC-OH) homostar **10** was isolated. The only detectable impurity was a low level of cytosine *N*-deacetylation, identified by LC-MS (see Supporting Information) and believed to be the minor peaks in Figure 2 c.

**Scheme 2:**
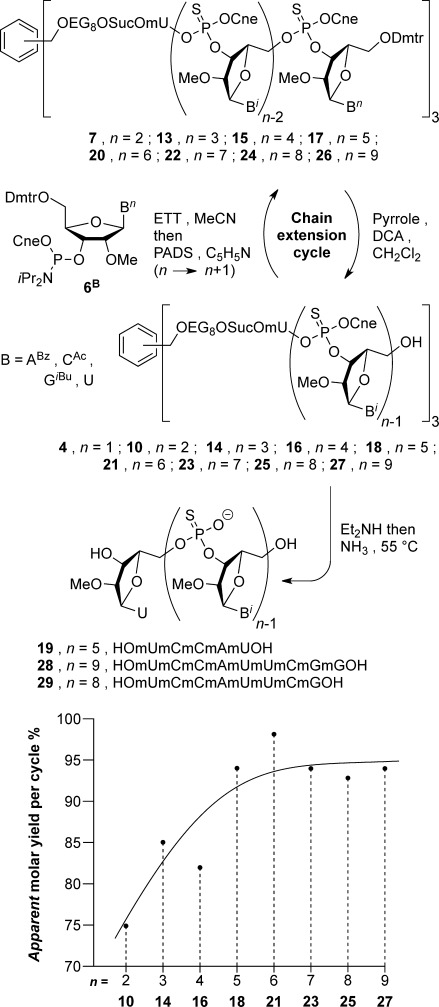
Chain extension cycle. Inset: Change in apparent yield of isolated 5′-OH tris(oligonucleotidyl) homostar with oligo length.

Despite the need to remove Dmtr-pyrrole by precipitation, the above cycle was repeated on homostar **10**, see Scheme 2; from this point on, all chain extension cycles start with 1.2–1.4 g tris(5′-HO-oligo) homostar. Thus, after chain extension, Dmtr-3-mer homostar **13** was partially purified by OSN (12 diavolumes) then detritylated, after which all the nucleotidyl debris was separated by OSN, and precipitation was again used to remove residual Dmtr-pyrrole. This time during the second diafiltration, the first five diavolumes contained only 0.1 % DCA to minimize N-deacetylation. The 85 % yield of tris(mUmCmC-OH) homostar **14** was significantly higher than that of dinucleotidyl homostar **10** at the same stage (see Scheme 2, inset graph), indicating that as expected the homostar rejection had risen with oligo length. It should be noted that chain extension cycle yields are calculated assuming 100 % purity of the product homostar. However, as low levels of side-reactions accumulate on the growing oligo, the purity cannot be 100 %, so the molecular weight cannot be precisely defined, and the yields are more correctly referred to as apparent yields.

Both the tritylated (**13**) and detritylated (**14**) tris(trinucleotidyl) homostars were less soluble in CH_3_CN than the shorter species **4**, **7** and **10**—a trend that continued with increasing length. Noting that all the oligonucleotidyl homostars (**14**–**18** and **20**–**27**) were highly soluble in DMF, all subsequent phosphoramidite couplings were conducted in CH_3_CN–DMF (ca. 9:1); this solubility of a branched 2′-Me-RNA 24-mer oligonucleotidyl homostar (**18**) may be favorably contrasted with the previously reported poor solubility of 5′-OH DNA 8-mers in CH_3_CN.[39] The solvent was also changed during OSN from neat CH_3_CN, in which tris(tetranucleotidyl) homostar **16** is almost insoluble, to CH_3_OH–CH_3_CN (1:4 or 1:3 v/v) in which all the oligonucleotidyl homostars are soluble up to at least tris(9-mer) homostar **27** (0.4 wt % **27** during final diafiltration; the saturation conc. was not determined). Finally, the DCA in the second diafiltration was replaced by 1 % pyridinium dichloroacetate (Py⋅DCA) which promoted permeation of thioate salts **12** just as effectively as un-buffered DCA. This protocol was used on 4-mer **16** and for all later chain extension cycles, following each reaction by HPLC, and assaying the products by ^31^P NMR spectroscopy and MALDI MS, both before and after detritylation (see Supporting Information).

Two further rounds of chain extension were conducted, with the apparent yield continuing to rise (82 % **16**, 94 % **18**, see inset graph, Scheme 2). Although HPLC usefully exhibited retention times lengthening in relation to the number of 5′-Dmtr ethers per homostar, both during chain extension and detritylation, by 5-mers **17** and **18** the peaks were too broad to be of further analytical use (see Supporting Information), presumably due to the exponentially growing number of diastereoisomers at the P-centers of the oligo backbone. However, ^31^P NMR spectroscopy continued to demonstrate acceptably low levels of amidate contamination after each cycle. Furthermore, MS confirmed that full-length tris(mUmCmCmAmU-OH) homostar **18** was the principal product. By contrast, MS of an oligo conjugated to a polydisperse support would be spread over too many polymeric homologues to provide sufficiently intense peaks for analysis.

**Figure 3:**
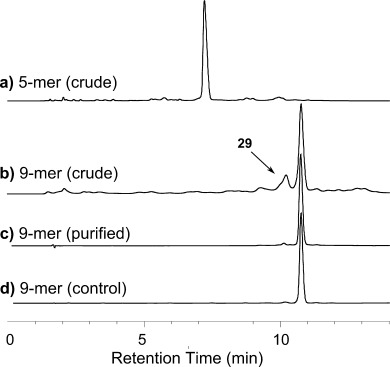
HPLC of deprotected oligos: a) crude 5-mer 19, 75 % purity; b) crude 9-mer 28, 49 %, containing 8-mer 29, 18 %; c) purified 9-mer 28, 94 %, from LPOS-OSN; d) 9-mer 28, 95 %, from SPOS.

Pentanucleotidyl homostar **18** was deprotected first with diethylamine, then overnight in aqueous ammonia at 55 °C. The following day, trituration with CH_3_CN removed protective group debris to give crude 2′-methyl RNA phosphorothioate 5-mer **19**. HPLC assay of this material (Figure 3 a) exhibited a moderate purity of 74 %, with both short-mer and long-mer contaminants. Although these short-mers could be explained by lack of capping, we believe that they actually derive from chain extension of residual amidate building block **11** after OSN. Examination of the mass spectra of the 5′-OH tris(oligonucleotidyl) homostars **10**, **14**, **16** and **18** (from 2-mer to 5-mer) exhibit no detectable ions corresponding to incomplete chain extension. Since our synthesis support possesses three arms, if 1 % incomplete chain extension had occurred, this would afford approximately 3 % homostar having one arm bearing the *n*−1 short-mer. Thus, assuming that MALDI ionization of full-length oligohomostars and their singly truncated oligohomostar contaminants are similar, mass spectral analysis of homostars supported oligonucleotides should usefully amplify sequence errors to detectable amounts. The long-mers probably arise from two sources: 1) relatively long coupling times compared to SPOS (35 min vs. 6–12 min) were used here to allow time for HPLC confirmation of complete coupling. This favors double coupling due to ETT induced detritylation.[40] 2) N-Deacetylation of cytosine residues (as observed at the dimer stage) could provide sites for branching, although we suspect that the switching from DCA to Py⋅DCA in the second diafiltration of each cycle largely suppressed this.

Chain extension was continued from tris(pentanucleotidyl) homostar **18** with the same protocol for another four cycles, and ^31^P NMR spectroscopy and MS now served as the principal methods of product characterization, to obtain 1.36 g of the desired tris(nonanucleotidyl) homostar **27**. Apparent yields of detritylated 6- to 9-mer homostars **21**, **23**, **25** and **27** now plateaued around an average of 95 % (see inset graph, Scheme 2). Finally, at the 9-mer stage MS indicated incomplete coupling with approximately 90 % conversion per chain (**25→26**). This material was detritylated and deprotected as before to characterize the crude 9-mer by HPLC (Figure 3 b); 266 mg 5′-OH 9-mer homostar **27** gave 180 mg crude 9-mer **28**. As implied by the homostar MS, HPLC indicated 49 % of the desired 9-mer **28**, plus 17 % of the expected 8-mer impurity **29** (confirmed by LC-MS, see Supporting Information). The 9-mer was then fractionated for confirmatory analysis through two ion-exchange columns to 94 % purity (Figure 3 c), and desalted providing a 16 % (29 mg) yield of pure 9-mer **28** from 5′-OH homostar **27**. This material displayed identical HPLC (Figure 3 d) and MS to 9-mer **28** prepared by SPOS.

## Conclusion

In this report we have demonstrated for the first time a new liquid-phase synthesis and separation paradigm for oligonucleotides: liquid-phase oligonucleotide synthesis/organic-solvent nanofiltration, LPOS-OSN. This was used to prepare 2′-methyl RNA phosphorothioate 9-mer **28**. This promising technology has yet to equal the speed and purity of SPOS, requiring around two days per chain extension cycle with the limited area available from current laboratory-scale flat membrane cells. However, the fact that we were able to perform eight chain extension and detritylation cycles, with intermediate purifications, all in the liquid phase demonstrates that it has high potential. Compared to competing precipitation strategies, LPOS-OSN is more amenable to industrial exploitation because liquid-phase handling is intrinsically scalable. LPOS-OSN also has the major advantage over SPOS that it is straightforward to sample and monitor every step of the process. Apart from ^31^P NMR spectroscopy and HPLC, the choice of a monodisperse support also allowed characterization of the growing oligonucleotidyl homostars by MS.

From the above experience, several modifications can be suggested to improve future protocols: Shortening the coupling time, and analysing only after thioylation, will reduce long-mer formation.[40] Minimising 5′-unblocking time, and therefore acid exposure, will minimise cytosine deacetylation, and again possible long-mer formation. Biasing building block debris away from amidates (**8**/**11**) into thioates (**9**/**12**) that are more easily removed by diafiltration would suppress short-mer formation. The overall yield of fully protected 5′-OH tris(oligonucleotidyl) homostar **27** from loaded uridine homostar **4** is only about 39 %, mainly due to poor recovery from the early cycles of OSN; the first three couplings (**4→**4-mer **14**) give a cumulative yield of only 52 %, but the next five couplings (4-mer **18→**9-mer **27**) have a combined yield of about 76 %. This will be much improved using our recently developed 2-stage diafiltration; we would expect early stage recovery to be >95 % and from 5-mer onwards >99 %.[41] As with SPOS, LPOS-OSN consumes a lot of solvent. We have recently demonstrated that an additional stage of diafiltration with a low-molecular-weight cut-off membrane can be used to recycle the permeate solvent, greatly reducing the potential cost on an industrial scale.[42] As the scale of LPOS-OSN increases, an alternative analysis to direct HPLC of the retentate will be required; we believe that rapid ammonia-methylamine (AMA) global deprotection, followed by HPLC of the crude unblocked oligo will provide a suitable method to assay for complete chain extension.[43] Finally, identifying a membrane that permeates Dmtr derivatives, or using a smaller 5′-protecting group, such as the methoxyisopropylidene acetal,[23] would make the process even more efficient.

## Experimental Section

### General experimental details

^1^H and ^13^C NMR spectra were recorded on Brüker AV-400 or Brüker AV-500 spectrometers. Chemical shifts in ppm are referenced with respect to residual solvent signals: *δ*_H_ (CHCl_3_) 7.25 ppm, *δ*_H_ (CHD_2_OD) 3.31 ppm, *δ*_H_ (CD_3_COCHD_2_) 2.05 ppm; *δ*_C_ (CDCl_3_) 77.50 ppm, *δ*_C_ (CD_3_OD) 49.15 ppm, *δ*_C_ (CD_3_COCHD_2_) 29.92 ppm. The splitting patterns for ^1^H NMR spectra are denoted as follows; s (singlet), d (doublet), t (triplet), q (quartet), quin (quintet), m (multiplet), br (broad) and combinations thereof. Coupling constants (*J*) are in Hertz (Hz). ^13^C NMR assignments (***C***, ***C***H, ***C***H_2_ and ***C***H_3_) and ^1^H NMR assignments were established with the aid of DEPT-135, HSQC and COSY experiments. Molecular fragments not abbreviated in main text are denoted as follows: U, uracil; C, cytosine; Ri, ribose; Suc, succinate; Hub, C_6_H_3_(CH_2_OR)_3_. CDCl_3_ was purchased from VWR, and CD_3_OD and CD_3_COCD_3_ from Merck. NMR spectrscopy of small Dmtr derivatives was conducted in the presence of either a small amount of Et_3_N or of pyridine. Mass spectra were recorded on Micromass MALDI micro MX, or Micromass LCT Premier (ESI) mass spectrometers. Phosphoramidites and 2′-*O*-methyl uridine were purchased from Fisher Scientific Ltd., UK or ChemGenes Corp., USA. Other reagents were purchased from Sigma-Aldrich Ltd. and used as supplied, except where specified. Reactions were carried out under anhydrous conditions under a nitrogen atmosphere. Dichloromethane, acetonitrile, THF and DMF were dried and stored over baked 4 Å molecular sieves. Triethylamine, diethyl ether, methanol, isopropanol and *N*-methyl imidazole were used as supplied. Flash chromatography was conducted in a 9 cm diameter, porosity 3 glass sinter funnel: Geduran® (Si 60) from Merck was used for normal phase columns, and Merck silanised silica for reverse phase columns. Thin-layer chromatography was carried out using Merck silica gel 60 F_254_ aluminium-backed plates; compounds were visualised using UV light or KMnO_4_ stain. Solid phase oligonucleotide synthesis (SPOS) was carried out on a GE AKTA Oligopilot 10, using preloaded 2′OMe U Primer Support 200 and manufacturer’s standard protocols on a 30 μmol scale. Cleavage from solid support and deprotection of nucleobases was carried out in 0.88 aqueous ammonia at 55 °C for 16 h.

### Synthetic procedures

**Tris-1,3,5-{ω-[2′-*O*-methyl-5′-O-(4′′,4′′′-dimethoxytriphenylmethyl)uridine-3′-*O*-succinyloxy]octa(ethylene glycol)-α-oxymethyl}benzene (3)**: Compound **1** (1.125 g, 1.00 mmol) was co-evaporated from CH_3_CN (3×15 mL), re-dissolved in CH_2_Cl_2_ (7 mL) and a dropping funnel was fitted to the flask. 2′-*O*-Methyl-3′-*O*-succinyl-5′-*O*-(4′′,4′′′-dimethoxytriphenylmethyl)uridine, triethylammonium salt (2, 3.047 g, 4.00 mmol) was co-evaporated from THF (3×50 mL), then re-dissolved in CH_2_Cl_2_ (14 mL) and *N*-methylimidazole (0.63 mL, 7.9 mmol) then 2,6-dichlorobenzoyl chloride (DcbCl, 0.54 mL, 3.77 mmol) were added. After stirring for 20 min, the activated succinate solution was transferred to the dropping funnel, rinsing out the flask with further CH_2_Cl_2_ (5 mL), and this solution slowly added to homostar **1** over 45 min. After stirring over night the reaction was diluted with CH_2_Cl_2_ (250 mL) and extracted with sat. NaHCO_3_ (200 mL). The aqueous layer was back-extracted with small portions of CH_2_Cl_2_ (50 mL×3), the combined organic layers dried over Na_2_SO_4_, and the solvent stripped off in vacuo. The residual foam may be purified chromatographically by fractionation through a column of silanised silica, eluting with a gradient of CH_3_CN–water (3:7 to 8:2 v/v containing 0.5 % sat. NaHCO_3_), but was normally used without purification. *R*_f_ (EtOH–CHCl_3_ 1:9 + trace Et_3_N) 0.56; ^1^H NMR (400 MHz, CDCl_3_): *δ*=7.88 (d, *J*=8.2 Hz, 3 H; U C***H***), 7.38–7.36 (m, 6 H; Dmtr C***H***), 7.32 (t, *J*=7.5 Hz, 6 H; Dmtr C***H***), 7.29–7.24 (m, 18 H; Hub C***H*** + Dmtr C***H***), 6.86 (d, *J*=8.9 Hz, 12 H; Dmtr C***H***), 6.04 (d, *J*=3.7 Hz, 3 H; 1′-C***H***), 5.33 (d, *J*=8.2 Hz, 3 H; U C***H***), 5.29 (t, *J*=5.6 Hz, 3 H; 3′-C***H***), 4.55 (s, 6 H; Hub-C***H***_***2***_O), 4.26–4.24 (m, 9 H; 4′-C***H*** + Suc-OC***H***_***2***_), 4.10 (dd, *J*=5.1, 3.7 Hz, 3 H; 2′-C***H***), 3.81 (s, 18 H; OC***H***_***3***_), 3.71–3.58 (m, 93 H; C***H***_***2***_O + 5′-CH***H***), 3.48–3.44 (m, 12 H; 2′-OC***H***_***3***_ + 5′-CH***H***), 2.73–2.66 ppm (m, 12 H; Suc C***H***_***2***_); ^13^C NMR (101 MHz, CDCl_3_): *δ*=171.98 (3 C; Suc *C*=O), 171.53 (3 C; Suc ***C***=O), 163.29 (3 C; U ***C***), 158.73 (6 C; Dmtr ***C***), 150.30 (3 C; U ***C***), 144.13 (3 C; Dmtr ***C***), 139.78 (3 C; U 6-***C***H), 138.57 (3 C; Hub ***C***), 134.98 (3 C; Dmtr ***C***), 134.86 (3 C; Dmtr ***C***), 130.17 (6 C; Dmtr ***C***H), 130.09 (6 C; Dmtr ***C***H), 128.08 (12 C; Dmtr ***C***H), 127.24 (3 C; Dmtr ***C***H), 126.32 (3 C; Hub ***C***H), 113.33 (12 C; Dmtr ***C***H), 102.51 (3 C; U 5-***C***H), 87.39 (3 C; Dmtr ***C***), 87.13 (3 C; 1′-***C***H), 82.18 (3 C; Ri ***C***H), 81.03 (3 C; Ri ***C***H), 73.06 (3 C; Hub-***C***H_2_O), 70.54 (39 C; ***C***H_2_O), 70.36 (3 C; Ri ***C***H), 69.51 (3 C; ***C***H_2_O), 69.02 (3 C; ***C***H_2_O), 63.94 (3 C; ***C***H_2_O), 61.56 (3 C; 5′-***C***H_2_), 59.03 (3 C; 2′-O***C***H_3_), 55.26 (6 C; Dmtr O***C***H_3_), 28.88 (3 C; Suc ***C***H_2_), 28.83 ppm (3 C; Suc ***C***H_2_); MS (MALDI-ToF+): *m/z* calcd for [C_162_H_210_N_6_NaO_57_]^+^: 3175.4; found: 3176 [**3**+Na]^+^.

**Tris-1,3,5-[ω-(2′-*O*-methyluridine-3′-*O*-succinyloxy)octa(ethylene glycol)-α-oxymethyl]benzene (4)**: The crude tris(Dmtr-mU) homostar **3** (4.438 g from the above procedure) was dissolved in CH_2_Cl_2_ (40 mL) and pyrrole (1.39 mL, 19.6 mmol) was added. Dichloroacetic acid was then added in aliquots (0.40 mL, 4.8 mmol) until a strong orange colour remained, and then dissipated over 20 min; four aliquots were required to overwhelm buffering of the crude Dmtr ether and TLC confirmed complete unblocking of intermediate **3**. The reaction was diluted with CH_2_Cl_2_ (200 mL) and partitioned with sat. NaHCO_3_ (150 mL). The aqueous layer was back-extracted with CH_2_Cl_2_ (50 mL×4), organic layers combined, dried over Na_2_SO_4_ and the solvent stripped off under reduced pressure. The residue was fractionated through a column of silica gel (180 mL) in a large sinter funnel, eluting with a gradient of CH_3_OH–CHCl_3_ (2:98 to 11:89 v/v). Bands containing Dcb-ester **5** (409 mg), mixed with some product, and compound **4** (1.644 g, 80 %) were isolated as colourless gums. *R*_f_ (CH_3_OH–CHCl_3_ 1:9) **4** 0.35; **5** 0.51; ^1^H NMR (400 MHz, CDCl_3_): *δ*=9.53 (br d, *J*=2.3, 3 H; U 4-N***H***), 7.85 (d, *J*=8.1 Hz, 3 H; U 6-C***H***), 7.23 (s, 3 H; Hub C***H***), 5.85 (d, *J*=5.1 Hz, 3 H; 1′-C***H***), 5.76 (dd, *J*=8.0, 2.0 Hz, 3 H; U 5-C***H***), 5.32 (t, *J*=4.6 Hz, 3 H; 3′-C***H***) 4.55 (s, 6 H; Hub-C***H***_***2***_O), 4.26 (dt, *J*=5.4, 2.0 Hz, 6 H; Suc-OC***H***_***2***_), 4.22–4.19 (m, 6 H; 4′-C***H*** + 2′-C***H***), 3.94 (br d, *J*=12.0 Hz, 3 H; 5′-CH***H***), 3.79 (br dd, *J*=12.3, 4.0 Hz, 3 H; 5′-CH***H***), 3.72–3.61 (m, 90 H; C***H***_***2***_O), 3.43 (s, 9 H; 2′-OC***H***_***3***_), 2.76–2.68 ppm (m, 12 H; Suc C***H***_***2***_C***H***_***2***_); ^13^C NMR (101 MHz, CDCl_3_): *δ*=172.08 (3 C; Suc ***C***=O), 171.68 (3 C; Suc ***C***=O), 163.92 (3 C; U ***C***), 150.75 (3 C; U ***C***), 141.22 (3 C; U 6-***C***H), 138.51 (3 C; Hub ***C***), 126.29 (3 C; Hub ***C***H), 102.64 (3 C; U 5-***C***H), 88.69 (3 C; 1′-***C***H), 83.18 (3 C; 2′/4′-***C***H), 81.43 (3 C; 2′/4′-***C***H), 73.00 (3 C; Hub-***C***H_2_O), 70.96 (3 C; 3′-***C***H), 70.48 (39 C; ***C***H_2_O), 69.46 (3 C; ***C***H_2_O), 68.96 (3 C; ***C***H_2_O), 63.90 (3 C; SucO-***C***H_2_), 61.15 (3 C; 5′-***C***H_2_), 58.90 (3 C; 2′-O***C***H_3_), 28.95 ppm (6 C; Suc ***C***H_2_***C***H_2_); MS (MALDI-ToF+): *m/z* calcd for [C_99_H_156_N_6_NaO_51_]^+^: 2268.97; found: 2269.0 [**4**+Na]^+^.

**Tris-1,3,5-{ω-(2′-*O*-Me-5′-*O*-{[2′-*O*-Me-5′-*O*-(Dmtr)-4-*N-*acetylcytosin-3′-yl](2-cyanoethyloxy)thiophosphoryl}uridinyl-3′-*O*-succinyloxy)octa(ethylene glycol)-α-oxymethyl}benzene, tris(mUp^Cne^SmC^Ac^-ODmtr) homostar (7)**: Tris(2′-methyluridine) homostar **4** (422 mg, 0.188 mmol) and compound **6^C^** (678 mg, 0.846 mmol, 4.5 equiv) were co-evaporated from CH_3_CN (3×10 mL) in vacuo. To the residue was added 0.25 m ETT in CH_3_CN (6.77 mL, 1.69 mmol, 9 equiv), and after 40 min, PADS (1.71 g, 5.65 mmol, 30 equiv) and pyridine (6.8 mL) were added. After a further 60 min the solvent was stripped off under reduced pressure and the residue fractionated through a column of silanised silica, eluting with a gradient of water–THF, plus sat. NaHCO_3_ (0.5 vol %). The appropriate fractions were combined and the THF evaporated in vacuo. The resultant emulsion was extracted with CH_2_Cl_2_ (100 mL×4), the organic layer dried over Na_2_SO_4_, and then evaporated to dryness. The residue was fractionated through silanised silica, eluting with a gradient of water–CH_3_CN, plus sat. NaHCO_3_ (0.5 vol %). The appropriate fractions were extracted as before to afford compound **7** (538 mg, 64 %). *R*_f_ (CH_3_OH–CH_2_Cl_2_ 1:9) 0.41; intermediate tris(phosphite triester) 0.54; ^1^H NMR (400 MHz, D_6_-acetone): *δ*=10.38 (br s, 1.5 H; N***H***), 10.25 (s, 1.5 H; N***H***), 10.19 (s, 1.5 H; N***H***), 10.15 (br s, 1.5 H; N***H***), 8.49 (d, *J*=7.4 Hz, 3 H; C C***H***), 7.75 (d, *J*=8.2 Hz, 1.5 H; U C***H***), 7.64 (d, *J*=8.2 Hz, 1.5 H; U C***H***), 7.54–7.51 (m, 6 H; Dmtr C***H***), 7.42–7.36 (m, 18 H; Dmtr C***H***), 7.33–7.30 (m, 3 H; Dmtr C***H***), 7.28 (s, 3 H; Hub C***H***), 7.13 (d, *J*=7.2 Hz, 1.5 H; C C***H***), 7.12 (d, *J*=7.4 Hz, 1.5 H; C C***H***), 6.97–6.93 (m, 12 H; Dmtr C***H***), 6.07–6.04 (m, 3 H; 1′-C***H***), 5.99–5.96 (m, 3 H; 1′-C***H***), 5.71 (d, *J*=7.7 Hz, 1.5 H; U C***H***), 5.70 (d, *J*=7.8 Hz, 1.5 H; U C***H***), 5.37–5.26 (m, 2 H; 2×3′-C***H***), 4.56 (s, 6 H; Hub-C***H***_***2***_O), 4.53–4.37 (m, 9 H; U 4′-C***H*** + C 4′-C***H*** + POCH***H***CH_2_CN), 4.36–4.15 (m, 21 H; U 2′-C***H*** + C 2′-C***H*** + 5′-C***H***_***2***_ + POCH***H***CH_2_CN + Suc-OC***H***_***2***_), 3.84 (s, 9 H; Dmtr OC***H***_***3***_), 3.83 (s, 9 H; Dmtr OC***H***_***3***_), 3.69–3.53 (m, 105 H; C***H***_***2***_O + 5′-C***H***_***2***_ + 2′-OC***H***_***3***_), 3.41 (s, 4.5 H; 2′-OC***H***_***3***_), 3.40 (s, 4.5 H; 2′-OC***H***_***3***_), 3.01 (t, *J*=7.2 Hz, 3 H; C***H***_***2***_CN), 2.9–2.87 (m, 3 H; C***H***_***2***_CN), 2.76–2.73 (m, 6 H; Suc C***H***_***2***_), 2.70–2.67 (m, 6 H; Suc C***H***_***2***_), 2.25 (s, 4.5 H; Ac C***H***_***3***_), 2.24 ppm (s, 4.5 H; Ac C***H***_***3***_); ^13^C NMR (101 MHz, D_6_-acetone): *δ*=171.89 (3 C; ***C***=O), 171.45 (3 C; ***C***=O), 170.66 (3 C; ***C***=O), 163.09 (3 C; U/C ***C***), 162.68 (1.5 C; U/C ***C***), 162.64 (1.5 C; U/C ***C***), 158.92 (6 C; Dmtr ***C***), 154.71 (1.5 C; C ***C***), 154.60 (1.5 C; C ***C***), 150.47 (1.5 C; U ***C***), 150.44 (1.5 C; U ***C***), 144.44 (1.5 C; C ***C***H), 144.30 (1.5 C; C ***C***H), 144.27 (1.5 C; Dmtr ***C***), 144.12 (1.5 C; Dmtr ***C***), 140.02 (3 C; U 6-***C***H), 139.00 (3 C; Hub ***C***), 135.43 (1.5 C; Dmtr ***C***), 135.33 (1.5 C; Dmtr ***C***), 135.19 (1.5 C; Dmtr ***C***), 135.15 (1.5 C; Dmtr ***C***), 130.27 (6 C; Dmtr ***C***H), 130.18 (6 C; Dmtr ***C***H), 128.48 (3 C; Dmtr ***C***H), 128.40 (3 C; Dmtr ***C***H), 128.02 (6 C; Dmtr ***C***H), 127.18 (3 C; Dmtr ***C***H), 125.70 (3 C; Hub ***C***H), 117.59 (1.5 C; ***C***N), 117.31 (1.5 C; ***C***N), 113.27 (12 C; Dmtr ***C***H), 102.65 (1.5 C; U 5-***C***H), 102.52 (1.5 C; U 5-***C***H), 96.15 (3 C; C ***C***H), 88.80 (1.5 C; 1′-***C***H), 88.51 (1.5 C; 1′-***C***H), 88.04 (1.5 C; 1′-***C***H), 87.99 (1.5 C; 1′-***C***H), 87.08 (1.5 C; Dmtr ***C***), 87.06 (1.5 C; Dmtr ***C***), 82.09 (1.5 C; Ri ***C***H), 81.78 (1.5 C; Ri ***C***H), 80.91 (3 C; Ri ***C***H), 80.79 (3 C; Ri ***C***H), 80.15–80.00 (m, 3 C; Ri ***C***H), 73.51 (1.5 C; Ri ***C***H), 73.28 (1.5 C; Ri ***C***H), 72.54 (3 C; Hub-***C***H_2_O), 70.58 (3 C; Ri ***C***H), 70.35 (39 C; ***C***H_2_), 69.64 (3 C; ***C***H_2_O), 68.72 (3 C; ***C***H_2_O), 67.42–67.10 (3 C; U 5′-***C***H_2_), 63.75–63.48 (m, 6 C; ***C***H_2_O + PO***C***H_2_CH_2_CN), 60.57 (1.5 C; C 5′-***C***H_2_), 60.49 (1.5 C; C 5′-***C***H_2_), 58.40 (1.5 C; 2′-O***C***H_3_), 58.33 (1.5 C; 2′-O***C***H_3_), 58.13 (1.5 C; 2′-O***C***H_3_), 57.83 (1.5 C; 2′-O***C***H_3_), 54.81 (3 C; Dmtr O***C***H_3_), 54.79 (3 C; Dmtr O***C***H_3_), 28.67 (6 C; Suc ***C***H_2_), 24.13 (1.5 C; Ac ***C***H_3_), 24.05 (1.5 C; Ac ***C***H_3_), 19.07–18.90 ppm (m, 3 C; ***C***H_2_CN); ^31^P NMR (162 MHz, D_6_-acetone) *δ*=67.33 (0.39 P), 67.11 ppm (0.61 P); MS (MALDI-ToF+): *m/z* calcd for [C_207_H_269_N_18_NaO_79_P_3_S_3_]^+^: 4484.59; found: 4484.1 [**7**+Na+H_2_O]^+^.

**Tris(mUp^Cne^SmC^Ac^-OH) homostar (10):** Tris(DmtrO-2-mer) homostar **7** (192 mg, 43 μmol) was placed in CH_2_Cl_2_ (1 mL), to which pyrrole (35 μL) then DCA (35 μL) were added. After 60 min the reaction was complete by TLC and 1 m triethylammonium bicarbonate (TEAB, 0.5 mL) was added, followed by sufficient CH_3_CN to give a clear solution. The solution was concentrated at reduced pressure until all CH_2_Cl_2_ had evaporated, then silanised silica (10 mL) was added to the remaining solution followed by the slow addition of water (90 mL) with gentle swirling, plus 1 m TEAB (1 mL). The silica was collected in a glass sinter funnel, and the pad was washed with water (50 mL), then CH_3_CN–water (1:3 v/v, 160 mL plus 1.5 mL TEAB) and the filtrate was discarded. Next the silica was washed with CH_3_CN (150 mL) and the filtrate evaporated to dryness. The residue (156 mg) was taken up in CHCl_3_ (20 mL) and to the swirled solution was added normal phase silica (10 mL). The silica was collected in a glass sinter funnel, the pad was washed with further CHCl_3_ (130 mL) and the filtrate discarded. Finally the silica was washed with CH_3_OH–CHCl_3_ (1:9 v/v, 150 mL) and the filtrate evaporated to dryness to afford compound **10** (118 mg, 77 %). *R*_f_(EtOH–CHCl_3_ 1:9) 0.19; ^1^H NMR (500 MHz, CDCl_3_–CD_3_OD 2:1 v/v): *δ*=8.42 (d, *J*=7.5 Hz, 1.5 H; C C***H***), 8.41 (d, *J*=7.6 Hz, 1.5 H; C C***H***), 7.63 (d, *J*=8.1 Hz, 1.5 H; U C***H***), 7.59 (d, *J*=8.1 Hz, 1.5 H; U C***H***), 7.402 (d, *J*=7.5 Hz, 1.5 H; C C***H***), 7.397 (d, *J*=7.6 Hz, 1.5 H; C C***H***), 7.21 (s, 3 H; Hub C***H***), 5.98 (d, *J*=3.7 Hz, 3 H; Ri 1′-C***H***), 5.91 (d, *J*=4.7 Hz, 1.5 H; Ri 1′-C***H***), 5.90 (d, *J*=4.9 Hz, 1.5 H; Ri 1′-C***H***), 5.75 (d, *J*=8.1 Hz, 1.5 H; U C***H***), 5.72 (d, *J*=8.1 Hz, 1.5 H; U C***H***), 5.21 (t, *J*=5.2 Hz, 3 H; U 3′-C***H***) 5.03–4.97 (m, 3 H; C 3′-C***H***), 4.52 (s, 6 H; Hub-C***H***_***2***_O), 4.45–4.23 (m, 18 H; POC***H***_***2***_CH_2_CN + U 5′- C***H***_***2***_ + C 4′-C***H*** + U 4′-C***H***), 4.21–4.19 (m, 6 H; Suc-OC***H***_***2***_), 4.15 (br t, *J*=4.3 Hz, 1.5 H; U 2′-C***H***), 4.14 (br t, *J*=4.4 Hz, 1.5 H; U 2′-C***H***), 4.04 (dd, *J*=4.9, 1.3 Hz, 1.5 H; C 2′-C***H***), 4.03 (dd, *J*=5.3, 1.3 Hz, 1.5 H; C 2′-C***H***), 3.95–3.91 (m, 3 H; 5′-CH***H***), 3.78–3.73 (m, 3 H; 5′-CH***H***), 3.67 (t, *J*=4.8 Hz, 6 H; C***H***_***2***_O), 3.65–3.59 (m, 84 H; C***H***_***2***_O), 3.53 (s, 4.5 H; 2′-OC***H***_***3***_), 3.50 (s, 4.5 H; 2′-OC***H***_***3***_), 3.39 (s, 9 H; 2′-OC***H***_***3***_), 2.85–2.82 (m, 6 H; C***H***_***2***_CN), 2.72–2.69 (m, 6 H; Suc C***H***_***2***_C***H***_***2***_), 2.67–2.64 (m, 6 H; Suc C***H***_***2***_C***H***_***2***_), 2.17 ppm (s, 9 H; Ac C***H***_***3***_); ^13^C NMR (126 MHz, CDCl_3_–CD_3_OD 2:1 v/v): *δ*=172.40 (3 C; ***C***=O), 171.75 (1.5 C; ***C***=O), 171.71 (1.5 C; ***C***=O), 171.56 (3 C; ***C***=O), 164.15 (3 C; U/C ***C***), 162.83 (3 C; U/C ***C***), 156.09 (3 C; C ***C***), 150.52 (3 C; U ***C***), 145.39 (3 C; C ***C***H), 140.21 (3 C; U 6-***C***H), 138.47 (3 C; Hub ***C***), 126.40 (3 C; Hub ***C***H), 117.03 (1.5 C; ***C***N), 116.96 (1.5 C; ***C***N), 102.79 (1.5 C; U 5-***C***H), 102.73 (1.5 C; U 5-***C***H), 97.40 (1.5 C; C ***C***H), 97.34 (1.5 C; C ***C***H), 89.22 (1.5 C; C 1′-***C***H), 89.10 (1.5 C; C 1′-***C***H), 88.08 (1.5 C; U 1′-***C***H), 88.02 (1.5 C; U 1′-***C***H), 83.29–83.19 (m, 3 C; U 4′-***C***H), 82.17 (3 C; C 2′-***C***H), 81.09 (1.5 C; U 2′-***C***H), 81.04 (1.5 C; U 2′-***C***H), 80.08–79.97 (m, 3 C; C 4′-***C***H), 74.50–74.42 (m, 3 C; C 3′-***C***H), 72.93 (3 C; Hub-***C***H_2_O), 70.35 (39 C; ***C***H_2_O), 70.18 (3 C; U 3′-***C***H), 69.43 (3 C; ***C***H_2_O), 68.87 (3 C; ***C***H_2_O), 66.70 (br, 3 C; U 5′-***C***H_2_), 63.91 (3 C; SucO***C***H_2_), 63.14–63.07 (m, 3 C; PO***C***H_2_CH_2_CN), 59.65 (3 C; C 5′-***C***H_2_), 58.81 (3 C; 2′-O***C***H_3_), 58.57 (1.5 C; 2′-O***C***H_3_), 58.54 (1.5 C; 2′-O***C***H_3_), 28.76 (6 C; Suc ***C***H_2_), 24.24 (3 C; Ac ***C***H_3_), 19.22–19.10 ppm (m, 3 C, ***C***H_2_CN); ^31^P NMR (202 MHz, CDCl_3_–CD_3_OD 2:1 v/v): *δ*=67.81 (0.44 P), 67.59 ppm (0.56 P); MS (MALDI-ToF+): *m/z* calcd for [C_144_H_214_N_18_NaO_72_P_3_S_3_]^+^=3537.20; found: 3538.3 [**10**+H]^+^.

**Tris(mUp^Cne^SmC^Ac^p^Cne^SmC^Ac^p^Cne^SmA^Bz^p^Cne^SmUp^Cne^SmU-OH) homostar (21)—typical chain extension cycle:** Tris(HO-5-mer) homostar **18** (1.286 g, 0.171 mmol) was dissolved in DMF (4 mL) to which was added CH_3_CN (20 mL) and the solution evaporated in vacuo; this was repeated with DMF (2 mL) plus CH_3_CN (20 mL), and finally with neat CH_3_CN (20 mL). Phosphoramidite **6^U^** (585 mg, 0.769 mmol, 4.5 equiv) was then added to the residue followed by 0.25 m ETT in CH_3_CN (6.2 mL, 1.55 mmol, 9 equiv). After 35 min PADS (465 mg, 1.55 mmol, 9 equiv) and pyridine (6.2 mL) were added, and after a further 35 min the reaction was diluted into CH_3_OH–CH_3_CN (3:17 v/v) and poured into the OSN rig. Once 12 diavolumes had permeated, the retentate was evaporated to give crude 5′-Dmtr homostar **20** (1.779 g) as a brown glass.

Partially purified tris(DmtrO-6-mer) homostar **20** (1.708 g) was placed in CH_2_Cl_2_ (28 mL), to which was added pyrrole (0.48 mL) then DCA (0.28 mL). After 45 min the reaction was complete by TLC and pyridine (0.28 mL) was added. The mixture was diluted with CH_3_CN (100 mL) and the liquid concentrated until all the CH_2_Cl_2_ had evaporated. To this solution was then added CH_3_OH (20 mL), the solution was diluted with further CH_3_OH–CH_3_CN (3:17 v/v) containing pyridinium.DCA (0.5 vol %) and 5 diavolumes were permeated. The flux was observed to drop significantly, so this was followed by 10 diavolumes CH_3_OH–CH_3_CN (1:4 v/v) when the flux improved. The retentate was evaporated to dryness, and the residual glass was re-dissolved in CH_2_Cl_2_–CH_3_OH (10 mL). The solution was added dropwise to briskly stirred diethyl ether (300 mL), and the precipitate collected to afford tris(HO-6-mer) homostar **21** (1.394 g, 98 %) as a brown powder. ^31^P NMR (202 MHz, CDCl_3_-CD_3_OD 2:1 v/v): *δ*=67.8–67.1 ppm (m, 15 P); MS (MALDI-ToF+): *m/z* calcd for [C_330_H_429_N_66_NaO_153_P_15_S_15_]^+^: 8734.0; found: 8728 [**21**+Na]^+^.

## Supporting Information

As a service to our authors and readers, this journal provides supporting information supplied by the authors. Such materials are peer reviewed and may be re-organized for online delivery, but are not copy-edited or typeset. Technical support issues arising from supporting information (other than missing files) should be addressed to the authors.

miscellaneous_information
