# Supplementary material for: Liquid-Phase Synthesis of 2′-Methyl-RNA on a Homostar Support through Organic-Solvent Nanofiltration
Source: Chemistry. 2015 May 26;21(26):9535–43. doi: 10.1002/chem.201501001 (PMC4517100; doi:10.1002/chem.201501001)
Supplement: Supplementary file 1 — miscellaneous_information [file chem0021-9535-sd1.pdf]

CHEMISTRY
A European Journal

Supporting Information

Liquid-Phase Synthesis of 2′-Methyl-RNA on a Homostar Support through Organic-Solvent Nanofiltration

Piers R. J. Gaffney,[a] Jeong F. Kim,[a] Irina B. Valtcheva,[a] Glynn D. Williams,[b] Mike S. Anson,[b] Andrew M. Buswell,[b] and Andrew G. Livingston*[a]

chem_201501001_sm_miscellaneous_information.pdf

Liquid-phase Synthesis of RNA on a Homostar Support via Organic Solvent Nanofiltration

Supplementary Information

Piers R. J. Gaffney, Jeong F. Kim, Irina Valtcheva, Glynn D. Williams,

Mike S. Anson, Andrew M. Buswell and Andrew G. Livingston

Contents

1. Synthetic Procedures – Chromatographic Purifications

1.1 2′-*O*-Methyl-3′-O-succinyl-5′-*O*-(4″,4‴-Dimethoxytriphenylmethyl)uridine, 2.....................4

1.2 Bis-1,3-[ω-(2′-*O*-methyluridine-3′-*O*-succinyloxy)octa(ethylene glycol)-α-oxymethyl]-5-[ω-(2,6-dichlorobenzoylox)octa(ethylene glycol)-α-oxymethyl]benzene, 5...4

1.3 2′-*O*-Methyl-3′-*O*-(2-cyanoethyloxyphosphorodithioyl)-5′-*O*-(4″,4‴-dimethoxytriphenylmethyl)-4-*N*-acetyl cytidine, triethylammonium salt, 8a^{C}..5

1.4 2′-*O*-Methyl-3′-*O*-[di(2-cyanoethyloxy)phosphorothioyl]-5′-*O*-(4″,4‴-dimethoxytriphenylmethyl)-4-*N*-acetyl cytidine and 2′-*O*-methyl-3′-*O*-(2-cyanoethyloxyphosphorothioyl)-5′-*O*-(4″,4‴-dimethoxytriphenylmethyl)-4-*N*-acetyl cytidine, triethylammonium salt, 8b^{C}............................5

1.5 2′-*O*-Methyl-5′-*O*-(4″,4‴-dimethoxytriphenylmethyl)-4-*N*-acetyl cytidine 3′-*O*-(*N,N*-diisopropyl)(2-cyanoethyl)phosphorothioamidate, 9a^{C}..6

1.6 2′-*O*-Methyl-5′-*O*-(4″,4‴-dimethoxytriphenylmethyl)-4-*N*-acetyl cytidine 3′-*O*-(*N,N*-diisopropyl)(2-cyanoethyl)phosphorothioamidate, 9b^{C}..7

2. Synthetic Procedures – Purifications by Organic Solvent Nanofiltration

2.1 Organic Solvent Nanofiltration Apparatus..9

2.2 Tris(mUp^{Cne}SmC^{Ac}-OH) homostar, 10..9

2.3 Tris(mUp^{Cne}SmC^{Ac}p^{Cne}SmC^{Ac}-OH) homostar, 14..9

2.4 Tris(mUp^{Cne}SmC^{Ac}p^{Cne}SmC^{Ac}p^{Cne}SmA^{Bz}-OH) homostar, 16...10

2.5 Tris(mUp^{Cne}SmC^{Ac}p^{Cne}SmC^{Ac}p^{Cne}SmA^{Bz}p^{Cne}SmU-OH) homostar, 18....................................10

2.6 HO-mUpSmCpSmCpSmApSmU-OH, 19...11

2.7 Tris(mUp^{Cne}SmC^{Ac}p^{Cne}SmC^{Ac}p^{Cne}SmA^{Bz}p^{Cne}SmUp^{Cne}SmU-OH) homostar, 21.................... 11

2.8 Tris(mUp^{Cne}SmC^{Ac}p^{Cne}SmC^{Ac}p^{Cne}SmA^{Bz}p^{Cne}SmUp^{Cne}SmUp^{Cne}SmC^{Ac}-OH) homostar, 23.. 11

2.9 Tris(mUp^{Cne}SmC^{Ac}p^{Cne}SmC^{Ac}p^{Cne}SmA^{Bz}p^{Cne}SmUp^{Cne}SmUp^{Cne}SmC^{Ac}p^{Cne}SmG^{iBu}-OH) homostar, 25..12

2.10 Tris(mUp^{Cne}SmC^{Ac}p^{Cne}SmC^{Ac}p^{Cne}SmA^{Bz}p^{Cne}SmUp^{Cne}SmUp^{Cne}SmC^{Ac}p^{Cne}SmG^{iBu}p^{Cne}SmG^{iBu}-OH) homostar, 27.. 13

2.11 HO-mUpSmCpSmCpSmApSmUpSmUpSmCpSmGpSmG-OH, 28....................................13

3. ^{1}H & ^{13}C NMR Spectra of Chromatographically Purified Materials

3.1 2′-*O*-Methyl-3′-O-succinyl-5′-*O*-(4″,4‴-dimethoxytriphenylmethyl)uridine, triethylammonium salt, 2 14
3.2 Tris(mU-ODmtr) homostar, 3 15
3.3 Tris(mU-OH) homostar, 4 16
3.4 Bis(mU-OH)Dcb homostar, 5 17
3.5 Tris(mUp^{Cne}SmC^{Ac}-ODmtr) homostar, 7 18
3.6 Tris(mUp^{Cne}SmC^{Ac}-OH) homostar, 10 20

4. ^{1}H & ^{31}P NMR Spectra of Homostars Purified by OSN

4.1 Tris(mUp^{Cne}SmC^{Ac}-ODmtr) homostar, 7 22
4.2 Tris(mUp^{Cne}SmC^{Ac}-OH) homostar, 10 23
4.3 Tris(mUp^{Cne}SmC^{Ac}p^{Cne}SmC^{Ac}-ODmtr) homostar, 13 24
4.4 Tris(mUp^{Cne}SmC^{Ac}p^{Cne}SmC^{Ac}-OH) homostar, 14 25
4.5 Tris(mUp^{Cne}SmC^{Ac}p^{Cne}SmC^{Ac}p^{Cne}SmA^{Bz}-ODmtr) homostar, 15 26
4.6 Tris(mUp^{Cne}SmC^{Ac}p^{Cne}SmC^{Ac}p^{Cne}SmA^{Bz}-OH) homostar, 16 27
4.7 Tris(mUp^{Cne}SmC^{Ac}p^{Cne}SmC^{Ac}p^{Cne}SmA^{Bz}p^{Cne}SmU-ODmtr) homostar, 17 28
4.8 Tris(mUp^{Cne}SmC^{Ac}p^{Cne}SmC^{Ac}p^{Cne}SmA^{Bz}p^{Cne}SmU-OH) homostar, 18 29
4.9 Tris(mUp^{Cne}SmC^{Ac}p^{Cne}SmC^{Ac}p^{Cne}SmA^{Bz}p^{Cne}SmUp^{Cne}SmU-ODmtr) homostar, 20 30
4.10 Tris(mUp^{Cne}SmC^{Ac}p^{Cne}SmC^{Ac}p^{Cne}SmA^{Bz}p^{Cne}SmUp^{Cne}SmU-OH) homostar, 21 31
4.11 Tris(mUp^{Cne}SmC^{Ac}p^{Cne}SmC^{Ac}p^{Cne}SmA^{Bz}p^{Cne}SmUp^{Cne}SmUp^{Cne}SmC^{Ac}-ODmtr) homostar, 22 32
4.12 Tris(mUp^{Cne}SmC^{Ac}p^{Cne}SmC^{Ac}p^{Cne}SmA^{Bz}p^{Cne}SmUp^{Cne}SmUp^{Cne}SmC^{Ac}-OH) homostar, 23 33
4.13 Tris(mUp^{Cne}SmC^{Ac}p^{Cne}SmC^{Ac}p^{Cne}SmA^{Bz}p^{Cne}SmUp^{Cne}SmUp^{Cne}SmC^{Ac}p^{Cne}SmG^{iBu}-ODmtr) homostar, 24 34
4.14 Tris(mUp^{Cne}SmC^{Ac}p^{Cne}SmC^{Ac}p^{Cne}SmA^{Bz}p^{Cne}SmUp^{Cne}SmUp^{Cne}SmC^{Ac}p^{Cne}SmG^{iBu}-OH) homostar, 25 35
4.15 Tris(mUp^{Cne}SmC^{Ac}p^{Cne}SmC^{Ac}p^{Cne}SmA^{Bz}p^{Cne}SmUp^{Cne}SmUp^{Cne}SmC^{Ac}p^{Cne}SmG^{iBu}p^{Cne}SmG^{iBu}-ODmtr) homostar, 26 36
4.16Tris(mUp^{Cne}SmC^{Ac}p^{Cne}SmC^{Ac}p^{Cne}SmA^{Bz}p^{Cne}SmUp^{Cne}SmUp^{Cne}SmC^{Ac}p^{Cne}SmG^{iBu}p^{Cne}SmG^{iBu}-OH) homostar, 27 37

5. Mass Spectra of Homostars and of Fully Unblocked 9-mer 28

5.1 Tris(mU-ODmtr) homostar, 3 38
5.2 Tris(mU-OH) homostar, 4 39
5.3 Bis(mU-OH)Dcb homostar, 5 40
5.4 Tris(mUp^{Cne}SmC^{Ac}-ODmtr) homostar, 7 41
5.5 Tris(mUp^{Cne}SmC^{Ac}-OH) homostar, 10 42
5.6 LC-MS of tris(mUp^{Cne}SmC^{Ac}-OH) homostar, 10 43
5.7 Tris(mUp^{Cne}SmC^{Ac}p^{Cne}SmC^{Ac}-ODmtr) homostar, 13 44
5.8 Tris(mUp^{Cne}SmC^{Ac}p^{Cne}SmC^{Ac}-OH) homostar, 14 45
5.9 Tris(mUp^{Cne}SmC^{Ac}p^{Cne}SmC^{Ac}p^{Cne}SmA^{Bz}-ODmtr) homostar, 15 46
5.10 Tris(mUp^{Cne}SmC^{Ac}p^{Cne}SmC^{Ac}p^{Cne}SmA^{Bz}-OH) homostar, 16 47
5.11 Tris(mUp^{Cne}SmC^{Ac}p^{Cne}SmC^{Ac}p^{Cne}SmA^{Bz}p^{Cne}SmU-ODmtr) homostar, 17 48
5.12 Tris(mUp^{Cne}SmC^{Ac}p^{Cne}SmC^{Ac}p^{Cne}SmA^{Bz}p^{Cne}SmU-OH) homostar, 18 49
5.13 LC-MS of crude HO-mUpSmCpSmCpSmApSmU-OH, 19 50
5.14 Tris(mUp^{Cne}SmC^{Ac}p^{Cne}SmC^{Ac}p^{Cne}SmA^{Bz}p^{Cne}SmUp^{Cne}SmU-ODmtr) homostar, 20 51
5.15 Tris(mUp^{Cne}SmC^{Ac}p^{Cne}SmC^{Ac}p^{Cne}SmA^{Bz}p^{Cne}SmUp^{Cne}SmU-OH) homostar, 21 52
5.16 Tris(mUp^{Cne}SmC^{Ac}p^{Cne}SmC^{Ac}p^{Cne}SmA^{Bz}p^{Cne}SmUp^{Cne}SmUp^{Cne}SmC^{Ac}-ODmtr) homostar, 22 53

5.17 Tris(mUp^{Cne}SmC^{Ac}p^{Cne}SmC^{Ac}p^{Cne}SmA^{Bz}p^{Cne}SmUp^{Cne}SmUp^{Cne}SmC^{Ac}-OH) homostar, 23....54
5.18 Tris(mUp^{Cne}SmC^{Ac}p^{Cne}SmC^{Ac}p^{Cne}SmA^{Bz}p^{Cne}SmUp^{Cne}SmUp^{Cne}SmC^{Ac}p^{Cne}SmG^{iBu}-ODmtr) homostar, 24....55
5.19 Tris(mUp^{Cne}SmC^{Ac}p^{Cne}SmC^{Ac}p^{Cne}SmA^{Bz}p^{Cne}SmUp^{Cne}SmUp^{Cne}SmC^{Ac}p^{Cne}SmG^{iBu}-OH) homostar, 25....56
5.20 Tris(mUp^{Cne}SmC^{Ac}p^{Cne}SmC^{Ac}p^{Cne}SmA^{Bz}p^{Cne}SmUp^{Cne}SmUp^{Cne}SmC^{Ac}p^{Cne}SmG^{iBu}p^{Cne}SmG^{iBu}-ODmtr) homostar, 26....57
5.21 Tris(mUp^{Cne}SmC^{Ac}p^{Cne}SmC^{Ac}p^{Cne}SmA^{Bz}p^{Cne}SmUp^{Cne}SmUp^{Cne}SmC^{Ac}p^{Cne}SmG^{iBu}p^{Cne}SmG^{iBu}-OH) homostar, 27....58
5.22 LC-MS of purified HO-mUpSmCpSmCpSmApSmUpSmUpSmCpSmGpSmG-OH, 28....59

6. HPLC of Homostars....60

Synthetic Procedures – Chromatographic Purifications

1.1 2′-*O*-Methyl-3′-O-succinyl-5′-*O*-(4″,4‴-dimethoxytriphenylmethyl)uridine, triethyl-ammonium salt, 2.

2′-*O*-Methyl uridine (2.480 g, 9.60 mmol) was dissolved in DMF (15 mL), to which was added Et_3N (5.35 mL, 38.4 mmol) then solid 9-chloro-4,4′-dimethoxytriphenylmethane (DmtrCl, 3.901 g, 11.5 mmol) in four portions over 30 min; for the first three portions the orange colour dissipates completely after a few minutes, but after the last portion the colour persists. After a further 90 min solid succinic anhydride (1.442 g, 14.4 mmol) and *N*-methylimidazole (0.38 mL, 4.8 mmol) were added and the reaction stirred over night. The next day water (2.5 mL) was added, and after 20 min the solution was poured onto silanised silica (130 mL), washing out the flask with sufficient MeCN (*ca.* 100 mL) to fully wet the silica. The thick slurry was slowly diluted with water, with continual gentle swirling, making the volume up to 800 mL. The silica was collected in a large glass sinter funnel and thoroughly washed with water (800 mL), sucking dy the silica and discarding the filtrate. The silica was then washed with MeCN-water (19:1 v/v, 700 mL) and the filtrate concetrated *in vacuo*. The residue was re-evaporated with MeCN (3 × 100 mL) and re-dissolved in $CHCl_3$ (200 mL) containing a little Et_3N. This solution was drawn into a pad of silica gel (190 mL) in a large sinter funnel which was washed with EtOH-$CHCl_3$ (1:49 v/v, 500 mL + 1 mL Et_3N), discarding the filtrate. The silica was then washed with MeOH-$CHCl_3$ (1:4 v/v, 700 mL + 1 mL Et_3N) and the filtrate evaporated to dryness to afford the *title compound* (6.133 g, 83.9%) as a cream-coloured foam.

R_f(MeOH-$CHCl_3$ 1:9 + trace Et_3N) 0.40.

^{1}H NMR (400 MHz, $CDCl_3$) δ 7.93 (d, *J* = 8.1 Hz, 1H, U C***H***), 7.37 (d, *J* = 7.4 Hz, 2H, Dmtr C***H***), 7.32 (t, *J* = 7.5 Hz, 2H, Dmtr C***H***), 7.29 – 7.25 (m, 5H, Dmtr C***H***), 6.86 (dd, *J* = 8.8, 1.4 Hz, 4H, Dmtr C***H***), 6.02 (d, *J* = 3.3 Hz, 1H, 1′-C***H***), 5.33 (d, *J* = 8.2 Hz, 1H, U C***H***), 5.31 (t, *J* = 5.7 Hz, 1H, 3′-C***H***), 4.28 (bdt, *J* = 6.2, 2.3 Hz, 1H, 4′-C***H***), 4.10 (dd, *J* = 5.0, 3.5 Hz, 1H, 2′-C***H***), 3.81 (s, 6H, Dmtr OC$\boldsymbol{H}_3$), 3.57 (dd, *J* = 11.3, 2.4 Hz, 1H, 5′-CH***H***), 3.49 (s, 3H, 2′-OC$\boldsymbol{H}_3$), 3.47 (dd, *J* = 10.9, 2.0, 1H, 5′-CH***H***), 3.00 (q, *J* = 7.3 Hz, $CH_3C\boldsymbol{H}_2N^+$), 2.74 – 2.67 (m, 2H, Suc C$\boldsymbol{H}_2$), 2.62 – 2.59 (m, 2H, Suc C$\boldsymbol{H}_2$), 1.19 (t, *J* = 7.3 Hz, $C\boldsymbol{H}_3CH_2N^+$).

^{13}C NMR (101 MHz, $CDCl_3$) δ = ^{13}C NMR (101 MHz, $CDCl_3$) δ 178.26 (b, Suc ***C***=O), 173.07 (Suc ***C***=O), 163.64 (b, U ***C***), 158.67 (2C, Dmtr ***C***), 150.88 (b, U ***C***), 144.16 (Dmtr ***C***), 139.51 (U ***C***H), 135.15 (Dmtr ***C***), 134.94 (Dmtr ***C***), 130.10 (2C, Dmtr ***C***H), 130.06 (2C, Dmtr ***C***H), 128.09 (2C, Dmtr ***C***H), 128.03(2C, Dmtr ***C***H), 127.16 (Dmtr ***C***H), 113.31 (4C, Dmtr ***C***H), 102.67 (U ***C***H), 87.24 (Dmtr ***C***), 86.71 (b, Ri ***C***H), 82.43 (b, Ri ***C***H), 81.34 (b, Ri ***C***H), 70.06 (b, Ri ***C***H), 62.07 (5′-$\boldsymbol{C}H_2$), 59.08 (2′-O$\boldsymbol{C}H_3$), 55.23 (2C, Dmtr O$\boldsymbol{C}H_3$), 45.26 (Me$\boldsymbol{C}H_2$N), 32.02 (Suc $\boldsymbol{C}H_2$), 30.77 (Suc $\boldsymbol{C}H_2$), 9.87 ($\boldsymbol{C}H_3CH_2N$).

m/z (ESI−) [**2**−Et_3NH]$^-$ = 659.2228, calc. $C_{35}H_{35}N_2O_{11}^-$ = 659.2246; [**2**−$Et_3NH+HCO_2H$]$^-$ = 705.1960.

1.2 Bis-1,3-[ω-(2′-*O*-methyluridine-3′-*O*-succinyloxy)octa(ethylene glycol)-α-oxymethyl]-5-[ω-(2,6-dichlorobenzoylox)octa(ethylene glycol)-α-oxymethyl]benzene, 5.

^{1}H NMR (400 MHz, $CDCl_3$) δ 9.66 – 9.61 (m, 2H, U 4-N***H***), 7.84 (d, *J* = 8.1 Hz, 2H, U 6-C***H***), 7.33 – 7.25 (m, 3H, Dcb C***H***), 7.22 (s, 3H, Hub C***H***), 5.84 (d, *J* = 5.0 Hz, 2H, 1′-C***H***), 5.73 (d, *J* = 7.9 Hz, 2H, U 5-C***H***), 5.29 (t, *J* = 4.7 Hz, 2H, 3′-C***H***), 4.53 (s, 6H, Hub-C$\boldsymbol{H_2}$O), 4.26 (dt, *J* = 4.8, 1.3 Hz, 4H, Suc-OC$\boldsymbol{H_2}$), 4.20 – 4.16 (m, 4H, 4′-C***H*** + 2′-C***H***), 3.94 – 3.88 (m, 2H, 5′-CH***H***), 3.83 – 3.81 (m, 2H, Dcb-OC$\boldsymbol{H_2}$), 3.79 – 3.74 (m, 2H, 5′-CH***H***), 3.69 – 3.60 (m, Lots H, C$\boldsymbol{H_2}$O), 3.44 (s, 6H, 2′-OC$\boldsymbol{H_3}$), 2.74 – 2.64 (m, 8H, Suc C$\boldsymbol{H_2}$C$\boldsymbol{H_2}$).

^{13}C NMR **5** (101 MHz, $CDCl_3$) δ 172.15 (2C, Suc ***C***=O), 171.75 (2C, Suc ***C***=O), 164.70 (Dcb ***C***=O), 163.47 (2C, U ***C***), 150.75 (2C, U ***C***), 141.31 (2C, U 6-***C***H), 138.53 (3C, Hub ***C***), 133.41 (Dcb ***C***), 131.88 (2C, Dcb ***C***), 130.98 (Dcb ***C***H), 127.86 (2C, Dcb ***C***H), 126.33 (3C, Hub ***C***H), 102.65 (2C,

U 5-***C***H), 88.98 (2C, 1′-***C***H), 83.11 (2C, 2′/4′-***C***H), 81.37 (2C, 2′/4′-***C***H), 73.03 (3C, Hub-***C***H_2O), 70.78 (2C, 3′-***C***H), 70.63 – 70.49 (38C, ***C***H_2O), 69.48 (3C, ***C***H_2O), 68.97 (3C, ***C***H_2O), 68.77 (DcbO-CH_2***C***H_2-O), 65.24 (DcbO-***C***H_2), 63.94 (2C, SucO-***C***H_2), 61.22 (2C, 5′-***C***H_2), 58.93 (2C, 2′-O***C***H_3), 29.97 (8C, Suc ***C***H_2***C***H_2).

m/z (MALDI-ToF+) [**5**+Na]$^+$ = 2102, calc. $C_{92}H_{142}Cl_2N_4NaO_{44}^+$ = 2099.8.

1.3 2′-*O*-Methyl-3′-*O*-(2-cyanoethyloxyphosphorodithioyl)-5′-*O*-(4″,4‴-dimethoxytriphenylmethyl)-4-*N*-acetyl cytidine, triethylammonium salt, 8a$^{\mathbf{C}}$.

2′-*O*-Methyl-5′-*O*-(4″,4‴-dimethoxytriphenylmethyl)-4-*N*-acetyl cytidine 3′-*O*-(2-cyanoethyl)-*N*,*N*-diisopropyl phosphoramidite (**6**$^{\mathbf{C}}$, 188 mg, 0.234 mmol) was dissolved in 0.25 M ETT in MeCN (1.9 mL, 0.475 mmol). After stirring for 30 min PADS (708 mg, 2.34 mmol) then pyridine (1.9 mL) were added and after 1h the solvent was stripped off *in vacuo*. The residue was re-dissolved in MeCN (10 mL) to which was added silanized silica (20 mL) then slowly water (150 mL), with gentle swirling, and sat. $NaHCO_3$ (10 mL). The silica was washed with water (200 mL), then 1M triethylammonium bicarbonate-acetone-water (2:5:15 v/v/v, 220 mL), and the filtrate discarded. The silica was finally washed with acetone and the solvent was stripped off. The crude material was fractionated through a column of silanized silica (120 mL), eluting with a gradient of MeCN-water (2:8 to 7:3), including 1% sat. $NaHCO_3$, and the appropriate fractions (MeCN-water 4:6) were concentrated under reduced pressure until all MeCN had evaporated; the later fractions (MeCN-water 7:3) contained Dmtr-thioamidate (**9a**$^{\mathbf{C}}$), the isolation of which is described in section **1.6**. To the clear aqueous solution was added 1M triethylammonium bicarbonate, and the now cloudy emulsion was extracted with dichloromethane (×6) until the cloudiness had dissipated. The combined organic layers were dried over Na_2SO_4, the solvent evaporated *in vacuo*, and the residue co-evaporated from dichloromethane-EtOH until the mass remained constant, to give the *title compound* (98 mg, *ca.* 48%) containing *ca.* 10% mono-thioate **8b**$^{\mathbf{C}}$.

R_f(MeOH-CH_2Cl_2 1:9) 0.11.

^{1}H NMR (400 MHz, $CDCl_3$) δ 10.05 (bs, 1H, N***H***), 8.45 (d, *J* = 7.5 Hz, 1H, C C***H***), 7.47 (d, *J* = 7.3 Hz, 2H, Dmtr C***H***), 7.37 – 7.24 (m, 7H, Dmtr C***H***), 6.92 (d, *J* = 7.5 Hz, 1H, C C***H***), 6.86 (d, *J* = 8.1 Hz, 4H, Dmtr C***H***), 6.06 (d, *J* = 1.8 Hz, 1H, 1′-C***H***), 5.18 (ddd, *J* = 12.5, 7.9, 4.7 Hz, 1H, 3′-C***H***), 4.45 (bd, *J* = 7.8 Hz, 1H, 4′-C***H***), 4.20 – 4.03 (m, 3H, POC***H₂***CH2CN + 2′-C***H***), 3.81 (s, 6H, Dmtr OC***H₃***), 3.65 (s, 3H, 2′-OC***H₃***), 3.63 (bs, 2H, 5′-C***H₂***), 3.09 (q, *J* = 7.3 Hz, CH_3C***H₂***N^+), 2.68 (t, *J* = 6.6 Hz, 2H, C***H₂***CN), 1.24 (t, *J* = 7.2 Hz, C***H₃***CH_2N^+).

^{13}C NMR (101 MHz, $CDCl_3$) δ = ^{13}C NMR (101 MHz, $CDCl_3$) δ 170.84 (Ac ***C***=O), 162.87 (C ***C***), 158.61 (2C, Dmtr ***C***), 155.22 (C ***C***), 144.98 (Dmtr ***C***), 144.27 (C ***C***H), 135.47 (Dmtr ***C***), 135.17 (Dmtr ***C***), 130.45 (4C, Dmtr ***C***H), 128.53 (2C, Dmtr ***C***H), 127.97 (2C, Dmtr ***C***H), 127.09 (Dmtr ***C***H), 117.74 (***C***N), 113.24 (4C, Dmtr ***C***H), 96.85 (C ***C***H), 88.68 (1′-***C***H), 87.12 (Dmtr ***C***), 82.93 (Ri ***C***H), 81.48 (d, *J* = 7.7 Hz, Ri ***C***H), 71.25 (d, *J* = 6.5 Hz, Ri ***C***H), 61.00 (5′-***C***H_2), 60.35 (d, *J* = 6.6 Hz, PO***C***H_2CH2CN), 58.61 (2′-O***C***H_3), 55.26 (2C, Dmtr O***C***H_3), 46.12 (Me***C***H_2N^+), 24.93 (Ac ***C***H_3), 19.42 (d, *J* = 7.6 Hz, ***C***H_2CN), 9.09 (***C***$H_3CH_2N^+$).

^{31}P NMR (162 MHz, $CDCl_3$) δ 114.42 (1P).

m/z (ESI−) [**8a**$^{\mathbf{C}}$−Et_3NH]$^-$ = 765.1804, calc. $C_{36}H_{38}N_4O_9PS_2^-$ = 765.1818.

1.4 2′-*O*-Methyl-3′-*O*-[di(2-cyanoethyloxy)phosphorothioyl]-5′-*O*-(4″,4‴-dimethoxytriphenylmethyl)-4-*N*-acetyl cytidine and 2′-*O*-methyl-3′-*O*-(2-cyanoethyloxyphosphorothioyl)-5′-*O*-(4″,4‴-dimethoxytriphenylmethyl)-4-*N*-acetyl cytidine, triethylammonium salt, 8b$^{\mathbf{C}}$.

2′-*O*-Methyl-5′-*O*-(4″,4‴-dimethoxytriphenylmethyl)-4-*N*-acetyl cytidine 3′-*O*-(2-cyanoethyl)-*N*,*N*-diisopropyl phosphoramidite (**6**$^{\mathbf{C}}$, 802 mg, 1.00 mmol) and 3-hydroxypropionitrile (102 μL, 1.5 mmol) were dissolved in 0.25 M ETT in MeCN (8.0 mL, 2 mmol). After 25 min PADS (600 mg, 2.0 mmol) then pyridine (8 mL) were added, and after a further 35 min the solvent was stripped off *in vacuo*. The residue was partitioned between CH_2Cl_2 and sat. $NaHCO_3$, the aqueous layer back-

extracted with further CH_2Cl_2, and the combined organic layers dried over Na_2SO_4. The solution of crude material was fractionated through silica gel (40 mL) in a medium diameter sinter funnel, eluting with a gradient of EtOH-CH_2Cl_2 (0:1 to 3:47 v/v), to afford the intermediate dicyanoethyl phosphorothioate triester (706 mg, 87%).

R_f(EtOH-CH_2Cl_2 1:9) 0.47.

^{1}H NMR (400 MHz, $CDCl_3$ + trace pyridine) δ 10.21 (bs, 1H, N***H***), 8.43 (d, *J* = 7.5 Hz, 1H, C C***H***), 7.40 – 7.24 (m, 9H, Dmtr C***H***), 7.01 (d, *J* = 7.5 Hz, 1H, C C***H***), 6.88 (d, *J* = 8.8 Hz, 4H, Dmtr C***H***), 6.09 (d, *J* = 1.7 Hz, 1H, 1′-C***H***), 5.11 (ddd, *J* = 9.9, 8.0, 4.9 Hz, 1H, 3′-C***H***), 4.39 (bd, *J* = 7.8 Hz, 1H, 4′-C***H***), 4.35 – 4.22 (m, 2H, POC***H₂***CH2CN), 4.14 – 4.06 (m, 1H, POCH***H***CH2CN), 4.04 (dd, *J* = 4.8, 1.7, Hz, 1H, 2′-C***H***), 3.93 – 3.85 (m, 1H, POCH***H***CH_2CN), 3.82 (s, 6H, Dmtr OC***H***$_3$), 3.73 (dd, *J* = 11.2, 2.1, Hz, 1H, 5′-CH***H***), 3.65 (s, 3H, 2′-OC***H***$_3$), 3.48 (dd, *J* = 11.2, 2.1, Hz, 1H, 5′-C***H***), 2.76 (t, *J* = 6.2 Hz, 2H, C***H***$_2$CN), 2.65 – 2.55 (m, 2H, C***H***$_2$CN), 2.25 (s, 3H, Ac C***H***$_3$).

^{13}C NMR (101 MHz, $CDCl_3$ + trace pyridine) δ = ^{13}C NMR (101 MHz, $CDCl_3$) δ 170.82 (Ac ***C***=O), 163.15 (C ***C***), 158.84 (2C, Dmtr ***C***), 154.84 (C ***C***), 144.42 (C ***C***H), 143.70 (Dmtr ***C***), 134.87 (Dmtr ***C***), 134.74 (Dmtr ***C***), 130.36 (4C, Dmtr ***C***H), 128.44 (2C, Dmtr ***C***H), 128.12 (2C, Dmtr ***C***H), 127.43 (Dmtr ***C***H), 116.39 (***C***N), 116.31 (***C***N), 113.36 (4C, Dmtr ***C***H), 97.14 (C ***C***H), 88.44 (1′-***C***H), 87.36 (Dmtr ***C***), 82.32 (Ri ***C***H), 80.69 (d, *J* = 9.4 Hz, Ri ***C***H), 73.39 (Ri ***C***H), 62.81 (d, *J* = 4.9 Hz, PO***C***H_2CH2CN), 62.60 (d, *J* = 4.3 Hz, PO***C***H_2CH2CN), 60.24 (5′-***C***H_2), 58.78 (2′-O***C***H_3), 55.32 (2C, Dmtr O***C***H_3), 24.87 (Ac ***C***H_3), 19.40 – 19.22 (m, 2C, ***C***H_2CN).

^{31}P NMR (162 MHz, $CDCl_3$ + trace pyridine) δ 65.62.

The phosphorothioate triester (53 mg, 0.066 mmol) was dissolved in MeCN (10 mL) to which was added Et_3N (5 mL). The following day the solvent was stripped off under reduced pressure to give the *title compound* (50 mg, 89%).

^{1}H NMR (400 MHz, $CDCl_3$) δ 10.11 (bs, 1H, N***H***), 8.43 (d, *J* = 7.4 Hz, 0.5H, C C***H***), 8.38 (d, *J* = 7.5 Hz, 1H, C C***H***), 7.45 – 7.42 (m, 2H, Dmtr C***H***), 7.37 – 7.19 (m, 7H, Dmtr C***H***), 6.94 (d, *J* = 7.5 Hz, 0.5H, C C***H***), 6.93 (d, *J* = 7.5 Hz, 0.5H, C C***H***), 6.87 – 6.84 (m, 4H, Dmtr C***H***), 6.08 (d, *J* = 2.0 Hz, 0.5H, 1′-C***H***), 6.06 (d, *J* = 1.6 Hz, 0.5H, 1′-C***H***), 5.05 (ddd, *J* = 10.3, 8.1, 4.7 Hz, 0.5H, 3′-C***H***), 4.99 ddd, *J* = 10.5, 6.6, 3.8 Hz, 0.5H, 3′-C***H***), 4.45 (bd, *J* = 7.4 Hz, 0.5H, 4′-C***H***), 4.36 (bd, *J* = 8.0 Hz, 0.5H, 4′-C***H***), 4.16 – 4.09 (m, 0.5H, POCH***H***CH_2CN), 4.09 – 4.04 (m, 1H, 2′-C***H***), 4.03 – 3.92 (m, 1.5H, POCH***H***CH_2CN), 3.80 (s, 6H, Dmtr OC***H***$_3$), 3.64 – 3.52 (m, 2H, 5′-C***H₂***), 3.63 (s, 1.5H, 2′-OC***H***$_3$), 3.62 (s, 1.5H, 2′-OC***H***$_3$), 3.05 (q, *J* = 7.3 Hz, 6H, CH_3C***H₂***N^+), 2.77 – 2.69 (m, 0.5H, C***H₂***CN), 2.65 – 2.57 (m, 1H, C***H₂***CN), 2.47 – 2.40 (m, 0.5H, C***H₂***CN), 2.24 (s, 3H, Ac C***H₃***), 1.27 (t, *J* = 7.3 Hz, 9H, C***H₃***CH_2N^+).

^{13}C NMR (101 MHz, $CDCl_3$) δ 170.77 (Ac ***C***=O), 162.84 (C ***C***), 158.65 (2C, Dmtr ***C***), 155.00 (C ***C***), 144.86 (C ***C***H), 144.16 (0.5C, Dmtr ***C***), 144.12 (0.5C, Dmtr ***C***), 135.36 (0.5C, Dmtr ***C***), 135.31 (0.5C, Dmtr ***C***), 135.11 (Dmtr ***C***), 130.40 (4C, Dmtr ***C***H), 128.59 – 128.44 (m, 2C, Dmtr ***C***H), 127.99 (2C, Dmtr ***C***H), 127.17 (0.5C, Dmtr ***C***H), 127.13 (0.5C, Dmtr ***C***H), 117.88 (0.5C, ***C***N), 117.81 (0.5C, ***C***N), 113.25 (4C, Dmtr ***C***H), 96.75 (C ***C***H), 88.53 (1′-***C***H), 87.13 (0.5C, Dmtr ***C***), 87.09 (0.5C, Dmtr ***C***), 83.02 (Ri ***C***H), 81.82 (0.5C, d, *J* = 7.4 Hz, Ri ***C***H), 81.41 (0.5C, d, *J* = 8.0 Hz, Ri ***C***H), 71.50 (0.5C, d, *J* = 5.0 Hz, Ri ***C***H), 71.37 (0.5C, d, *J* = 4.5 Hz, Ri ***C***H), 61.35 (0.5C, 5′-***C***H_2), 61.10 (0.5C, 5′-***C***H_2), 60.57 – 60.47 (m, PO***C***H_2CH2CN), 58.55 (2′-O***C***H_3), 55.27 (2C, Dmtr O***C***H_3), 45.66 (Me***C***H_2N^+), 24.90 (Ac ***C***H_3), 19.72 – 19.53 (m, ***C***H_2CN), 8.59 (***C***$H_3CH_2N^+$).

m/z (ESI−) [**8b**^{C}−Et_3NH]$^-$ = 765.1804, calc. $C_{36}H_{38}N_4O_9PS_2^-$ = 765.1818.

1.5 2′-*O*-Methyl-5′-*O*-(4″,4‴-dimethoxytriphenylmethyl)-4-*N*-acetyl cytidine 3′-*O*-(*N*,*N*-diisopropyl)(2-cyanoethyl)phosphorothioamidate, 9a^C.

See section **1.4** for preparation of the crude material: The appropriate fractions (MeCN-water 7:3) were concentrated under reduced pressure until all MeCN had evaporated. To the aqueous solution was added brine, and the cloudy emulsion was extracted with dichloromethane. The organic layer was dried over Na_2SO_4, and the solvent evaporated *in vacuo*. To the residue (193 mg) were added CH_2Cl_2 (100

mL) and flash silica (20 mL) with a trace of pyridine (10 drops). After gentle swirling to ensure adsorption, the silica was washed with CH_2Cl_2 (100 mL) and the filtrate discarded. The silica was then washed with MeOH-CH_2Cl_2 (1:19 200 mL) and the filtrate evaporated to afford the *title compound* (41 mg, 21%).

R_f(MeOH-CH_2Cl_2 1:9) 0.60.

^{1}H NMR (400 MHz, $CDCl_3$ + trace pyridine) δ 10.05 (bs, 1H, N***H***), 8.48 (d, *J* = 7.5 Hz, 0.5H, C C***H***), 8.47 (d, *J* = 7.5 Hz, 0.5H, C C***H***), 7.46 (dd, *J* = 7.1, 1.4 Hz, 0.5H, Dmtr C***H***), 7.42 (dd, *J* = 8.2, 1.4 Hz, 0.5H, Dmtr C***H***), 7.38 – 7.27 (m, 7H, Dmtr C***H***), 6.98 (d, *J* = 7.5 Hz, 0.5H, C C***H***), 6.93 (d, *J* = 7.5 Hz, 0.5H, C C***H***), 6.90 – 6.86 (m, 4H, Dmtr C***H***), 6.12 (d, *J* = 1.6 Hz, 0.5H, 1′-C***H***), 6.09 (d, *J* = 1.6 Hz, 0.5H, 1′-C***H***), 5.11 – 5.02 (m, 1H, 3′-C***H***), 4.41 (bd, *J* = 8.2 Hz, 0.5H, 4′-C***H***), 4.37 (bd, *J* = 8.0 Hz, 0.5H, 4′-C***H***), 4.31 – 4.22 (m, 0.5C, POCH***H***CH_2CN), 4.07 – 3.97 (m, 0.5C, POCH***H***CH_2CN), 4.04 (dd, *J* = 4.9, 1.3 Hz, 2′-C***H***), 3.99 (dd, *J* = 4.7, 1.2 Hz, 2′-C***H***), 3.86 – 3.80 (m, 2H, 5′-CH***H***), 3.84 (s, 3H, Dmtr OC***H***$_3$), 3.83 (s, 3H, Dmtr OC***H***$_3$), 3.76 – 3.38 (m, 2H, NC***H***Me_2), 3.66 (s, 1.5H, 2′-OC***H***$_3$), 3.65 (s, 1.5H, 2′-OC***H***$_3$), 3.54 (dd, *J* = 11.2, 2.4 Hz, 0.5H, 5′-CH***H***), 3.46 (dd, *J* = 11.1, 2.5 Hz, 0.5H, 5′-CH***H***), 2.84 – 2.68 (m, 1H, C***H***$_2$CN), 2.51 – 2.44 (m, 0.5H, C***H***$_2$CN), 2.34 – 2.26 (m, 0.5H, C***H***$_2$CN), 2.26 (s, 1.5H, Ac C***H***$_3$), 2.25 (s, 1.5H, Ac C***H***$_3$), 1.29 (s, 1.5H, NCHMeC***H***$_3$), 1.27 (s, 3H, NCHMeC***H***$_3$), 1.26 (s, 3H, NCHMeC***H***$_3$), 1.24 (s, 1.5H, NCHMeC***H***$_3$), 1.19 (s, 1.5H, NCHMeC***H***$_3$), 1.18 (s, 1.5H, NCHMeC***H***$_3$).

^{13}C NMR (101 MHz, $CDCl_3$) δ 170.60 (Ac ***C***=O), 162.85 (C ***C***), 158.81 (Dmtr ***C***), 158.72 (Dmtr ***C***), 154.93 (C ***C***), 144.79 (0.5C, C ***C***H), 144.63 (0.5C, C ***C***H), 143.92 (0.5C, Dmtr ***C***), 143.84 (0.5C, Dmtr ***C***), 135.15 (0.5C, Dmtr ***C***), 134.98 (0.5C, Dmtr ***C***), 134.91 (Dmtr ***C***), 130.38 (4C, Dmtr ***C***H), 128.56 (Dmtr ***C***H), 128.49 (Dmtr ***C***H), 128.05 (Dmtr ***C***H), 128.01 (Dmtr ***C***H), 127.41 (0.5C, Dmtr ***C***H), 127.22 (0.5C, Dmtr ***C***H), 116.92 (0.5C, ***C***N), 116.82 (0.5C, ***C***N), 113.28 (4C, Dmtr ***C***H), 96.89 (C ***C***H), 88.73 (0.5C, 1′-***C***H), 88.56 (0.5C, 1′-***C***H), 87.32 (0.5C, Dmtr ***C***), 87.22 (0.5C, Dmtr ***C***), 82.99 (0.5C, Ri ***C***H), 82.44 (0.5C, Ri ***C***H), 81.26 (0.5C, d, *J* = 9.7 Hz, Ri ***C***H), 80.98 (0.5C, d, *J* = 8.2 Hz, Ri ***C***H), 71.73 (0.5C, d, *J* = 5.1 Hz, Ri ***C***H), 71.34 (0.5C, d, *J* = 5.5 Hz, Ri ***C***H), 60.80 – 60.54 (2C, m, 5′-***C***H_2 + PO***C***H_2CH_2CN), 58.64 (0.5C, 2′-O***C***H_3), 58.42 (0.5C, 2′-O***C***H_3), 55.31 (Dmtr O***C***H_3), 55.25 (Dmtr O***C***H_3), 47.42 (N***C***HMe_2), 47.37 (N***C***HMe_2), 24.90 (Ac ***C***H_3), 22.69 (NCHMe***C***H_3), 22.53 (NCHMe***C***H_3), 22.41 (NCHMe***C***H_3), 22.31 (NCHMe***C***H_3), 19.53 (0.5C, d, *J* = 9.1 Hz, ***C***H_2CN), 19.17 (0.5C, d, *J* = 9.3 Hz, ***C***H_2CN).

m/z (ESI+) [**9a^{C}**+Na]$^+$ = 856.3111, calc. $C_{42}H_{52}N_5NaO_9PS^+$ = 856.3116.

1.6 2′-*O*-Methyl-5′-*O*-(4″,4‴-dimethoxytriphenylmethyl)-4-*N*-acetyl cytidine 3′-*O*-(*N*,*N*-diisopropyl)(2-cyanoethyl)phosphoramidate, 9b^{C}.

2′-*O*-Methyl-5′-*O*-(4″,4‴-dimethoxytriphenylmethyl)-4-*N*-acetyl cytidine 3′-*O*-(2-cyanoethyl)-*N*,*N*-diisopropyl phosphoramidite (**6^{C}**, 202 mg, 0.25 mmol) was dissolved in MeCN (2 mL) to which was added *ca.* 5M *tert*-butyl hydrogen peroxide (80 μL, 0.5 mmol, 0.5 eq.). After 45 min, the reaction was partitioned between CH_2Cl_2 and 25% $NaHCO_3$, washing the aqueous phase with further CH_2Cl_2. The combined organic layers were dried over Na_2SO_4 and fractionated through a pad of flash silica (20 mL): a trace of pyridine (10 drops) was added to the organic layer which was loaded onto the silica. This was first washed with MeOH-DCM (100 mL, 1:99), discarding the filtrate, then with MeOH-DCM (100 mL, 1:19) and the filtrate evaporated to afford the *title compound* (208 mg, 100%).

R_f(MeOH-$CHCl_3$ 1: 9) 0.52.

^{1}H NMR (400 MHz, $CDCl_3$ + trace d_5-pyridine) δ 10.63 (bs, 0.4H, N***H***), 10.43 (bs, 0.6H, N***H***), 8.42 (d, *J* = 7.5 Hz, 0.4H, C C***H***), 8.41 (d, *J* = 7.5 Hz, 0.6H, C C***H***), 7.39 – 7.35 (m, 2H, Dmtr C***H***), 7.32 – 7.25 (m, 7H, Dmtr C***H***), 7.03 (d, *J* = 7.5 Hz, 0.6H, C C***H***), 6.95 (d, *J* = 7.5 Hz, 0.4H, C C***H***), 6.85 – 6.82 (m, 4H, Dmtr C***H***), 6.04 (bs, 1H, 1′-C***H***), 4.93 – 4.85 (m, 1H, 3′-C***H***), 4.384 – 4.25 (m, 1.8H, , 4′-C***H*** + 0.4×POCH***H***CH_2CN + 0.4×2′-C***H***), 4.14 – 4.08 (m, 0.4H, POCH***H***CH_2CN), 4.06 (d, *J* = 4.3 Hz, 0.6H, 2′-C***H***), 3.96 – 3.81 (m, 1.2H, POCH***H***CH_2CN), 3.79 (s, 2.4H, Dmtr OC***H***$_3$), 3.77 (s, 3.6H, Dmtr OC***H***$_3$), 3.73 – 3.69 (m, 2H, 0.8×5′-CH***H*** + 0.4×2′-OC***H***$_3$), 3.63 (s, 1.8H, 2′-OC***H***$_3$), 3.47 – 3.32 (m, 3.2H, 1.2×5′-CH***H*** + 2×NC***H***Me_2), 2.96 (ddd, *J* = 16.9, 8.1, 5.8 Hz, 0.4H, C***H***$_2$CN), 2.69 (dt,

J = 17.0, 5.1 Hz, 0.4H, C***H₂***CN), 2.49 (dt, *J* = 16.9, 5.9 Hz, 0.6H, C***H₂***CN), 2.38 (dt, *J* = 17.0, 6.4 Hz, 0.6H, C***H₂***CN), 2.21 (s, 1.8H, Ac C***H₃***), 2.15 (s, 1.2H, Ac C***H₃***), 1.20 – 1.16 (m, 9.6H, NCHMeC***H₃***), 1.01 (d, *J* = 6.7 Hz, 2.4H, NCHMeC***H₃***).

^{13}C NMR (101 MHz, $CDCl_3$) δ 170.96 (0.4C, Ac ***C***=O), 170.82 (0.6C, Ac ***C***=O), 162.98 (C ***C***), 158.75 (2C, Dmtr ***C***), 154.91 (0.6C, C ***C***), 154.70 (0.4C, C ***C***), 144.19 (0.6C, C ***C***H), 144.09 (0.4C, C ***C***H), 144.05 (0.6C, Dmtr ***C***), 143.89 (0.4C, Dmtr ***C***), 135.12 (0.6C Dmtr ***C***), 135.08 (0.4C, Dmtr ***C***), 135.00 (0.6C, Dmtr ***C***), 134.98 (0.4C, Dmtr ***C***), 130.26 – 130.20 (m, 4C, Dmtr ***C***H), 128.39 (0.8C, Dmtr ***C***H), 128.28 (1.2C, Dmtr ***C***H), 128.02 (2C, Dmtr ***C***H), 127.27 (0.6C, Dmtr ***C***H), 127.23 (0.4C, Dmtr ***C***H), 117.29 (0.4C, ***C***N), 116.77 (0.6C, ***C***N), 113.28 (4C, Dmtr ***C***H), 96.83 (0.6C, C ***C***H), 96.45 (0.4C, C ***C***H), 88.98 (0.4C, 1′-***C***H), 88.85 (0.6C, 1′-***C***H), 87.33 (0.4C, Dmtr ***C***), 87.02 (0.6C, Dmtr ***C***), 82.75 (0.4C, Ri ***C***H), 82.70 (0.6C, Ri ***C***H), 81.02 – 80.78 (m, Ri ***C***H), 70.97 (0.6C, d, *J* = 5.8 Hz), 70.78 (0.4C, d, *J* = 5.2 Hz), 60.69 – 60.59 (m, 1.4C, 0.4×5′-***C***H_2 + PO***C***H_2CH_2CN), 60.23 (0.6C, d, *J* = 4.7 Hz, 5′-***C***H_2), 58.60 (0.6C, 2′-O***C***H_3), 58.56 (0.4C, 2′-O***C***H_3), 55.21 (2C, Dmtr O***C***H_3), 46.34 (0.6, d, *J* = 4.7 Hz, N***C***HMe$_2$), 46.17 (0.4C, d, *J* = 4.8 Hz, N***C***HMe$_2$), 24.82(0.6C, Ac ***C***H_3), 24.76 (0.4C, Ac ***C***H_3), 22.65 (1.8C, NCHMe***C***H_3), 22.49 (1.2C, NCHMe***C***H_3), 22.27 (1.2C, NCHMe***C***H_3), 22.07 (1.8C, NCHMe***C***H_3), 19.51 – 19.36 (m, ***C***H_2CN).

^{31}P NMR (162 MHz, $CDCl_3$ + trace d_5-pyridine) δ 7.81 (0.38P), 7.45 (0.6P).

m/z (ESI+) [**9a**^{C}+H]$^+$ = 818.3521, calc. $C_{42}H_{53}N_5O_{10}P^+$ = 818.3525.

2. Synthetic Procedures – Purifications by Organic Solvent Nanofiltration

2.1 Organic Solvent Nanofiltration Apparatus

The OSN rig (see Figure S2.1) comprised a pressurized feed tank and four cross-flow membrane cells, each with 51 cm^2 surface area, coupled in series. The retentate was circulated around the system (volume *ca.* 400 mL) using a gear pump (*Michael Smith Ltd.*, UK, GL Series). The apparatus was pressurized with nitrogen gas to 10 bar. The volume of liquid that permeated was balanced by an equal volume of fresh solvent injected by an HPLC pump (*Gilson*, UK, Model 305), using graduated feed and permeate bottles.

Prior to the preparative diafiltration, the membranes were first characterized for rejection performance using a mixture of linear PEGs (MW 400, 2000, and 8000 Da), 1 $g.L^{-1}$ of each dissolved in MeCN. The obtained rejection values were the same as the data reported by Valtcheva *et al.* (*J. Membr. Sci.*, **2014**, *457*, 62-72).

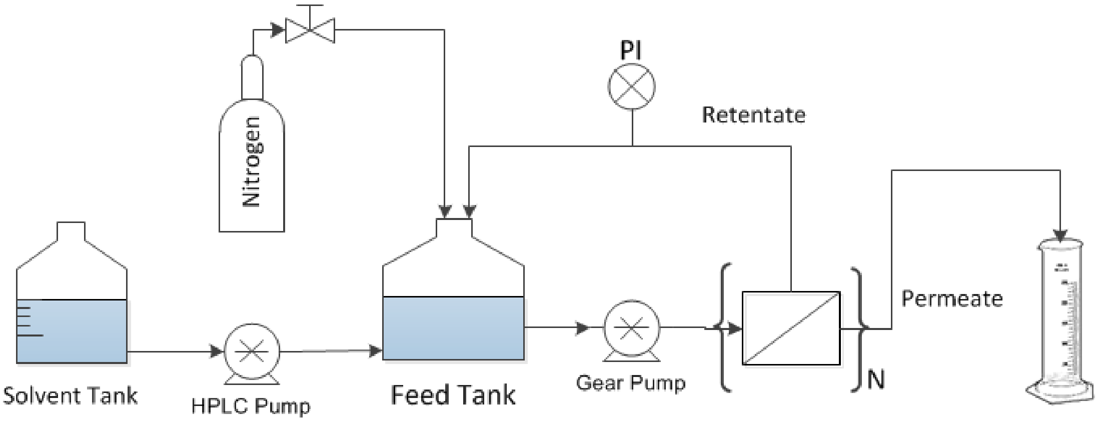

Figure S2.1. Schematic of OSN rig.

2.2 Tris(mUp^{Cne}SmC^{Ac}-OH) homostar, 10.

Tris(2′-methyluridine) homostar **4** (1.130 g, 0.503 mmol) was evaporated from MeCN (3 × 20 mL) *in vacuo*. To the residue was added 2′-*O*-methyl-5′-*O*-(4″,4‴-dimethoxytriphenylmethyl)-4-*N*-acetylcytidine 3′-*O*-(2-cyanoethyl)-*N*,*N*-diisopropyl phosphoramidite (**6**^{C}, 1.808 g, 2.26 mmol, 4.5 eq.) followed by 0.25M ETT in MeCN (18.1 mL, 4.53 mmol, 9 eq.). After 90 min, PADS (1.360 g, 4.50 mmol, 9 eq.) and pyridine (18.1 mL) were added, and after a further 30 min the reaction was diluted into MeCN and poured into the OSN rig. Once 12 diavolumes had permeated, the retentate was evaporated to give crude 5′-Dmtr homostar **7** (2.267 g) as a cream-coloured glass.

Partially purified tris(DmtrO-2-mer) homostar **7** (2.267 g) was placed in dichloromethane (62 mL), to which were added pyrrole (1.05 mL) then DCA (0.62 mL). After 35 min the reaction was complete by TLC and pyridine (2 mL) was added. The solution was diluted with MeCN (100 mL) and concentrated until all the dichloromethane had evaporated. This solution was then diluted with further MeCN containing DCA (1.0 vol%) and 5 diavolumes were permeated, followed by 10 diavolumes neat MeCN. The retentate was evaporated to dryness, and the residual glass was re-dissolved in dichloromethane (15 mL). The solution was added dropwise to briskly stirred diethyl ether (250 mL), and the precipitate collected to afford tris(HO-2-mer) homostar **10** (1.240 g, 70%) as a grey-green powder.

2.3 ris(mUp^{Cne}SmC^{Ac}p^{Cne}SmC^{Ac}-OH) homostar, 14.

Tris(HO-2-mer) homostar **10** (1.240 g, 0.351 mmol) was evaporated from MeCN (3 × 20 mL) *in vacuo*. To the residue was added 2′-*O*-methyl-5′-*O*-(4″,4‴-dimethoxytriphenylmethyl)-4-*N*-

acetylcytidine 3′-*O*-(2-cyanoethyl)-*N*,*N*-diisopropyl phosphoramidite (**6**^{C}, 1.267 g, 1.58 mmol, 4.5 eq.) followed by 0.25M ETT in MeCN (12.6 mL, 3.16 mmol, 9 eq.). After 40 min the reaction appeared incomplete by HPLC, so further phosphoramidite **6**^{C} (0.336 g, 0.42 mmol, 1.2 eq.) and ETT (3.5 mL, 0.89 mmol, 2.5 eq.) were added, but after another 20 min the HPLC had changed little. Consequently, PADS (1.206 g, 3.99 mmol, 11.4 eq.) and pyridine (16.1 mL) were added, and after a further 35 min the reaction was diluted into MeCN and poured into the OSN rig. Once 12 diavolumes had permeated, the retentate was evaporated to give crude 5′-Dmtr homostar **13** (2.678 g) as a brown glass.

Partially purified tris(DmtrO-3-mer) homostar **13** (2.596 g) was placed in dichloromethane (45 mL), to which were added pyrrole (0.76 mL) then DCA (0.35 mL). After 25 min the reaction was incomplete by TLC, so further DCA (0.23 mL) was added, and after another 20 min the reaction was complete. The solution was diluted with MeCN (100 mL) and concentrated until all the dichloromethane had evaporated. This solution was then diluted with further MeCN containing DCA (0.1 vol%) and 5 diavolumes were permeated, followed by 10 diavolumes neat MeCN. The retentate was evaporated to dryness, and the residual glass (1.478 g) was re-dissolved in dichloromethane-MeCN (40 mL, 1:1 v/v). The solution was added dropwise to briskly stirred diethyl ether (400 mL), and the precipitate collected to afford tris(HO-3-mer) homostar **14** (1.441 g, 88%) as a pale brown powder.

2.4 Tris(mUp^{Cne}SmC^{Ac}p^{Cne}SmC^{Ac}p^{Cne}SmA^{Bz}-OH) homostar, 16.

Tris(HO-3-mer) homostar **14** (1.353 g, 0.280 mmol) was dissolved in DMF (2 mL) and the solution was co-evaporated from MeCN (3 × 20 mL) *in vacuo*. To the residue was added 2′-*O*-methyl-5′-*O*-(4″,4‴-dimethoxytriphenylmethyl)-7-*N*-benzoyladenosine 3′-*O*-(2-cyanoethyl)-*N*,*N*-diisopropyl phosphoramidite (**6**^{A}, 1.117 g, 1.26 mmol, 4.5 eq.) followed by 0.25M ETT in MeCN (10.1 mL, 2.52 mmol, 9 eq.). After 35 min a solution of PADS (756 mg, 2.52 mmol, 9 eq.) in pyridine (10.1 mL) was added, and after a further 35 min the reaction was diluted into MeCN and poured into the OSN rig. Once 12 diavolumes had permeated, the retentate was evaporated to give crude 5′-Dmtr homostar **15** (2.477 g) as a brown glass.

Partially purified tris(DmtrO-4-mer) homostar **15** (2.422 g) was placed in dichloromethane (36 mL), to which was added pyrrole (0.60 mL) then DCA (0.36 mL). After 35 min the reaction was complete by TLC and pyridine (0.40 mL) was added. The solution was diluted with MeCN (100 mL), and concentrated until all the dichloromethane had evaporated. This solution was then diluted with further MeOH-MeCN (1:9 v/v) containing pyridinium.DCA (0.5 vol%) and 5 diavolumes were permeated, followed by 10 diavolumes MeOH-MeCN (1:9 v/v). The retentate was evaporated to dryness, and the residual glass (1.556 g) was re-dissolved in dichloromethane-MeOH (25 mL, 9:1 v/v). The solution was added dropwise to briskly stirred diethyl ether (300 mL), and the precipitate collected to afford tris(HO-4-mer) homostar **16** (1.478 g, 84.6%) as a pale brown powder.

2.5 Tris(mUp^{Cne}SmC^{Ac}p^{Cne}SmC^{Ac}p^{Cne}SmA^{Bz}p^{Cne}SmU-OH) homostar, 18.

Tris(HO-4-mer) homostar **16** (1.418 g, 0.222 mmol) was dissolved in DMF (4 mL) to which was added MeCN (20 mL) and the solution evaporated *in vacuo*; this was repeated with DMF (2 mL) plus MeCN (20 mL), and finally with neat MeCN (15 mL). To the residue was added 2′-*O*-methyl-5′-*O*-(4″,4‴-dimethoxytriphenylmethyl)uridine 3′-*O*-(2-cyanoethyl)-*N*,*N*-diisopropyl phosphoramidite (**6**^{U}, 764 mg, 1.00 mmol, 4.5 eq.) followed by 0.25M ETT in MeCN (8.0 mL, 2.00 mmol, 9 eq.). After 35 min PADS (605 mg, 2.00 mmol, 9 eq.) and pyridine (8.0 mL) were added, and after a further 40 min the reaction was diluted into MeOH-MeCN (1:9 v/v) and poured into the OSN rig. Once 12 diavolumes had permeated, the retentate was evaporated to give crude 5′-Dmtr homostar **17** (1.961 g) as a brown glass.

Partially purified tris(DmtrO-5-mer) homostar **17** (1.896 g) was placed in dichloromethane (28 mL), to which was added pyrrole (0.48 mL) then DCA (0.28 mL). After 25 min the reaction was slightly cloudy and TLC showed incomplete reaction, so further DCA (0.14 mL) was added when all

the solids dissolved. After a further 25 min the reaction was complete by TLC and pyridine (0.82 mL) was added when a thick precipitate formed. The mixture was diluted with MeCN (100 mL), the liquid concentrated until all the dichloromethane had evaporated, then MeOH (5 mL) was added when all the solids dissolved. The solution was diluted with further MeOH-MeCN (1:9 v/v) containing pyridinium.DCA (0.5 vol%) and 5 diavolumes were permeated, followed by 10 diavolumes MeOH-MeCN (1:9 v/v). The retentate was evaporated to dryness, and the residual glass was re-dissolved in dichloromethane-MeOH (25 mL, 4:1 v/v). The solution was added dropwise to briskly stirred diethyl ether (300 mL), and the precipitate collected to afford tris(HO-5-mer) homostar **19** (1.524 g, 94%) as a brown powder.

2.6 HO-mUpSmCpSmCpSmApSmU-OH, 19.

Tris(HO-5-mer) homostar **21** (152 mg, 20.2 μmol) was suspended in MeCN (4 mL), when the glass turned to gummy globules but did not dissolve. Upon addition of diethylamine (1 mL) the briskly stirred suspension became cloudy, although the solids did not dissolve. After 30 min the solvent was stripped off and the residue dissolved in conc. ammonia (2 mL). The solution was filtered through a tight plug of cotton wool to remove small amounts of dark brown organically soluble material, and the flask and filter were washed with further aq. ammonia (3 × 1mL). The filtrate was placed in an Ace® pressure tube, fitted with a PTFE screw top and O-ring seal, the assembly was closed and the honey coloured solution heated at 55 °C overnight. The following day the reaction was cooled to room temperature and once more filtered through a plug of cotton wool. The filtrate was co-evaporated from ethanol (×3), and the resultant off-white solid triturated with MeCN to afford the crude *title compound* (132 mg, 74% by HPLC).

2.7 Tris(mUp^{Cne}SmC^{Ac}p^{Cne}SmC^{Ac}p^{Cne}SmA^{Bz}p^{Cne}SmUp^{Cne}SmU-OH) homostar, 21.

Tris(HO-5-mer) homostar **18** (1.286 g, 0.171 mmol) was dissolved in DMF (4 mL) to which was added MeCN (20 mL) and the solution evaporated *in vacuo*; this was repeated with DMF (2 mL) plus MeCN (20 mL), and finally with neat MeCN (20 mL). To the residue was added 2′-*O*-methyl-5′-*O*-(4″,4‴-dimethoxytriphenylmethyl)uridine 3′-*O*-(2-cyanoethyl)-*N*,*N*-diisopropyl phosphoramidite (**6**$^{\mathbf{U}}$, 585 mg, 0.769 mmol, 4.5 eq.) followed by 0.25M ETT in MeCN (6.2 mL, 1.55 mmol, 9 eq.). After 35 min PADS (465 mg, 1.55 mmol, 9 eq.) and pyridine (6.2 mL) were added, and after a further 35 min the reaction was diluted into MeOH-MeCN (3:17 v/v) and poured into the OSN rig. Once 12 diavolumes had permeated, the retentate was evaporated to give crude 5′-Dmtr homostar **20** (1.779 g) as a brown glass.

Partially purified tris(DmtrO-6-mer) homostar **20** (1.708 g) was placed in dichloromethane (28 mL), to which was added pyrrole (0.48 mL) then DCA (0.28 mL). After 45 min the reaction was complete by TLC and pyridine (0.28 mL) was added. The mixture was diluted with MeCN (100 mL) and the liquid concentrated until all the dichloromethane had evaporated. To this solution was then added MeOH (20 mL), the solution was diluted with further MeOH-MeCN (3:17 v/v) containing pyridinium.DCA (0.5 vol%) and 5 diavolumes were permeated. The flux was observed to drop significantly, so this was followed by 10 diavolumes MeOH-MeCN (1:4 v/v) when the flux improved. The retentate was evaporated to dryness, and the residual glass was re-dissolved in dichloromethane-MeOH. The solution was added dropwise to briskly stirred diethyl ether (300 mL), and the precipitate collected to afford tris(HO-6-mer) homostar **21** (1.394 g, 98%) as a brown powder.

2.8 Tris(mUp^{Cne}SmC^{Ac}p^{Cne}SmC^{Ac}p^{Cne}SmA^{Bz}p^{Cne}SmUp^{Cne}SmUp^{Cne}SmC^{Ac}-OH) homostar, 23.

Tris(HO-6-mer) homostar **21** (1.322g, 0.152 mmol) was dissolved in DMF (4 mL) to which was added MeCN (20 mL) and the solution evaporated *in vacuo*; this was repeated with DMF (2 mL) plus MeCN (20 mL), and finally with neat MeCN (20 mL). To the residue was added 2′-*O*-methyl-5′-*O*-(4″,4‴-dimethoxytriphenylmethyl)-4-*N*-acetylcytidine 3′-*O*-(2-cyanoethyl)-*N*,*N*-diisopropyl

phosphoramidite ($\mathbf{6^{C}}$, 549 mg, 0.683 mmol, 4.5 eq.) followed by 0.25M ETT in MeCN (5.5 mL, 1.37 mmol, 9 eq.). After 40 min PADS (414 mg, 1.37 mmol, 9 eq.) and pyridine (5.5 mL) were added, and after a further 40 min the reaction was diluted into MeOH-MeCN (1:4 v/v) and poured into the OSN rig. Once 12 diavolumes had permeated, the retentate was evaporated to give crude 5′-Dmtr homostar **22** (1.670 g) as a brown glass.

Partially purified tris(DmtrO-7-mer) homostar **22** (1.616 g) was placed in dichloromethane (25 mL), to which was added pyrrole (0.43 mL) then DCA (0.25 mL). After 30 min the reaction was not quite complete so further DCA (0.125 mL) was added. After a further 45 min the reaction was complete by TLC and pyridine (0.40 mL) was added. The mixture was diluted with MeCN (100 mL) and the liquid concentrated until all the dichloromethane had evaporated. To this solution was then added MeOH (20 mL), the solution was diluted with further MeOH-MeCN (1:4 v/v) containing pyridinium.DCA (1 vol%) and 5 diavolumes were permeated, followed by 10 diavolumes MeOH-MeCN (1:4 v/v). The retentate was evaporated to dryness, and the residual glass (1.511 g) was re-dissolved in dichloromethane-MeOH (4:1 v/v, 15 mL). The solution was added dropwise to briskly stirred diethyl ether (300 mL), and the precipitate collected to afford tris(HO-7-mer) homostar **23** (1.432 g, 94%) as a pink/brown powder.

2.9 Tris(mUp^{Cne}SmC^{Ac}p^{Cne}SmC^{Ac}p^{Cne}SmA^{Bz}p^{Cne}SmUp^{Cne}SmUp^{Cne}SmC^{Ac}p^{Cne}SmG$^{i\mathrm{Bu}}$-OH) homostar, 25.

Tris(HO-7-mer) homostar **23** (1.335g, 0.133 mmol) was dissolved in DMF (4 mL) to which was added MeCN (20 mL) and the solution evaporated *in vacuo*; this was repeated with DMF (2 mL) plus MeCN (20 mL), and finally with neat MeCN (20 mL). To the residue was added 2′-*O*-methyl-5′-*O*-(4″,4‴-dimethoxytriphenylmethyl)-7-*N*-isobutyryl guanidine 3′-*O*-(2-cyanoethyl)-*N*,*N*-diisopropyl phosphoramidite ($\mathbf{6^{G}}$, 523 mg, 0.600 mmol, 4.5 eq.) followed by 0.25M ETT in MeCN (4.8 mL, 1.2 mmol, 9 eq.). After 40 min PADS (363 mg, 1.2 mmol, 9 eq.) and pyridine (4.8 mL) were added, and after a further 40 min the reaction was diluted into MeOH-MeCN (1:4 v/v) and poured into the OSN rig. Once 12 diavolumes had permeated, the retentate was evaporated to give crude 5′-Dmtr homostar **24** (1.725 g) as a brown glass; should be left under high vacuum to remove all MeOH which inhibits detritylation.

Partially purified tris(DmtrO-8-mer) homostar **24** (1.670 g) was placed in dichloromethane (25 mL), to which was added pyrrole (0.43 mL) then DCA (0.25 mL). After 45 min the reaction was complete by TLC and pyridine (0.25 mL) was added. The mixture was diluted with MeCN (95 mL) and the liquid concentrated until all the dichloromethane had evaporated. To this solution was then added MeOH (15 mL), the solution was diluted with further MeOH-MeCN (1:4 v/v) containing pyridinium.DCA (1 vol%) and 5 diavolumes were permeated, followed by 10 diavolumes MeOH-MeCN (1:4 v/v). The retentate was evaporated to dryness, and the residual glass (1.457 g) was re-dissolved in dichloromethane-MeOH (4:1 v/v, 15 mL). The solution was added dropwise to briskly stirred diethyl ether (300 mL), and the precipitate collected to afford tris(HO-8-mer) homostar **25** (1.377 g, 93%) as a pink/brown powder.

2.10 Tris(mUp^{Cne}SmC^{Ac}p^{Cne}SmC^{Ac}p^{Cne}SmA^{Bz}p^{Cne}SmUp^{Cne}SmUp^{Cne}SmC^{Ac}p^{Cne}SmG$^{i\mathrm{Bu}}$p^{Cne}SmG$^{i\mathrm{Bu}}$-OH) homostar, 27.

Tris(HO-8-mer) homostar **25** (1.314g, 0.114 mmol) was dissolved in DMF (4 mL) to which was added MeCN (20 mL) and the solution evaporated *in vacuo*; this was repeated with DMF (2 mL) plus MeCN (20 mL), and finally with neat MeCN (20 mL). To the residue was added 2′-*O*-methyl-5′-*O*-(4″,4‴-dimethoxytriphenylmethyl)-7-*N*-isobutyryl guanidine 3′-*O*-(2-cyanoethyl)-*N*,*N*-diisopropyl phosphoramidite ($\mathbf{6^{G}}$, 448 mg, 0.514 mmol, 4.5 eq.) followed by 0.25M ETT in MeCN (4.1 mL, 1.03 mmol, 9 eq.); at first the substrate did not dissolve but with gentle swirling a clear solution was obtained after 5 min. After 35 min PADS (312 mg, 1.03 mmol, 9 eq.) and pyridine (4.1 mL) were added, and after a further 35 min the reaction was diluted into MeOH-MeCN (1:4 v/v) and poured into

the OSN rig. Once 12 diavolumes had permeated, the retentate was evaporated to give crude 5′-Dmtr homostar **26** (1.680 g) as a brown glass.

Partially purified tris(DmtrO-9-mer) homostar **26** (1.626 g) was placed in dichloromethane (25 mL), to which was added pyrrole (0.43 mL) then DCA (0.25 mL), but the substrate did not dissolve completely so further DCA (0.125 mL) was added when a clear orange solution was obtained. After 40 min the reaction was complete by TLC and pyridine (0.375 mL) was added, causing a thick precipitate to form. The supernatant was diluted with MeCN (100 mL) and the liquid concentrated until all the dichloromethane had evaporated. To this suspension was then added MeOH (20 mL) when all the brown gum dissolved. The solution was diluted with further MeOH-MeCN (1:4 v/v) containing pyridinium.DCA (1 vol%) and 5 diavolumes were permeated. However, the flux dropped around 4-fold, so the methanol concentration of the next 10 diavolumes was increased to MeOH-MeCN (1:3 v/v), when the flux recovered. The retentate was evaporated to dryness and the residual glass was re-dissolved in dichloromethane-MeOH (4:1 v/v, 15 mL). The solution was added dropwise to briskly stirred diethyl ether (300 mL), and the precipitate collected to afford tris(HO-9-mer) homostar **27** contaminated with incompletely chain extended homostar (1.362 g, *ca*. 94%).

2.11 HO-mUpSmCpSmCpSmApSmUpSmUpSmCpSmGpSmG-OH, 28.

Tris(HO-9-mer) homostar **27** (266 mg, 20.5 μmol) was suspended in MeCN (12 mL), to which was added diethylamine (2.8 mL) and the suspension was stirred briskly. After 30 min the solvent was stripped off and the residue dissolved in conc. ammonia (2 mL). The solution was filtered through a tight plug of cotton wool, and the flask and filter were washed with further aq. ammonia (3 × 1mL). The filtrate was placed in an Ace® pressure tube, fitted with a PTFE screw top and O-ring seal, the assembly was closed and the solution heated at 55 °C overnight. The following day the reaction was cooled to room temperature and once more filtered through a plug of cotton wool. The filtrate was co-evaporated from ethanol (×3), and the resultant off-white solid triturated with MeCN to afford the crude 9-mer (211 mg, 49% **28 +** 17% **29** by HPLC).

The crude precipitate (180 mg) was purified twice by ion exchange on *GE* Source 15Q: First column (13 cm × 2.6 cm diameter) used a gradient of 0.2M NaCl plus 20mM NaOH (buffer A), and 2M NaCl plus 20mM NaOH (buffer B), 0 → 75% buffer B eluted at 40 $mL.min^{-1}$ over 45 min; second column (9 cm × 1.6 cm diameter), 0 → 55% buffer B in buffer A eluted at 25 $mL.min^{-1}$ over 25 min. The purified fractions were desalted through a *GE* 26/10 column eluted at 10 $mL.min^{-1}$, to afford the *title compound* (**28**, 29 mg, 15%) in 94% purity.

3. ^{1}H, ^{13}C & ^{31}P NMR from chromatographically purified materials

3.1 2′-*O*-Methyl-3′-O-succinyl-5′-*O*-(4″,4‴-dimethoxytriphenylmethyl)uridine, triethylammonium salt, 2 (400 MHz, $CDCl_3$).

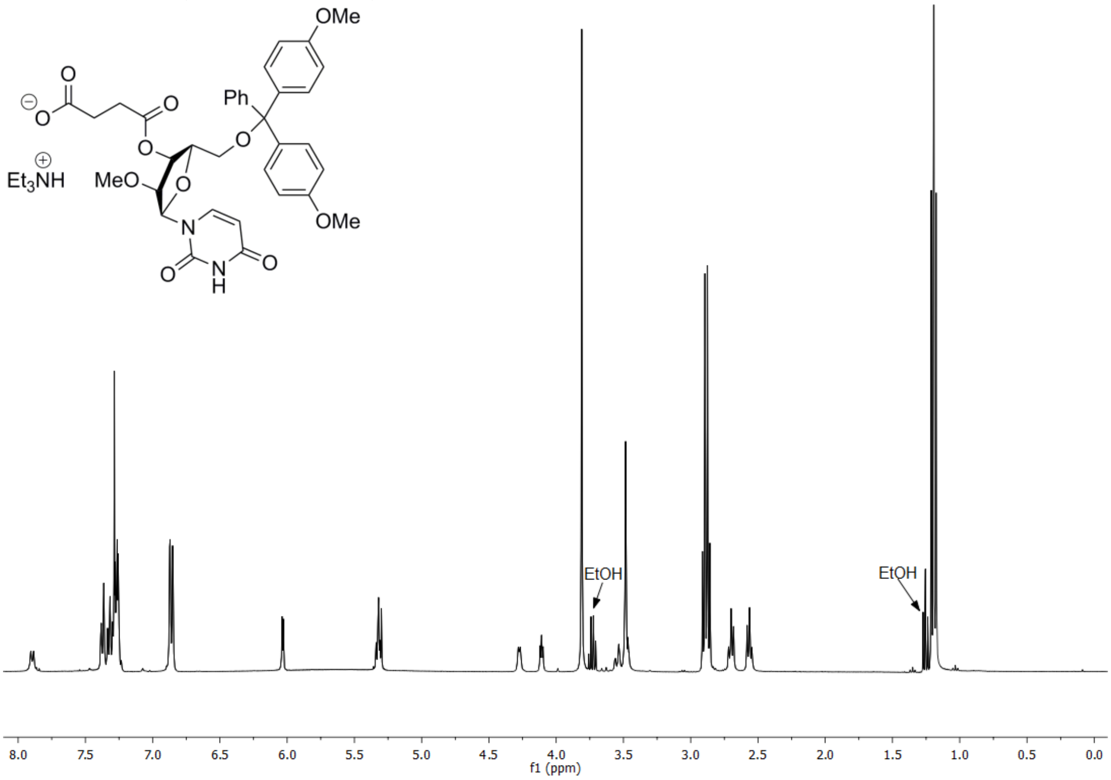

Figure S3.1.1 ^{1}H-NMR spectrum

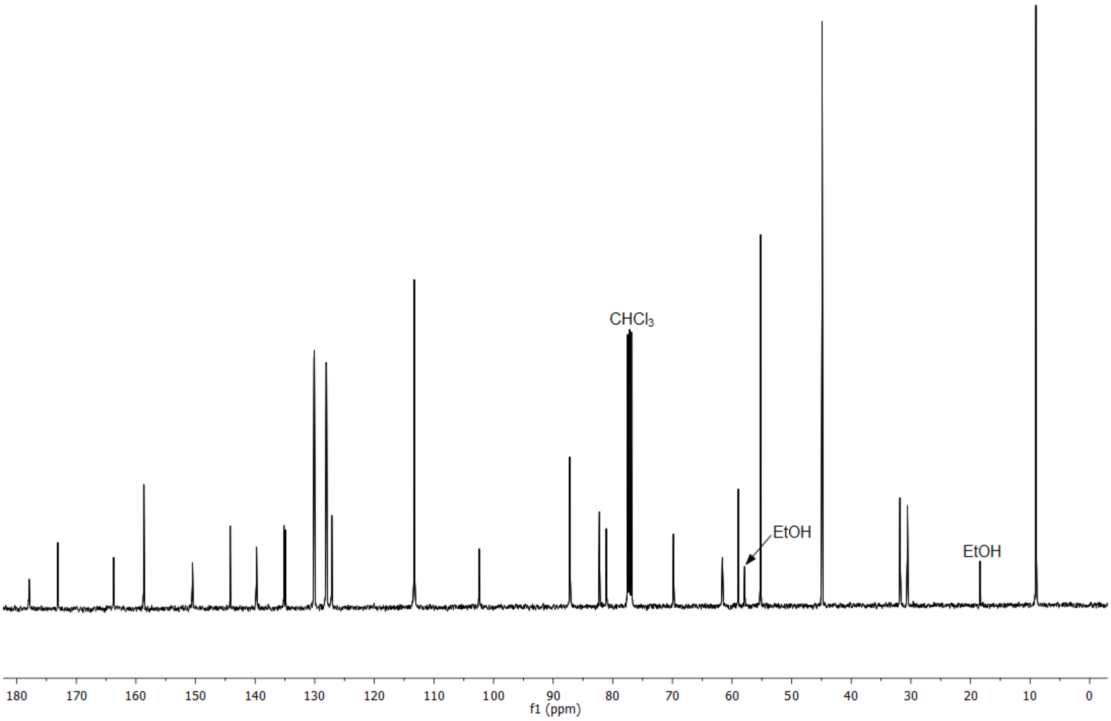

Figure S3.1.2 ^{13}C-NMR spectrum

3.2 Tris-1,3,5-{ω-[2′-*O*-methyl-5′-*O*-(4″,4‴-dimethoxytriphenylmethyl)uridine-3′-*O*-succinyloxy]-octa(ethylene glycol)-α-oxymethyl}benzene, tris(mU-ODmtr) homostar, 3 (400 MHz, $CDCl_3$).

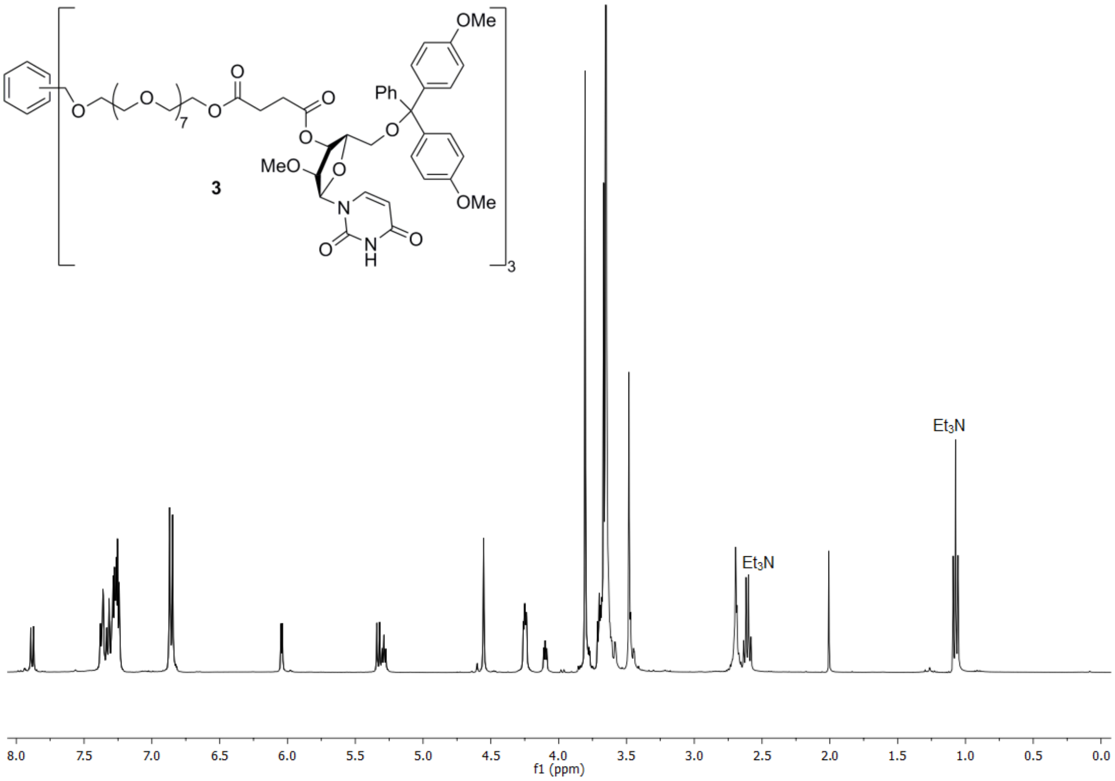

Figure S3.2.1 ^{1}H-NMR spectrum

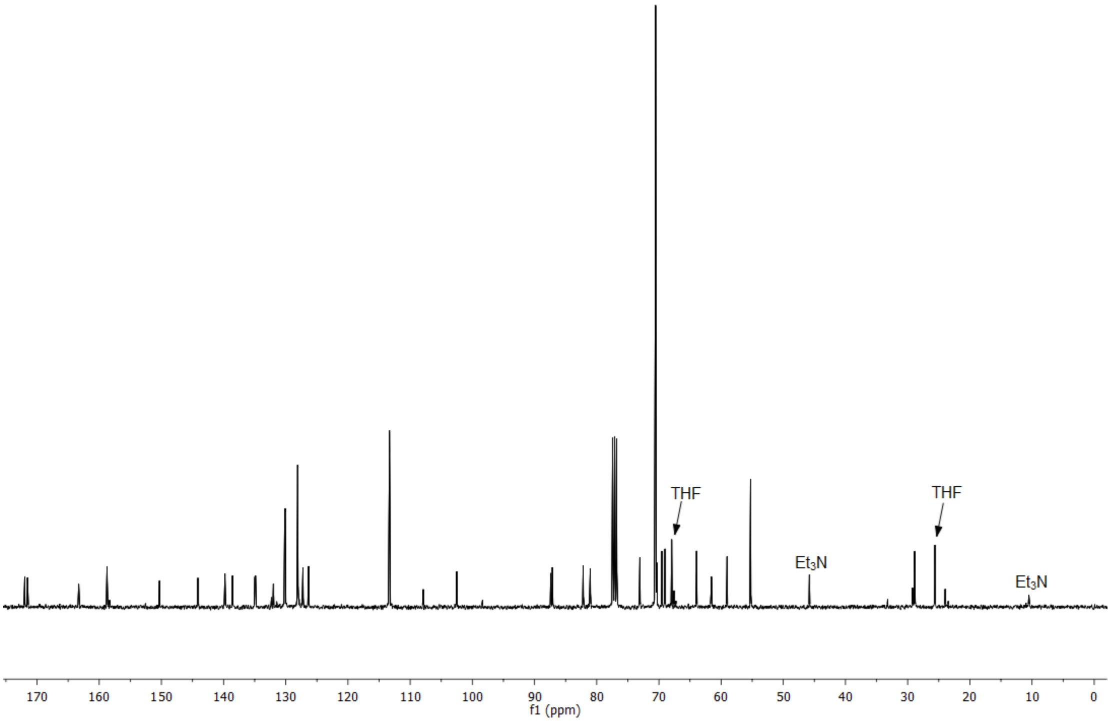

Figure S3.2.2 ^{13}C-NMR spectrum

3.3 Tris-1,3,5-[ω-(2′-*O*-methyluridine-3′-*O*-succinyloxy)octa(ethylene glycol)-α-oxymethyl]benzene, tris(mU-OH) homostar, 4 (400 MHz, $CDCl_3$).

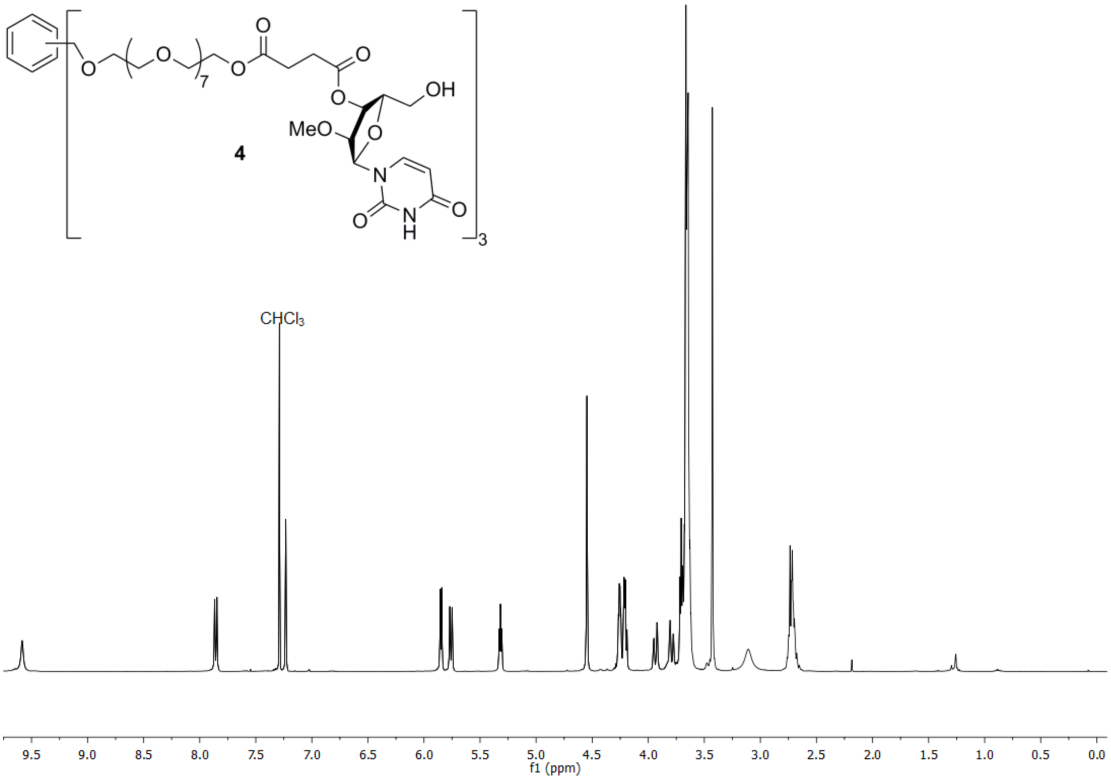

Figure S3.3.1 ^{1}H-NMR spectrum

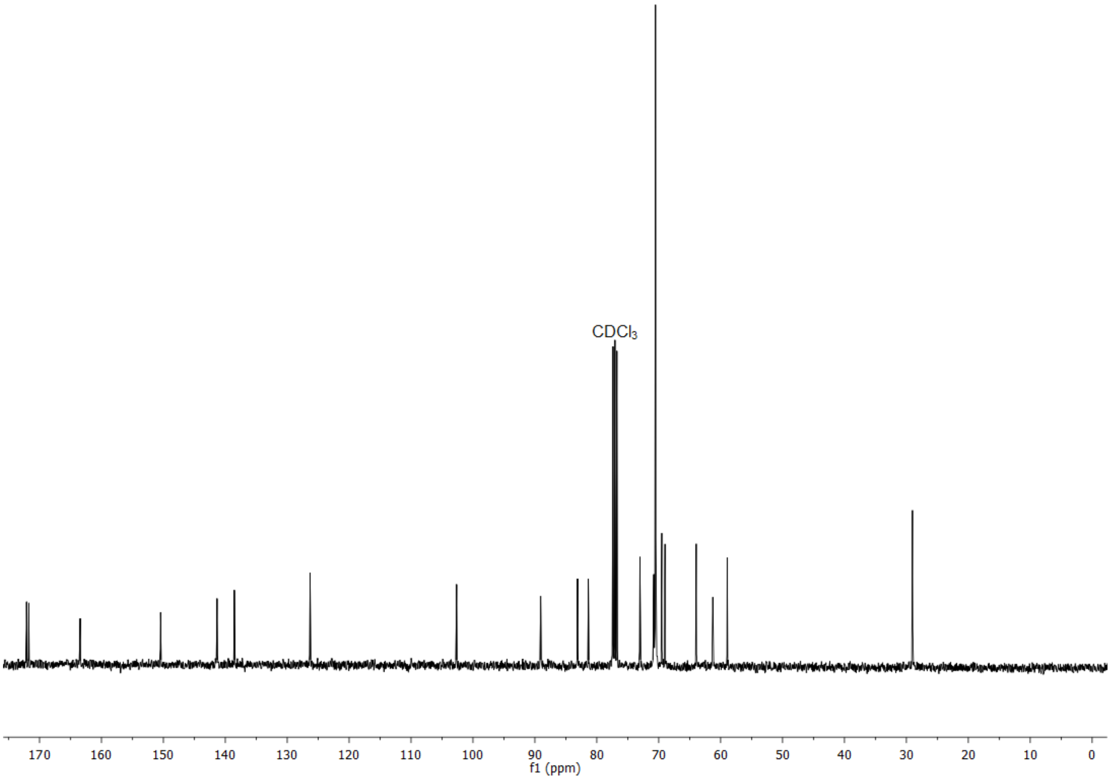

Figure S3.3.2 ^{13}C-NMR spectrum

3.4 Bis-1,3-[ω-(2′-*O*-methyluridine-3′-*O*-succinyloxy)octa(ethylene glycol)-α-oxymethyl]-5-[ω-(2,6-dichlorobenzoylox)octa(ethylene glycol)-α-oxymethyl]benzene, bis(mU-OH)Dcb homostar, 5 (400 MHz, $CDCl_3$).

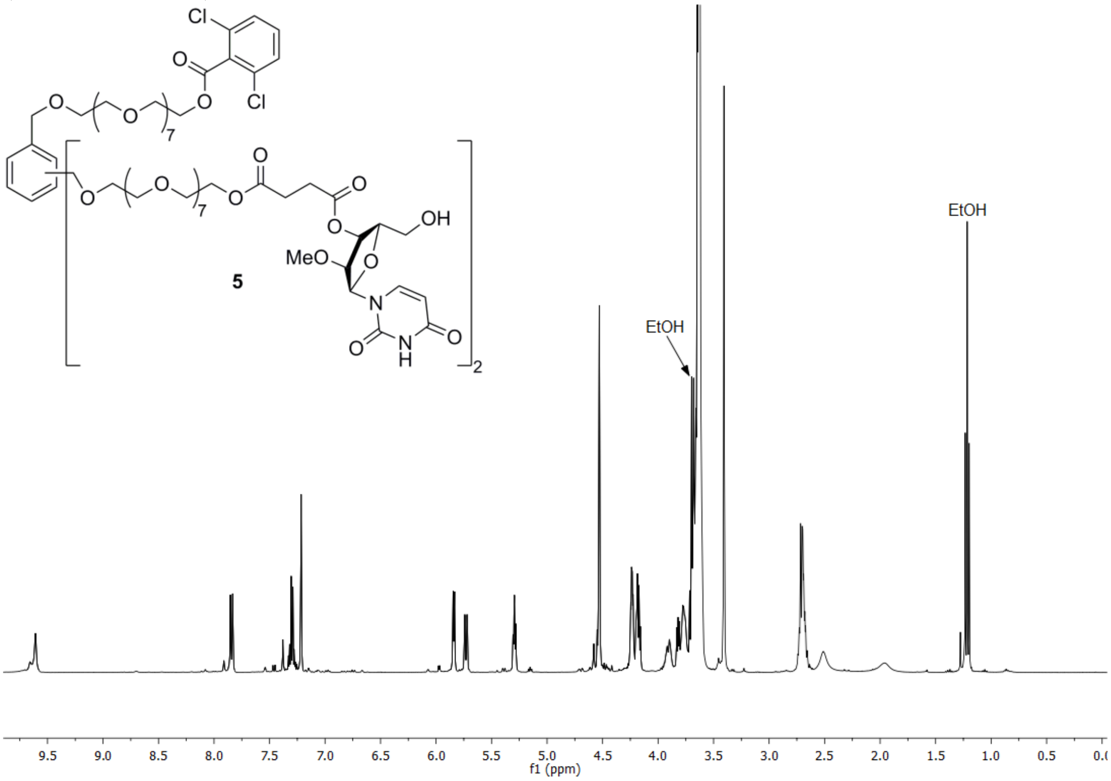

Figure S3.4.1 ^{1}H-NMR spectrum

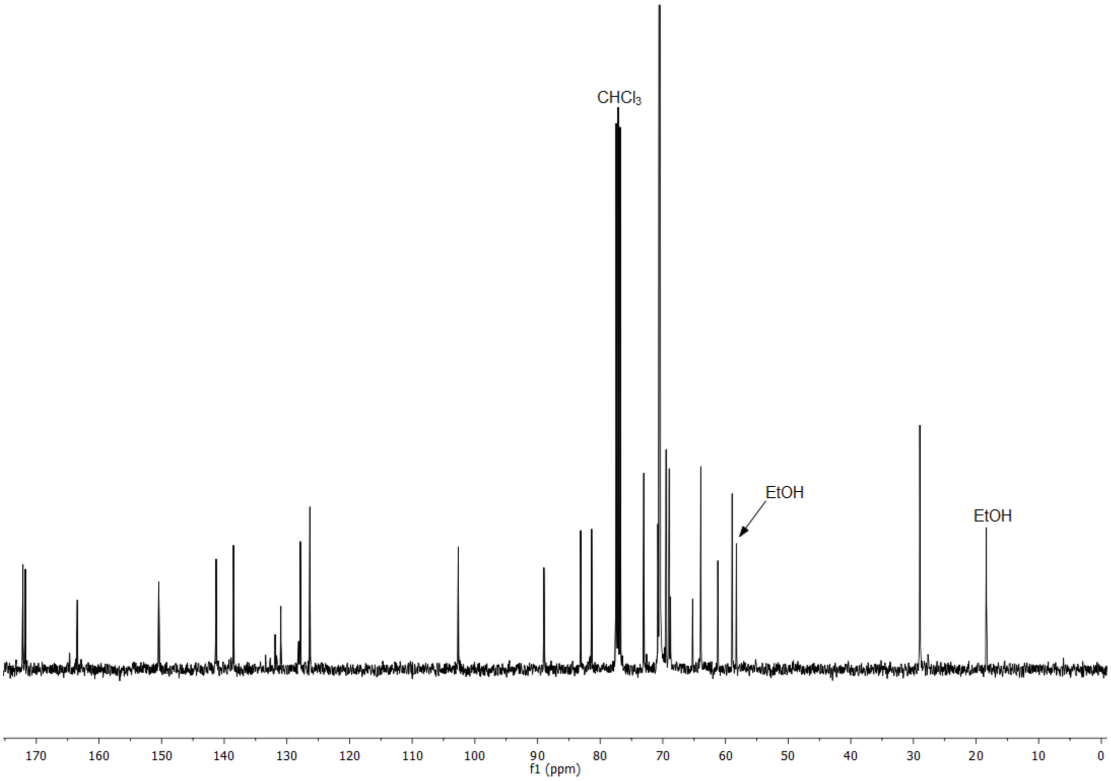

Figure S3.4.2 ^{13}C-NMR spectrum

3.5 Tris(mUp^{Cne}SmC^{Ac}-ODmtr) homostar, 7 (400 MHz, D_6-acetone).

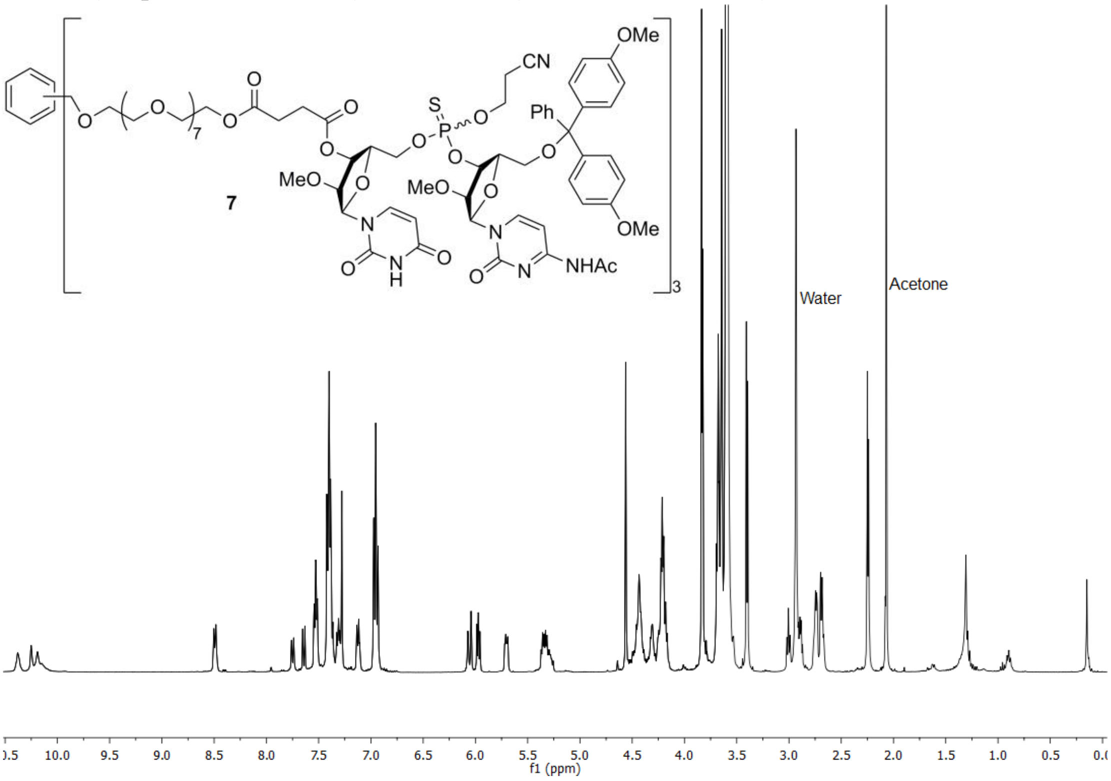

Figure S3.5.1 ^{1}H-NMR spectrum

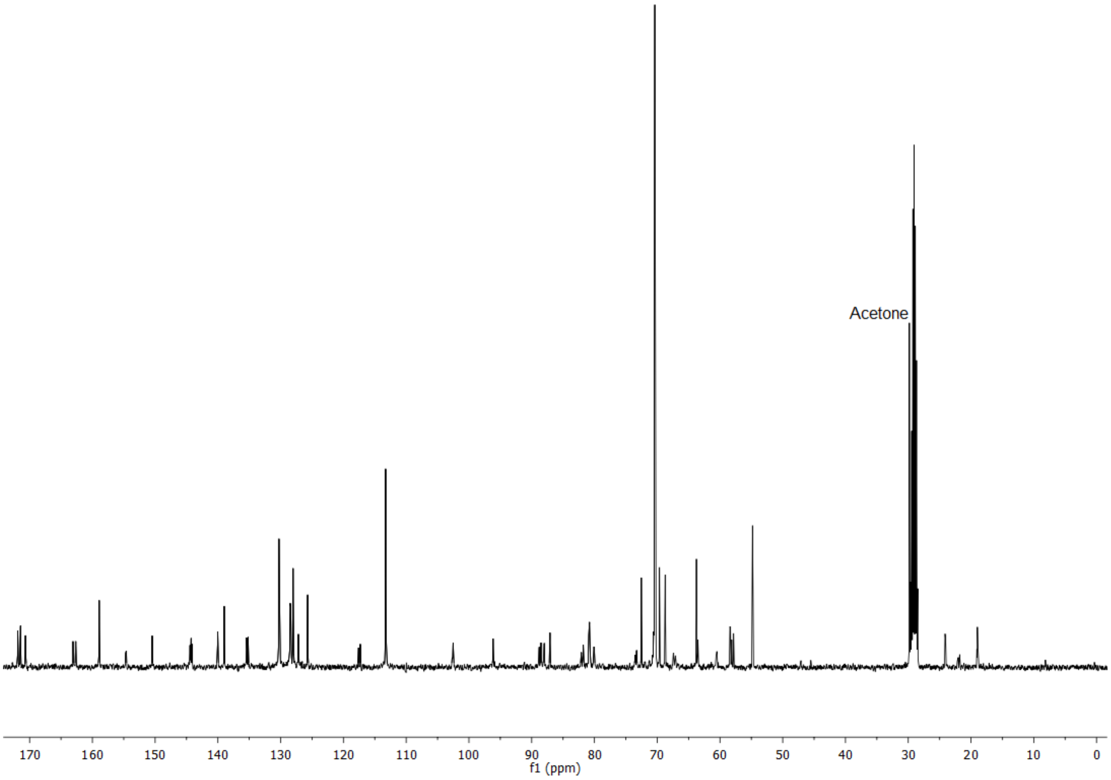

Figure S3.5.2 ^{13}C-NMR spectrum

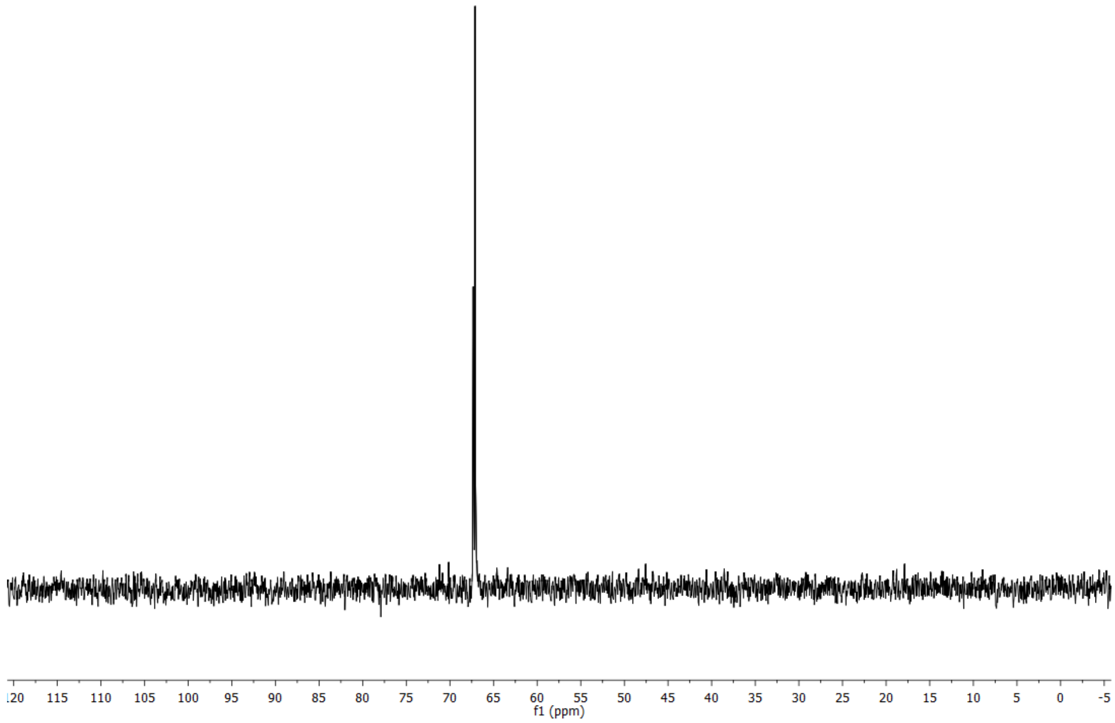

Figure S3.5.3 ^{31}P-NMR spectrum

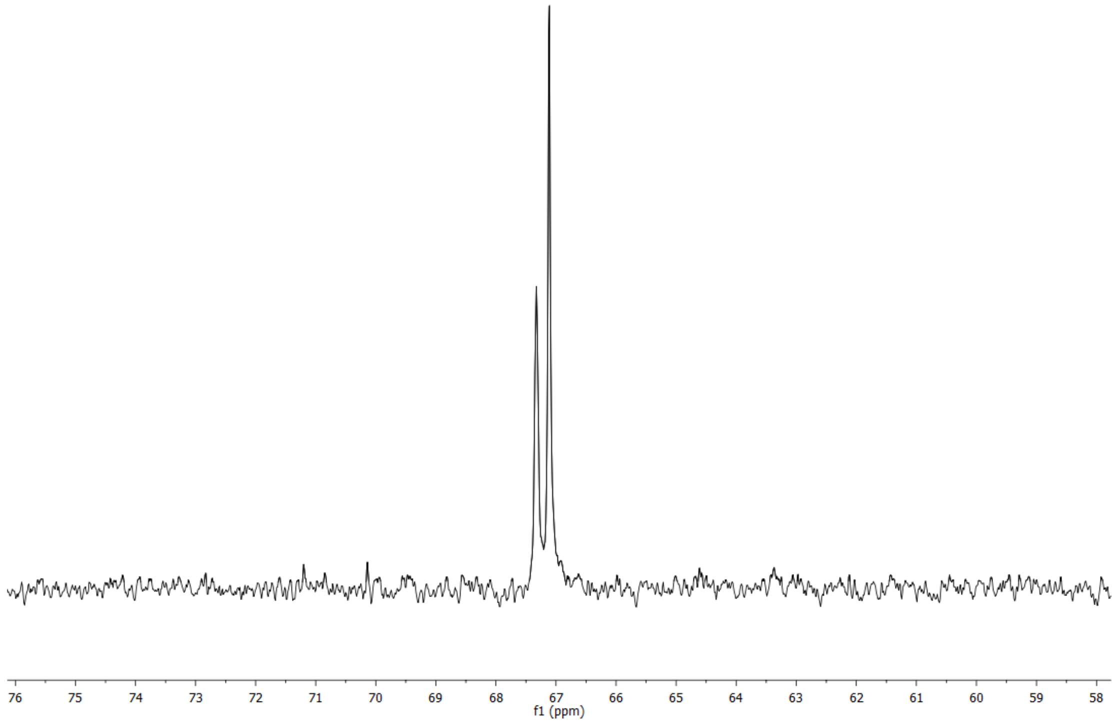

Figure S3.5.4 ^{31}P-NMR spectrum, expansion

3.6 Tris(mUp^{Cne}SmC^{Ac}-OH) homostar, 10 (500 MHz, $CDCl_3$-CD_3OD 2:1).

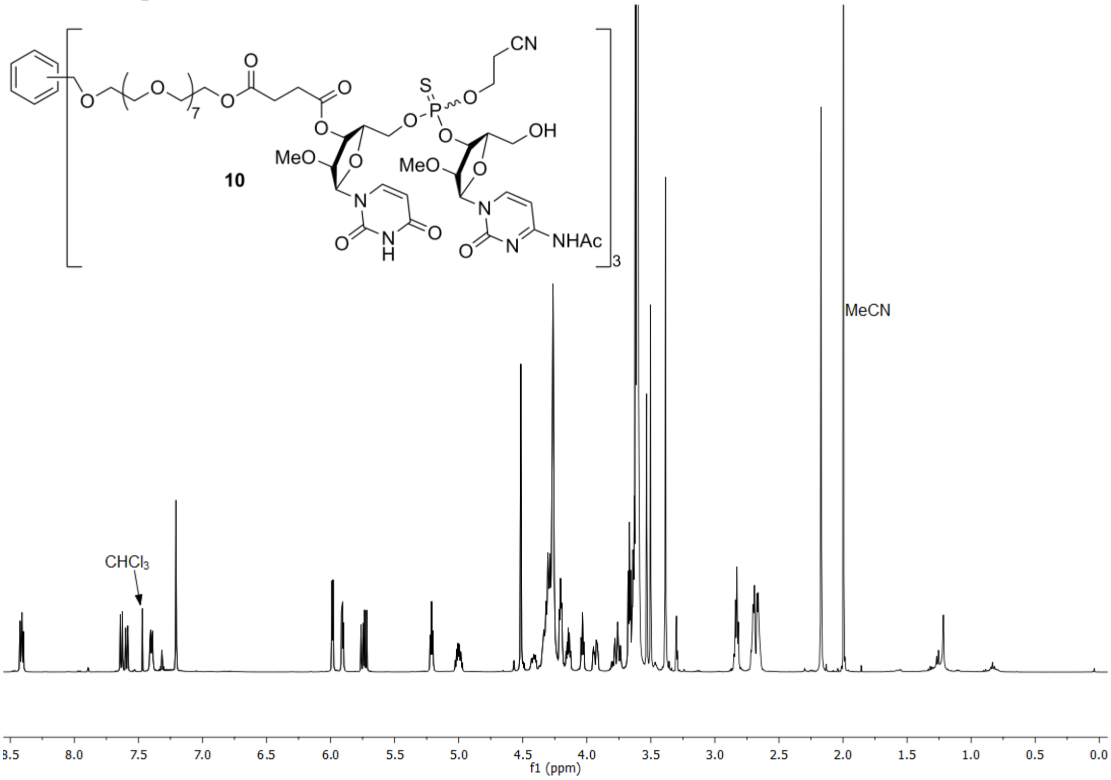

Figure S3.6.1 ^{1}H-NMR spectrum

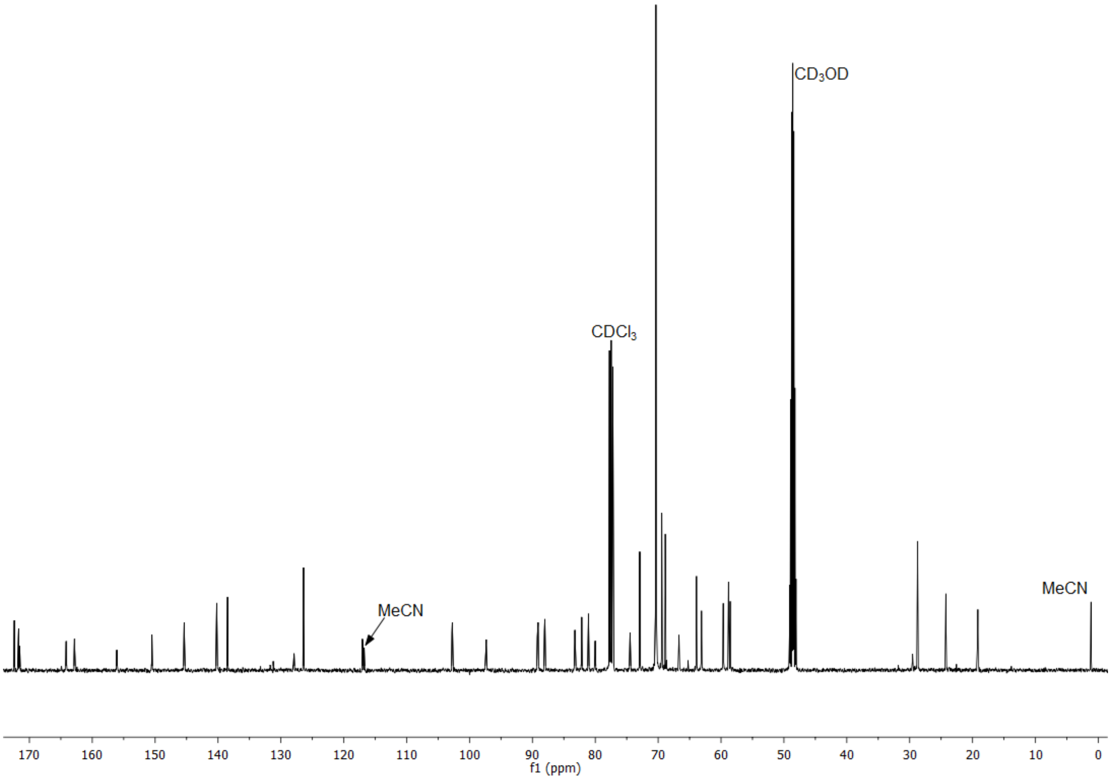

Figure S3.6.2 ^{13}C-NMR spectrum

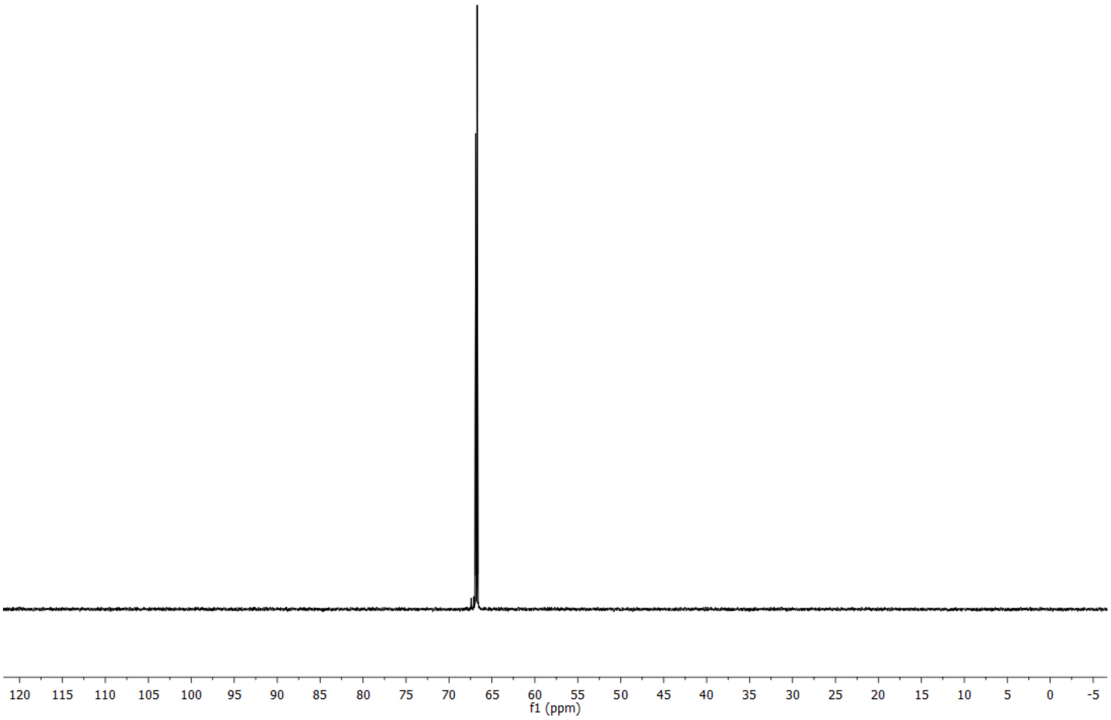

Figure S3.6.3 ^{31}P-NMR spectrum

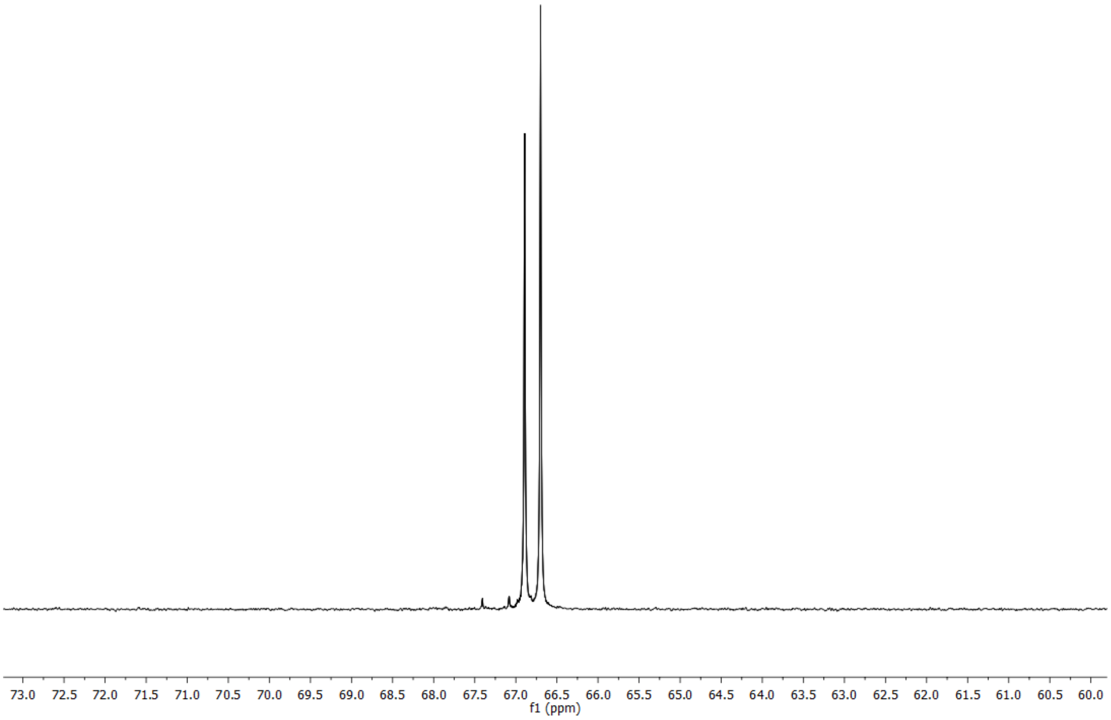

Figure S3.6.4 ^{31}P-NMR spectrum

4. ^{1}H & ^{31}P NMR of homostars purified by OSN

4.1 Crude tris(mUp^{Cne}SmC^{Ac}-ODmtr) homostar, 7 (400 MHz, D_6-acetone).

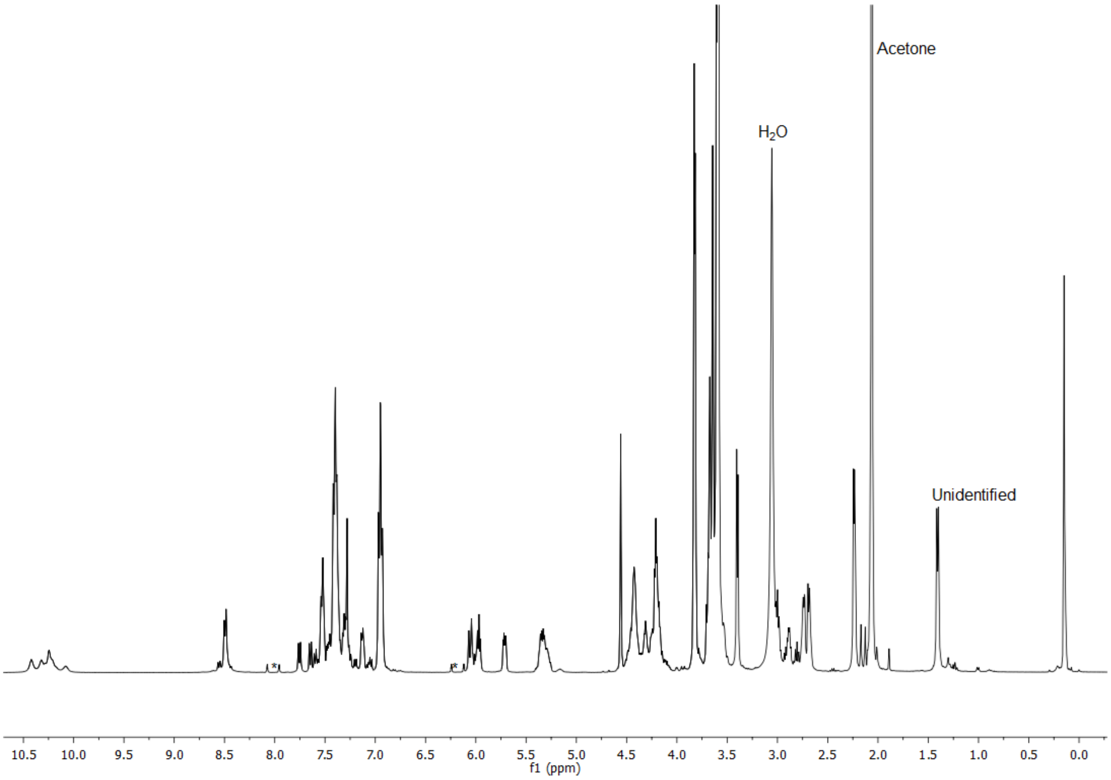

Figure S4.1.1 ^{1}H-NMR spectrum * H-phosphonate derived from hydrolysis of phosphoramidite **6^{c}** sometimes observed.

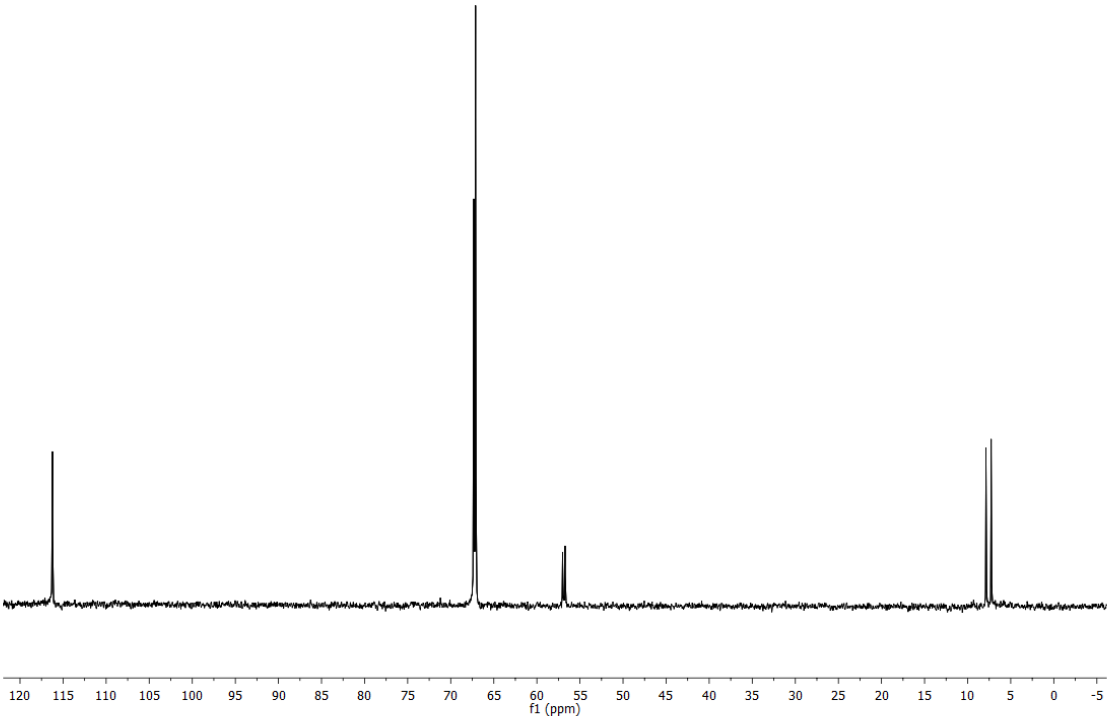

Figure S4.1.2 ^{31}P-NMR spectrum H-phosphonate derived from hydrolysis of phosphoramidite **6^{c}** has identical ^{31}P NMR

4.2 Tris(mUp^{Cne}SmC^{Ac}-OH) homostar, 10 (500 MHz, $CDCl_3$-CD_3OD 2:1).

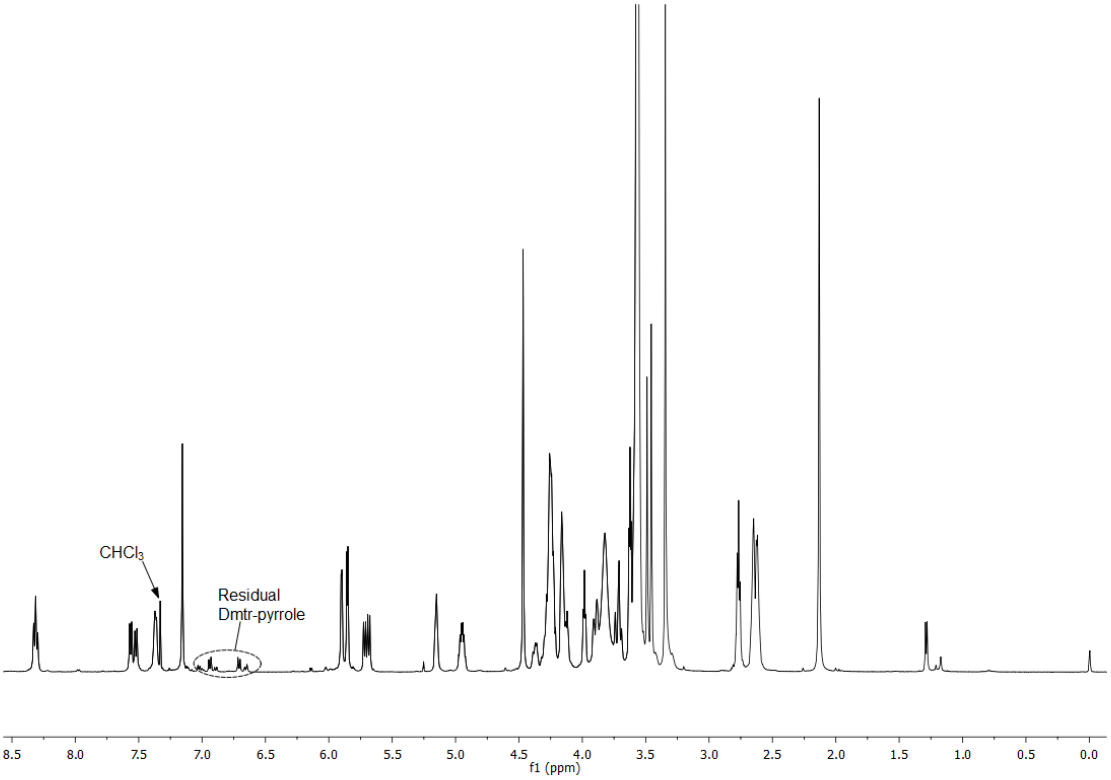

Figure S4.2.1 ^{1}H-NMR spectrum With the exception of a trace of Dmtr-pyrrole, this sample of dinucleotidyl homostar **10** purified by OSN is virtually indistinguishable from that obtained by chromatography (see Figure S3.6.1).

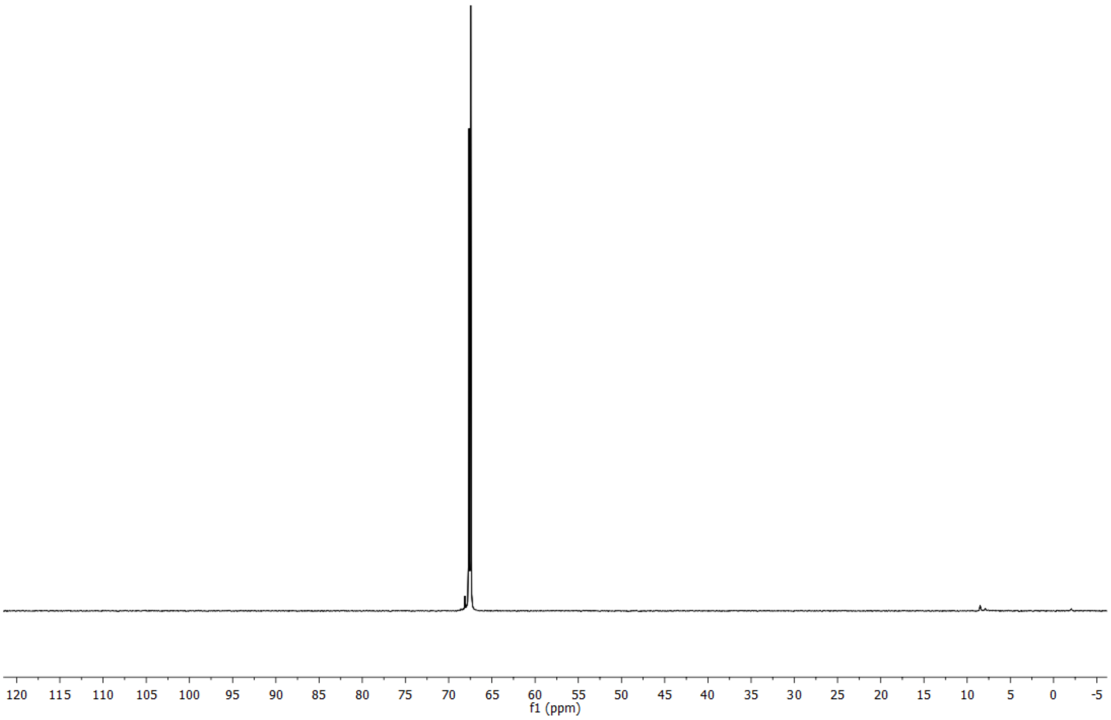

Figure S4.2.2 ^{31}P-NMR spectrum

4.3 Crude tris(mUp^{Cne}SmC^{Ac}p^{Cne}SmC^{Ac}-ODmtr) homostar, 13 (400 MHz, D_6-acetone).

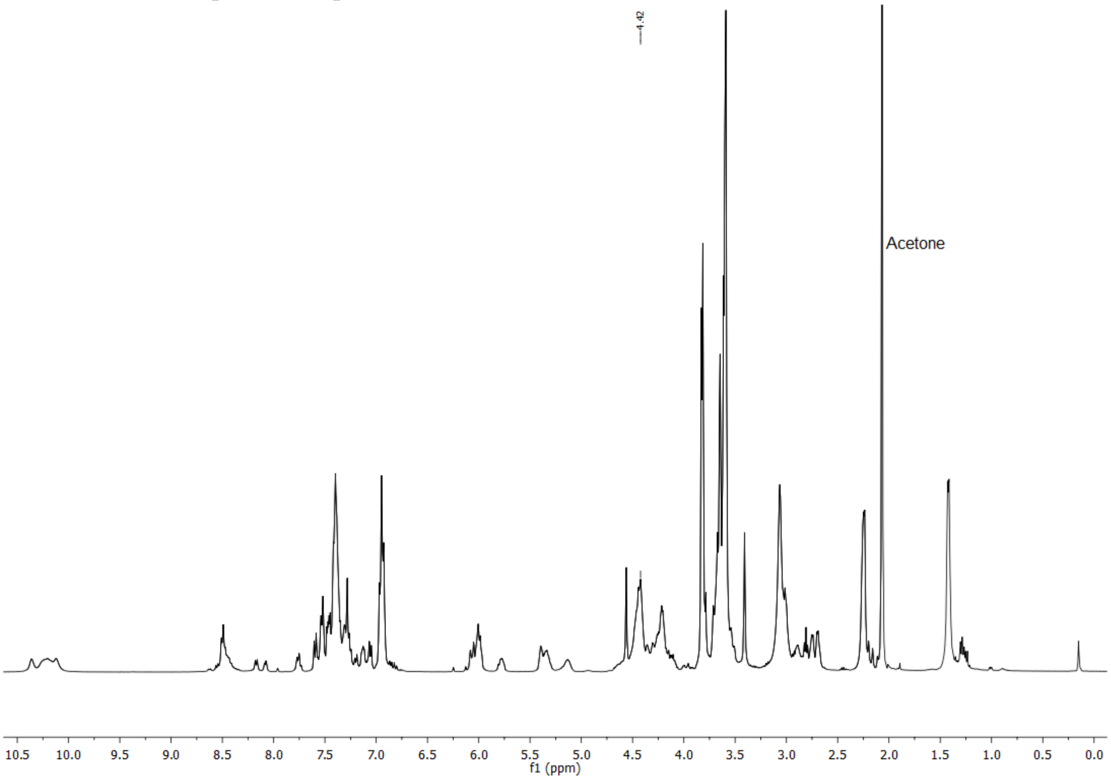

Figure S4.3.1 ^{1}H-NMR spectrum

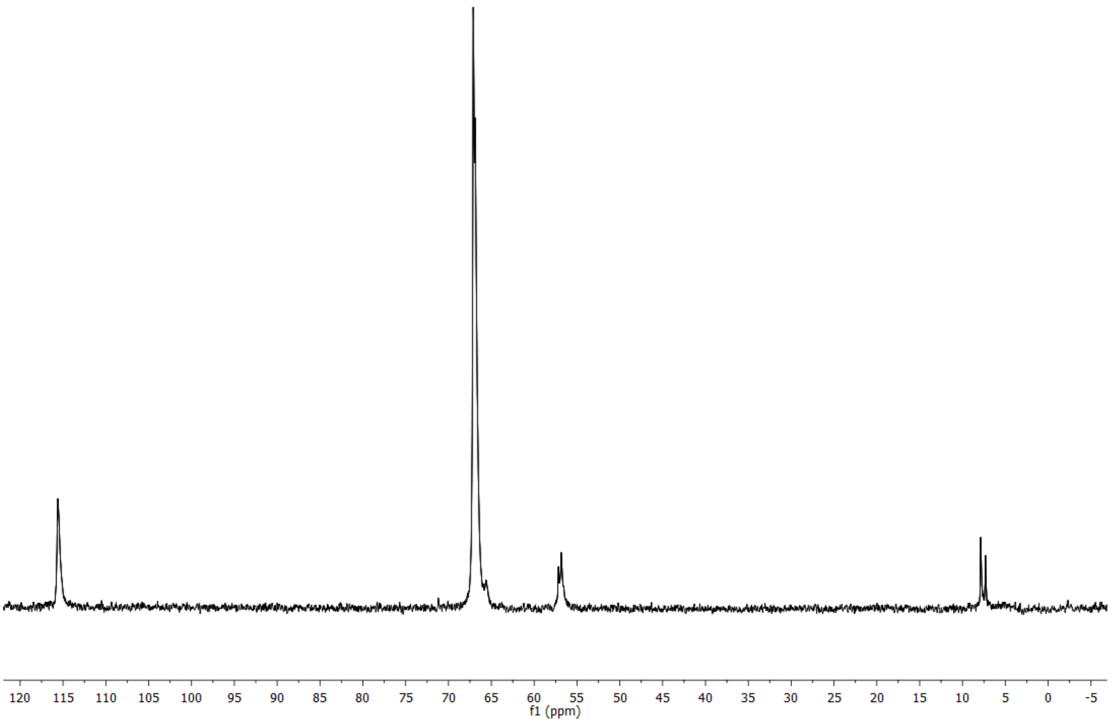

Figure S4.3.2 ^{31}P-NMR spectrum

4.4 Tris(mUp^{Cne}SmC^{Ac}p^{Cne}SmC^{Ac}-OH) homostar, 14 (500 MHz,$CDCl_3$-CD_3OD 3:1), before trituration**.**

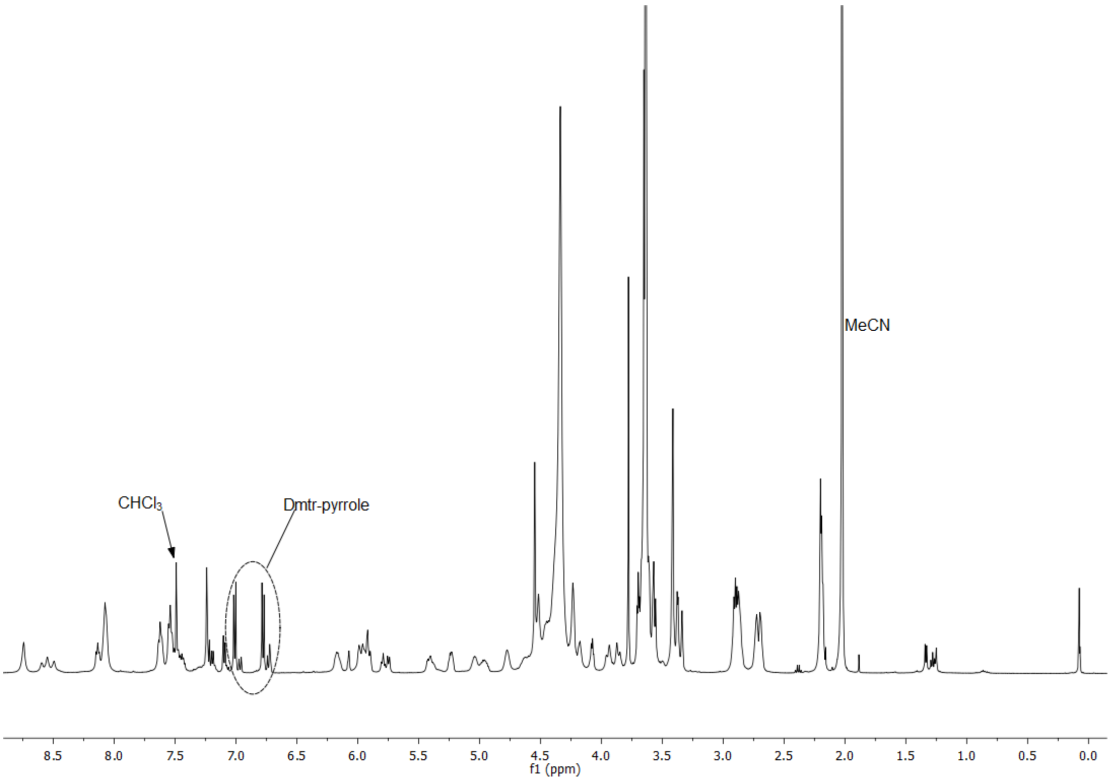

Figure S4.4.1 ^{1}H-NMR spectrum

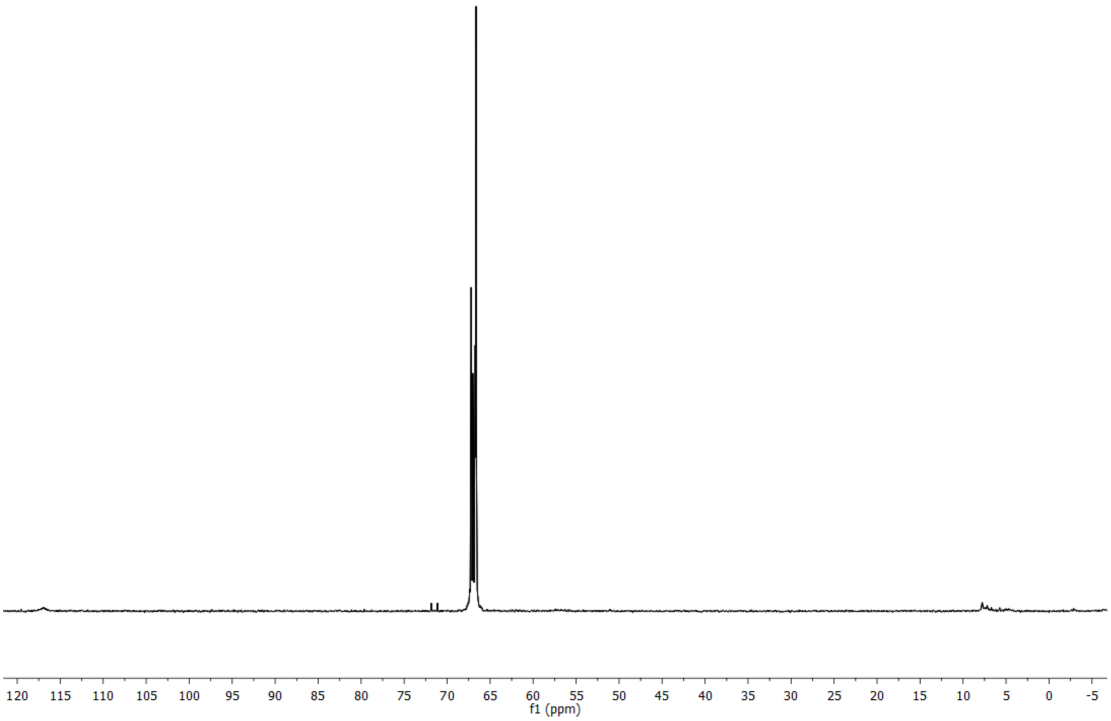

Figure S4.4.2 ^{31}P-NMR spectrum

4.5 Crude tris(mUp^{Cne}SmC^{Ac}p^{Cne}SmC^{Ac}p^{Cne}SmA^{Bz}-ODmtr) homostar, 15 (400 MHz, $CDCl_3$).

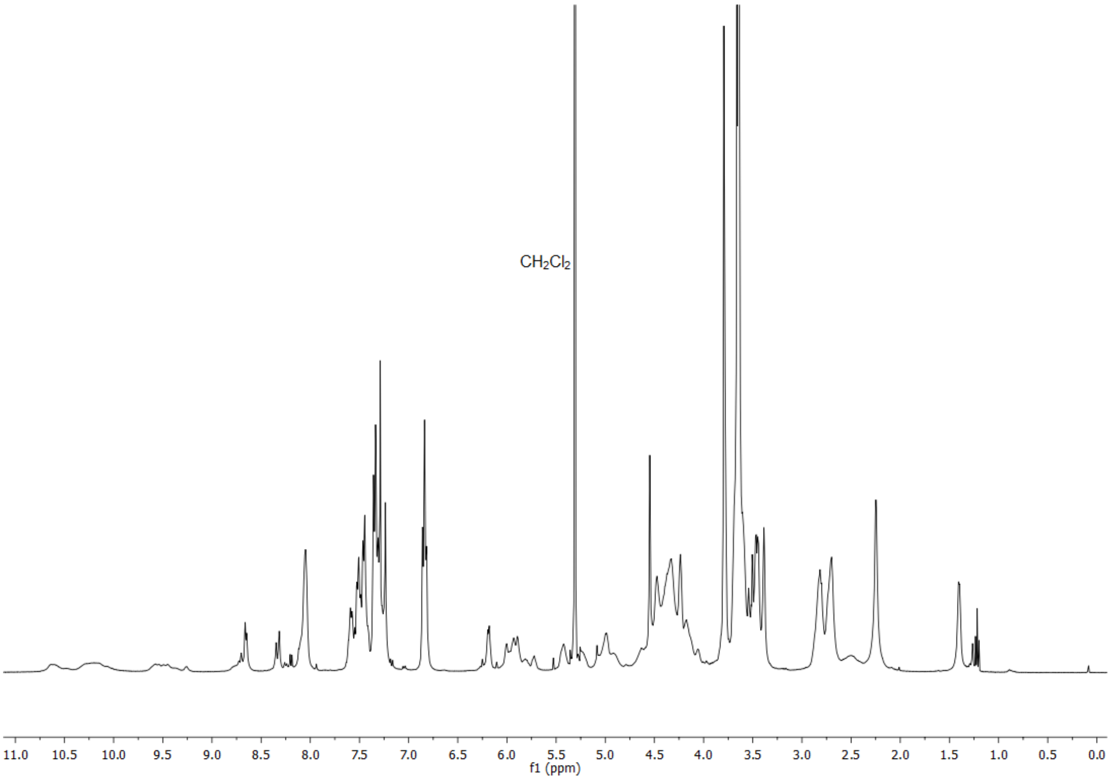

Figure S4.5.1 ^{1}H-NMR spectrum

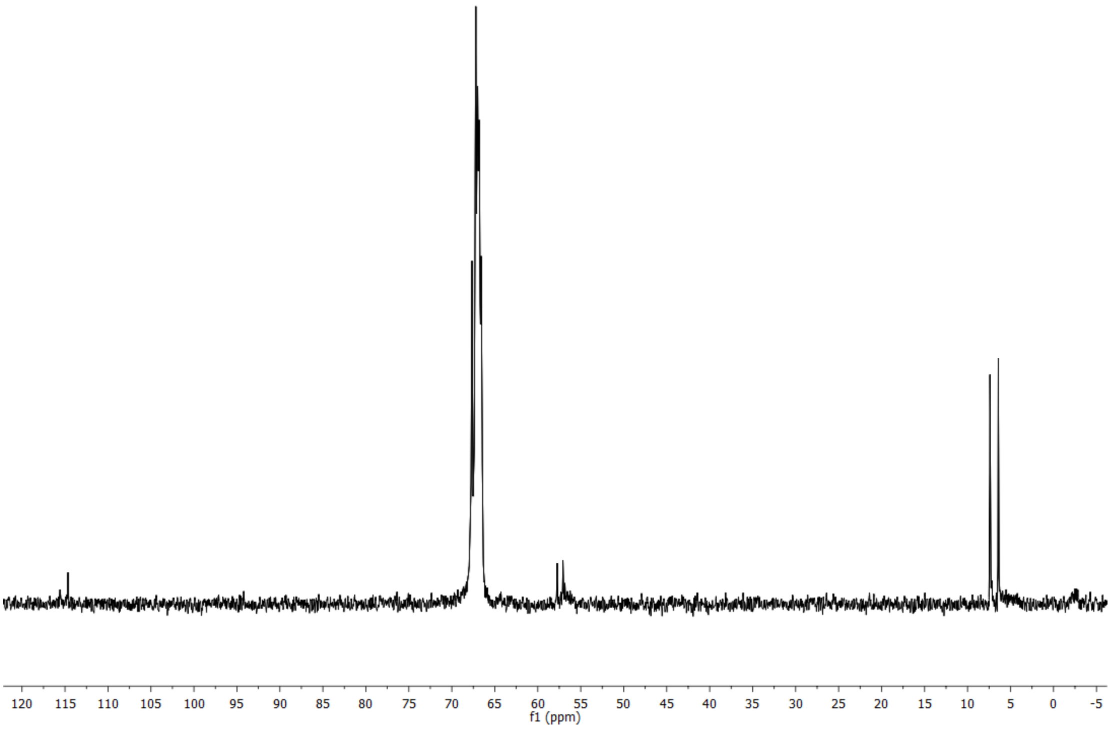

Figure S4.5.2 ^{31}P-NMR spectrum

4.6 Tris(mUp^{Cne}SmC^{Ac}p^{Cne}SmC^{Ac}p^{Cne}SmA^{Bz}-OH) homostar, 16 (500 MHz, $CDCl_3$-CD_3OD 3:1) before trituration**.**

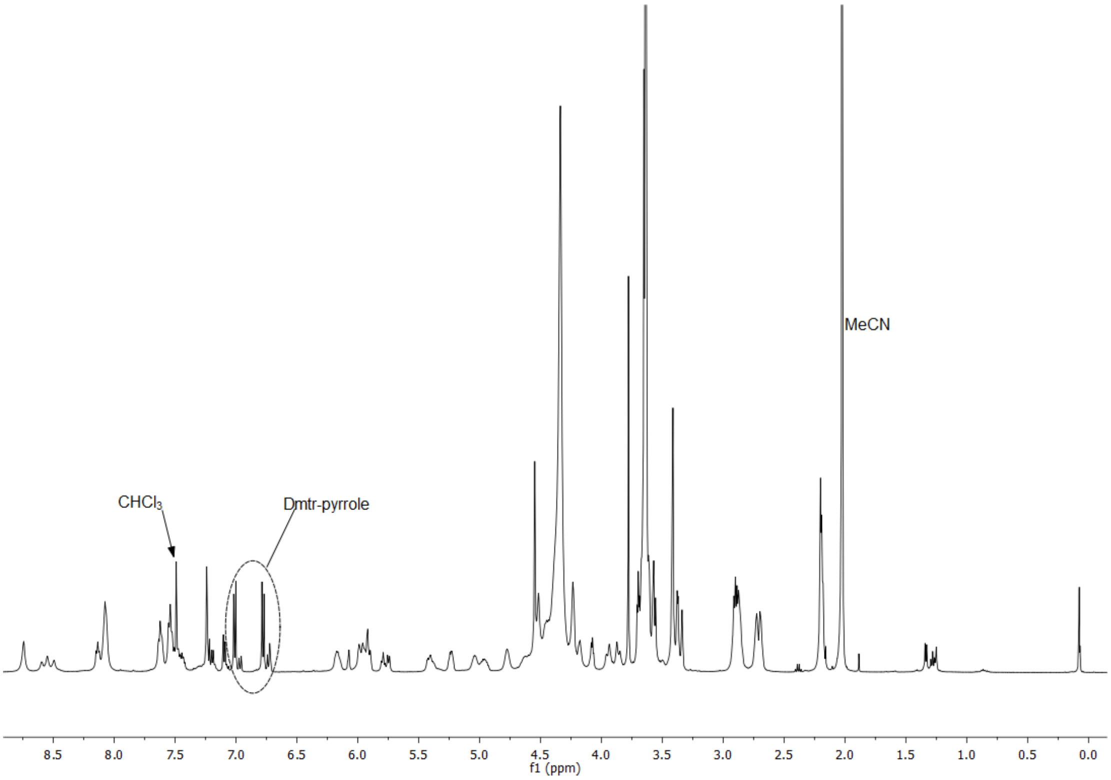

Figure S4.6.1 ^{1}H-NMR spectrum

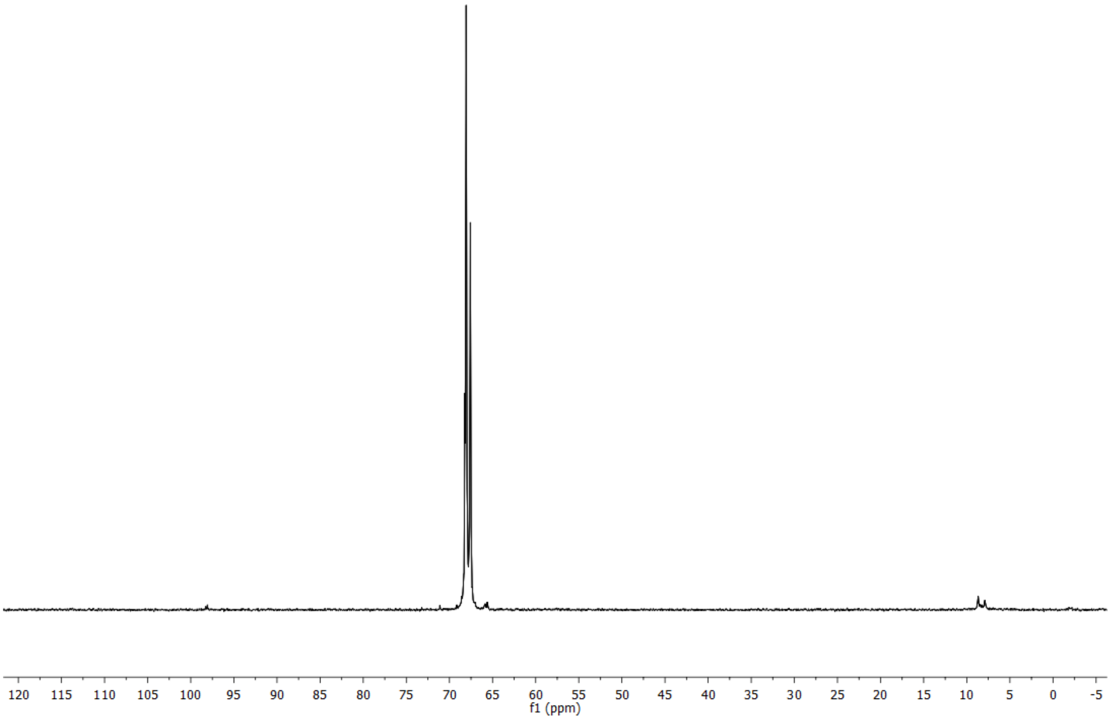

Figure S4.6.2 ^{31}P-NMR spectrum

4.7 Crude tris(mUp^{Cne}SmC^{Ac}p^{Cne}SmC^{Ac}p^{Cne}SmA^{Bz}p^{Cne}SmU-ODmtr) homostar, 17 (400 MHz, $CDCl_3$).

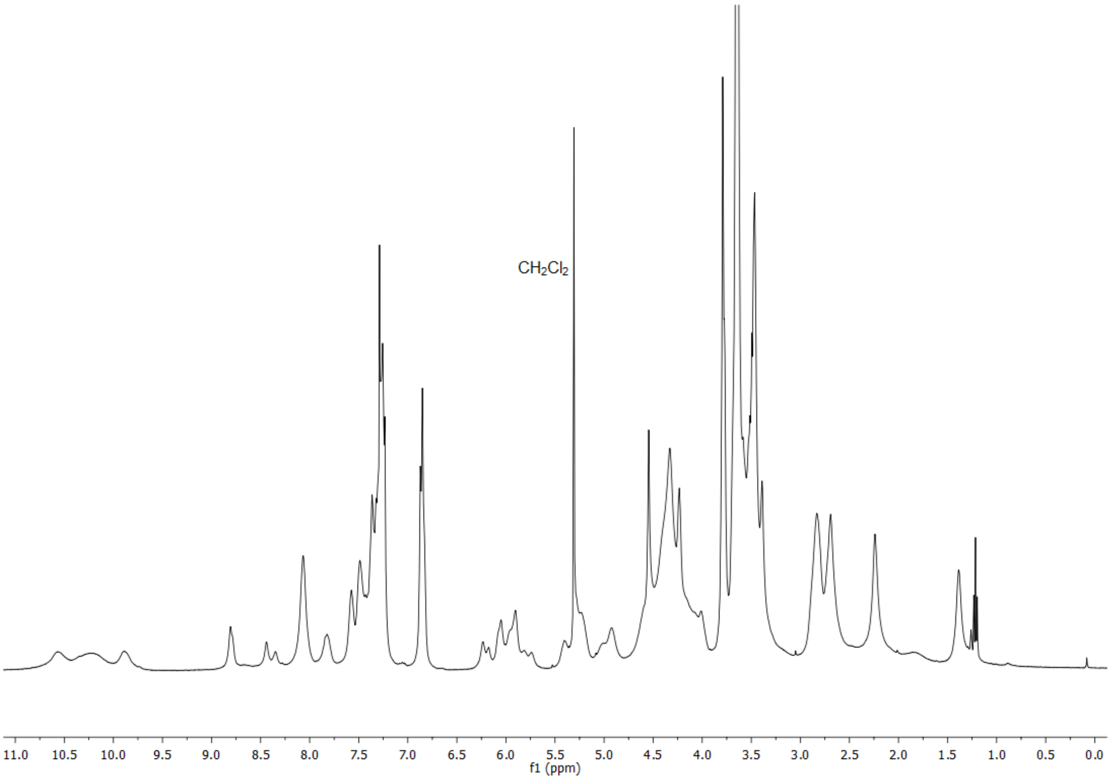

Figure S4.7.1 ^{1}H-NMR spectrum

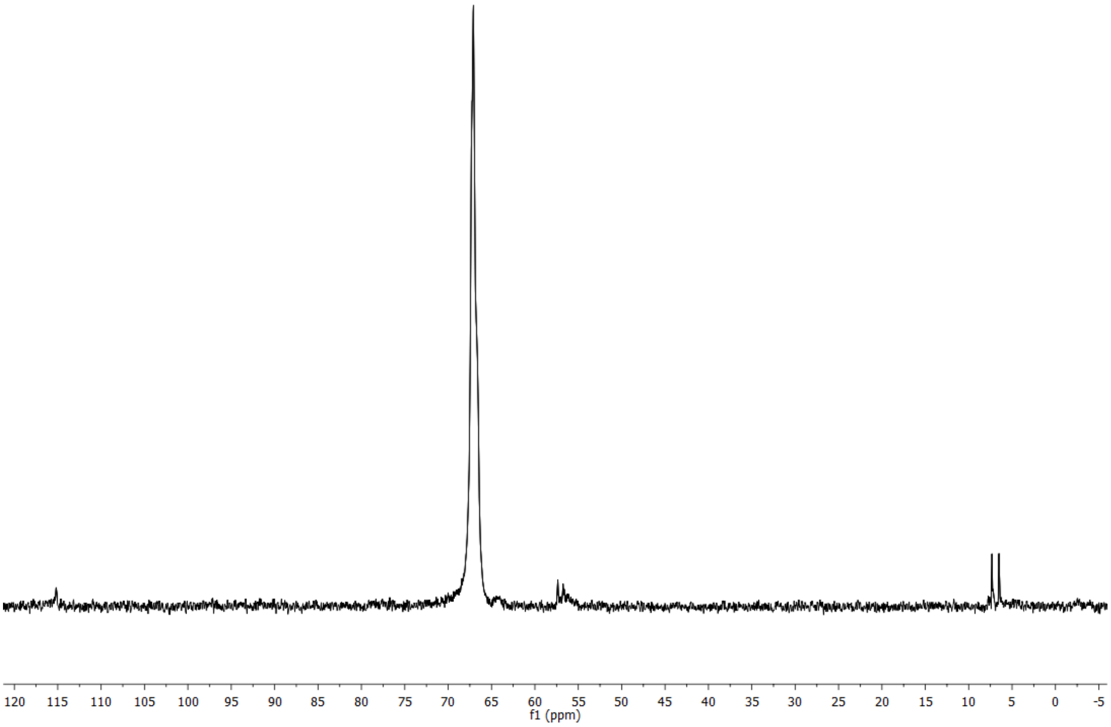

Figure S4.7.2 ^{31}P-NMR spectrum

4.8 Tris(mUp^{Cne}SmC^{Ac}p^{Cne}SmC^{Ac}p^{Cne}SmA^{Bz}p^{Cne}SmU-OH) homostar, 18 (400 MHz, $CDCl_3$-CD_3OD 3:1).

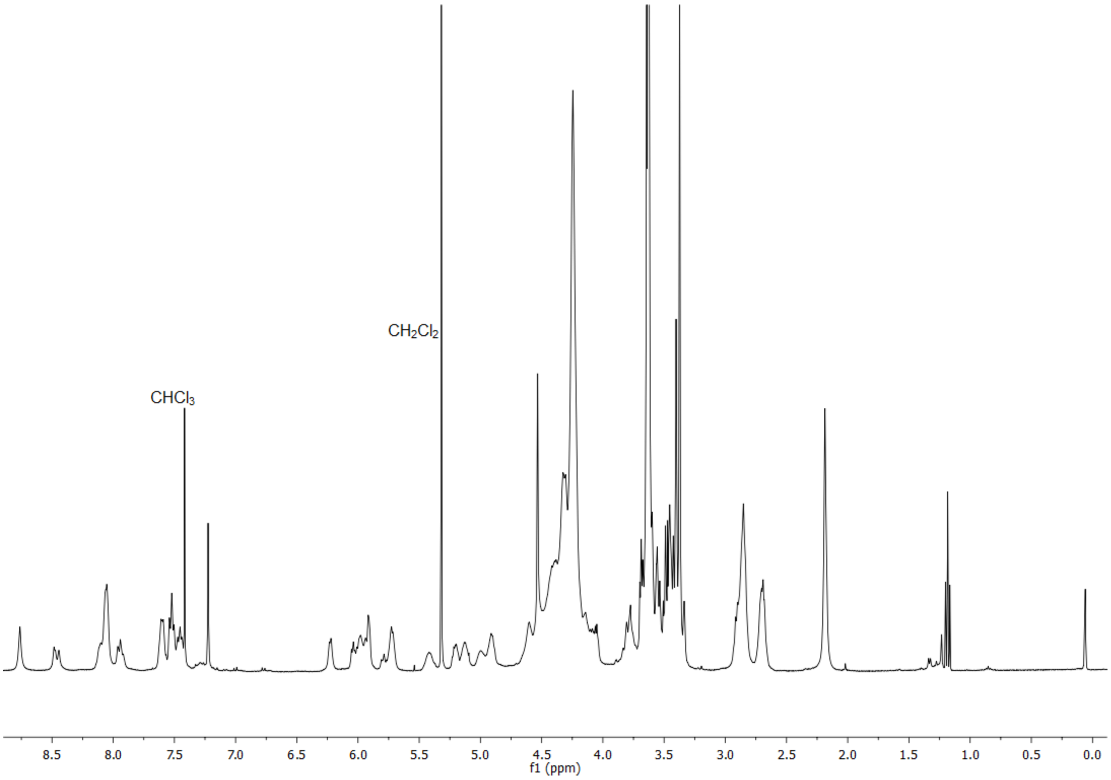

Figure S4.8.1 ^{1}H-NMR spectrum

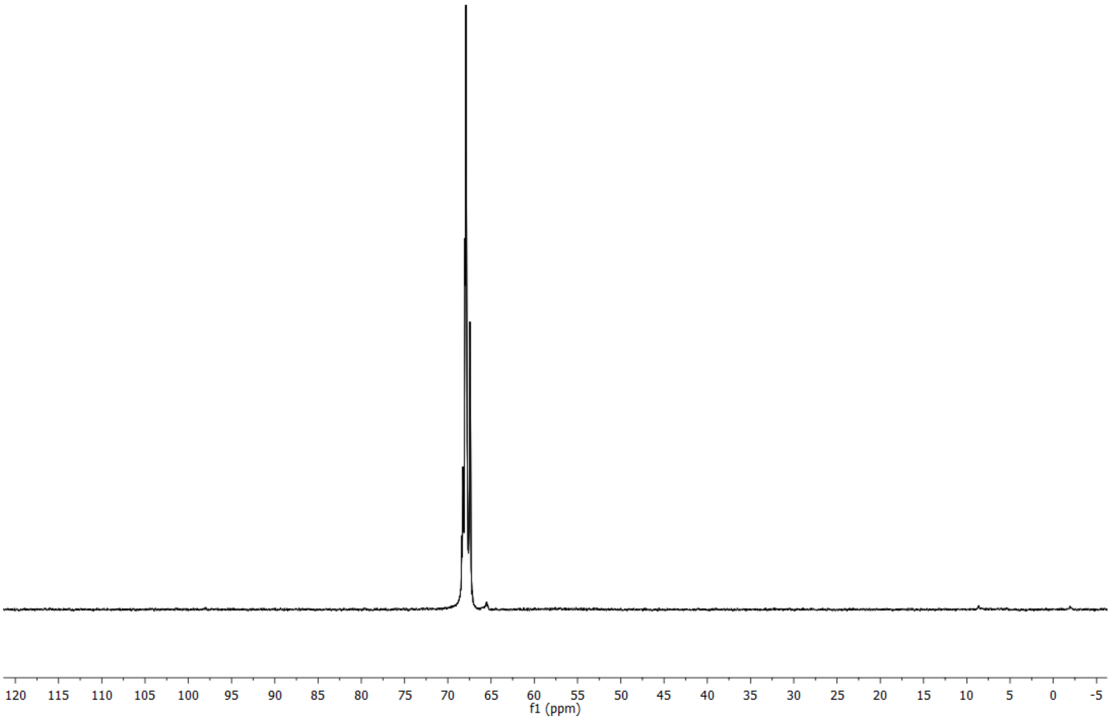

Figure S4.8.2 ^{31}P-NMR spectrum

4.9 Crude tris(mUp^{Cne}SmC^{Ac}p^{Cne}SmC^{Ac}p^{Cne}SmA^{Bz}p^{Cne}SmUp^{Cne}SmU-ODmtr) homostar, 20 (500 MHz, D_6-acetone-CD_3OD 3:1).

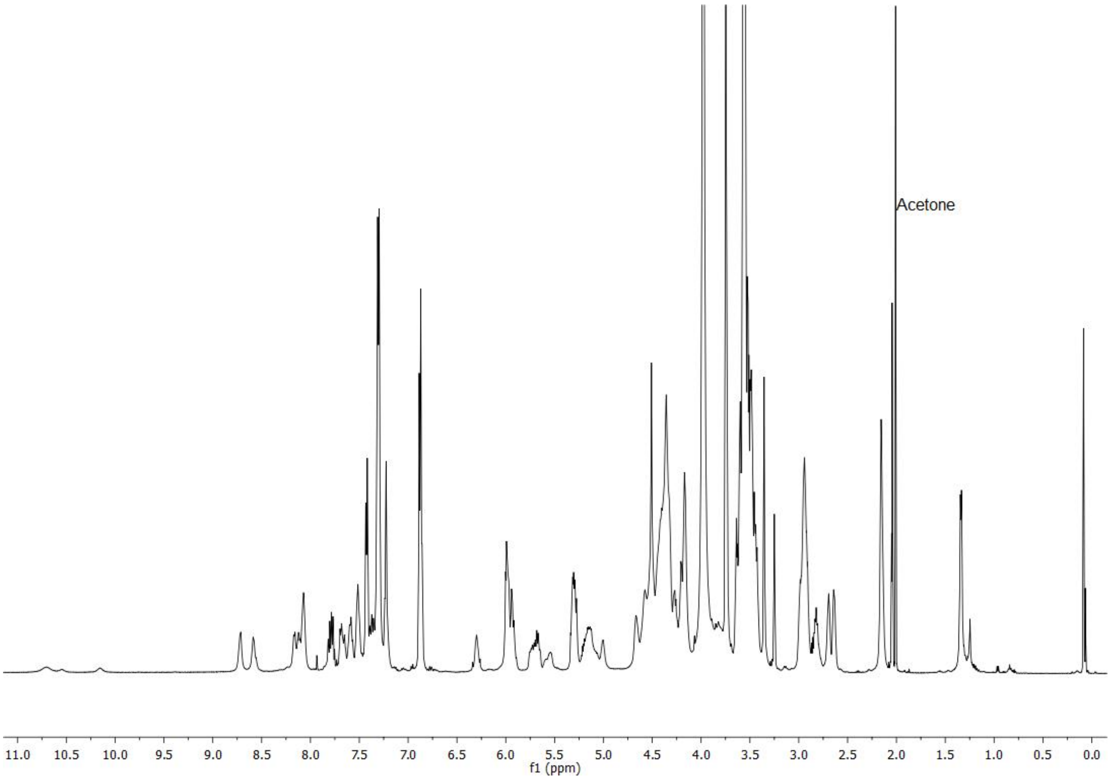

Figure S4.9.1 ^{1}H-NMR spectrum

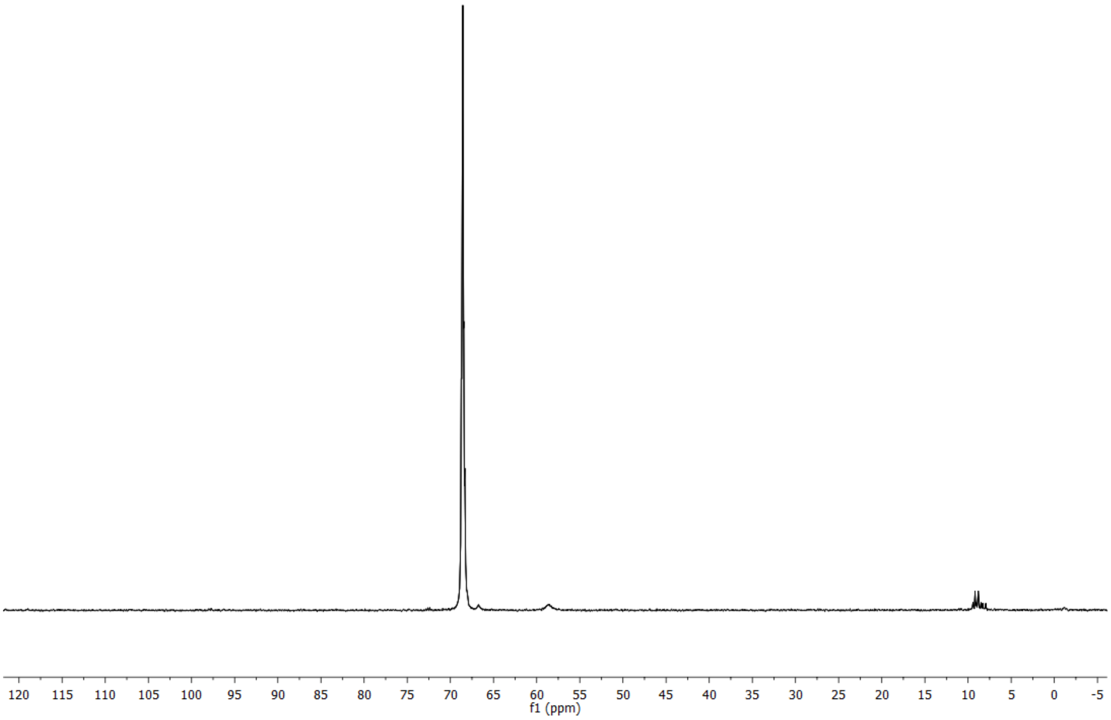

Figure S4.9.2 ^{31}P-NMR spectrum

4.10 Tris(mUp^{Cne}SmC^{Ac}p^{Cne}SmC^{Ac}p^{Cne}SmA^{Bz}p^{Cne}SmUp^{Cne}SmU-OH) homostar, 21 (500 MHz, $CDCl_3$-CD_3OD 3:1).

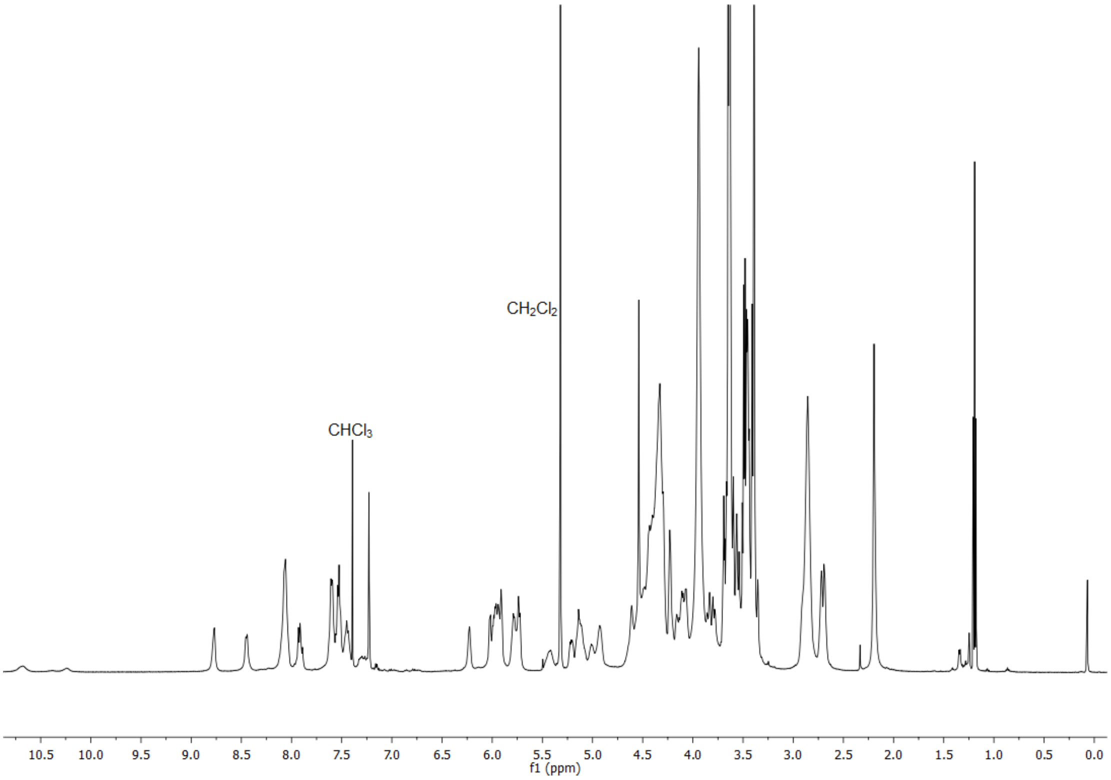

Figure S4.10.1 ^{1}H-NMR spectrum

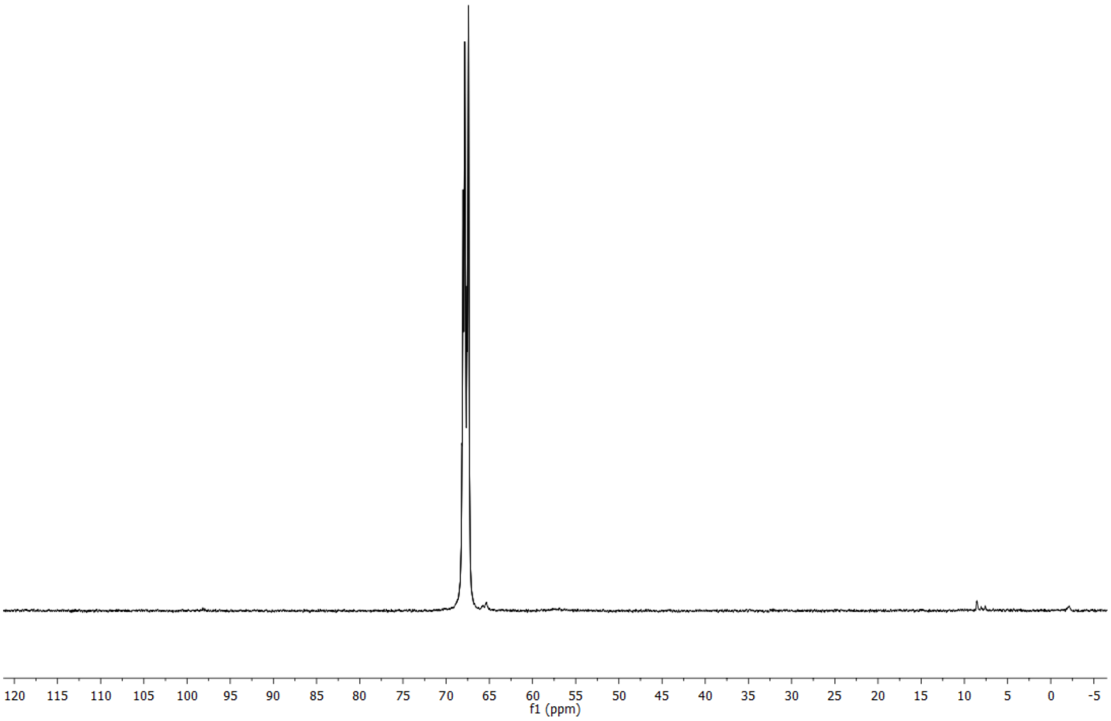

Figure S4.10.2 ^{31}P-NMR spectrum

4.11 Crude tris(mUp^{Cne}SmC^{Ac}p^{Cne}SmC^{Ac}p^{Cne}SmA^{Bz}p^{Cne}SmUp^{Cne}SmUp^{Cne}SmC^{Ac}-ODmtr) homostar, 22 (400 MHz, D_6-acetone CD_3OD 3:1).

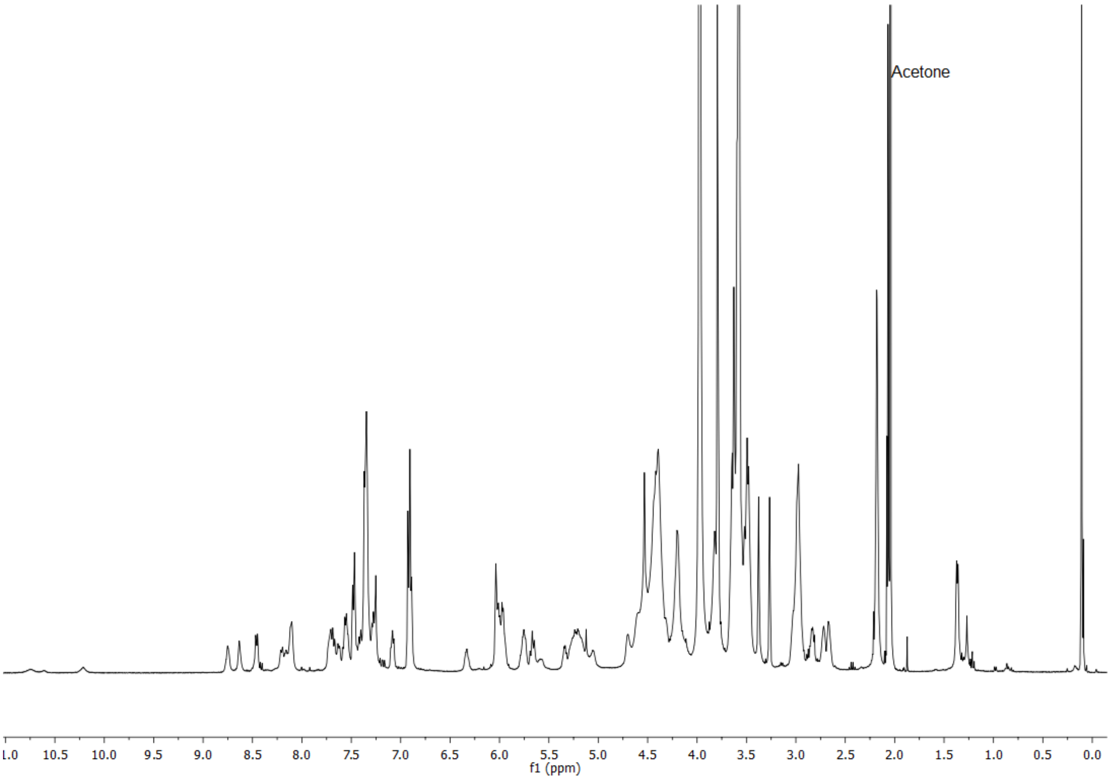

Figure S4.11.1 ^{1}H-NMR spectrum

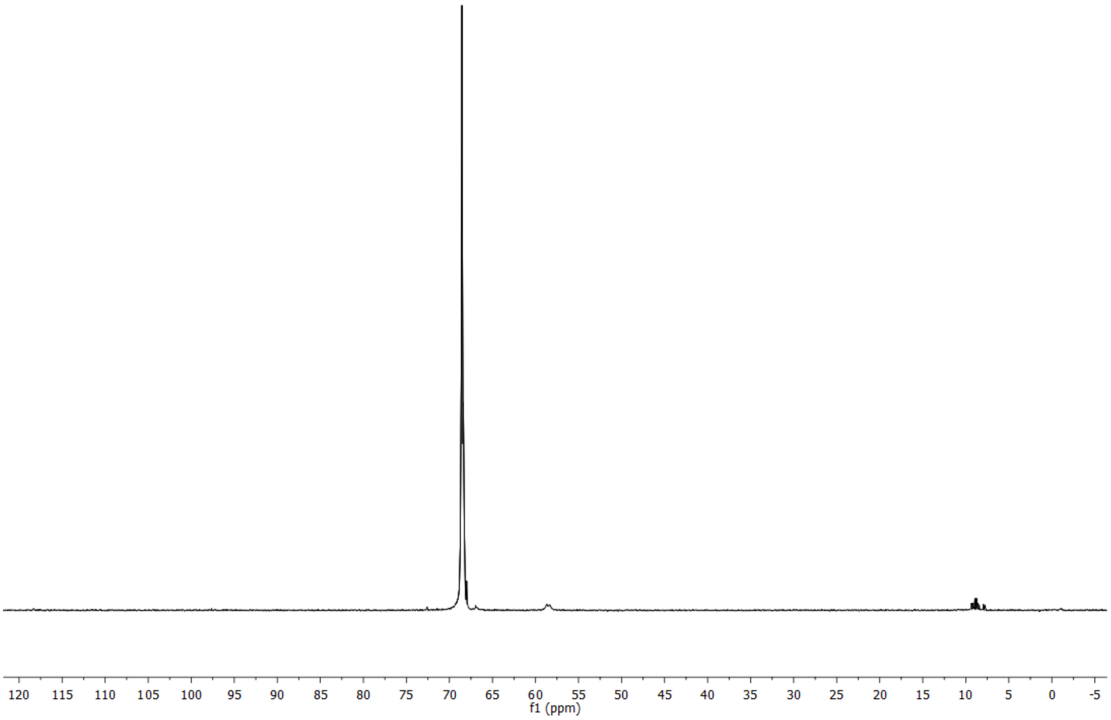

Figure S4.11.2 ^{31}P-NMR spectrum

4.12 Tris(mUp^{Cne}SmC^{Ac}p^{Cne}SmC^{Ac}p^{Cne}SmA^{Bz}p^{Cne}SmUp^{Cne}SmUp^{Cne}SmC^{Ac}-OH) homostar, 23 21 (500 MHz, $CDCl_3$-CD_3OD 3:1).

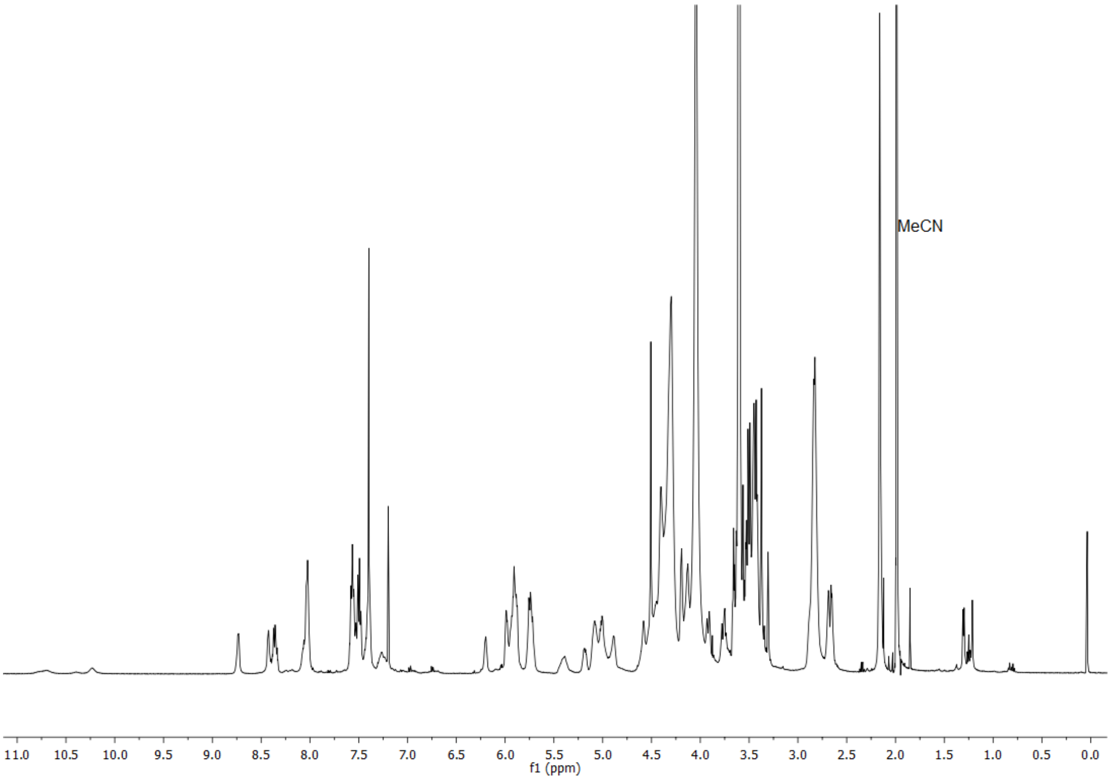

Figure S4.12.1 ^{1}H-NMR spectrum

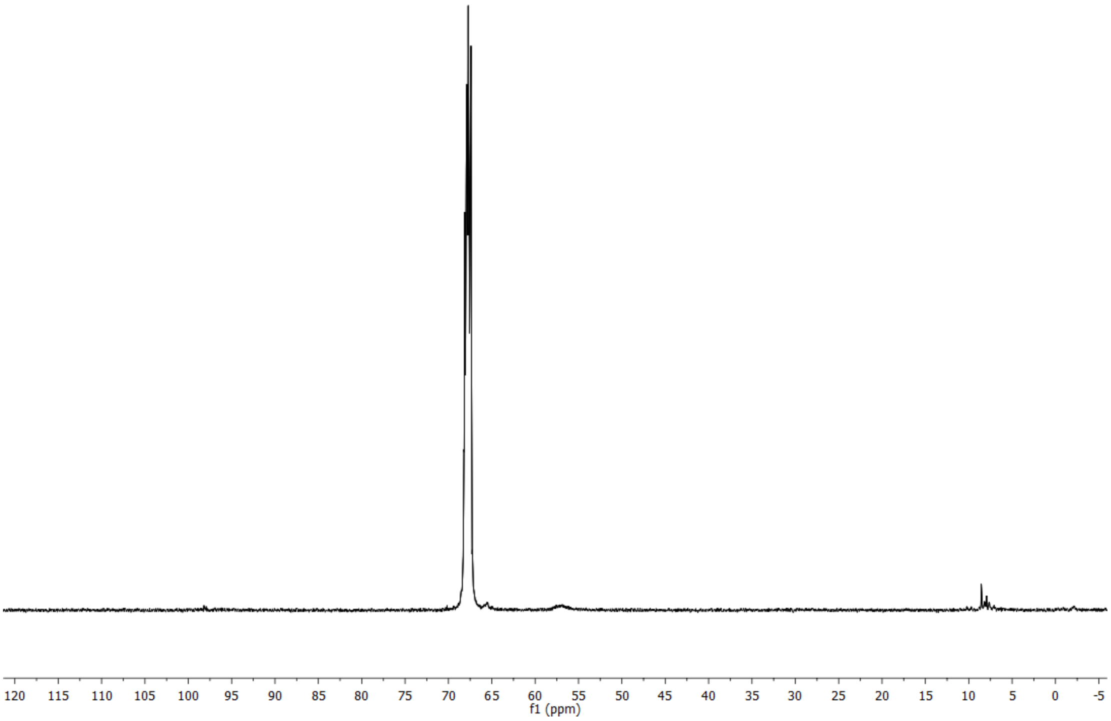

Figure S4.12.2 ^{31}P-NMR spectrum

4.13 Crude tris(mUp^{Cne}SmC^{Ac}p^{Cne}SmC^{Ac}p^{Cne}SmA^{Bz}p^{Cne}SmUp^{Cne}SmUp^{Cne}SmC^{Ac}p^{Cne}SmG^{iBu}-ODmtr) homostar, 24.

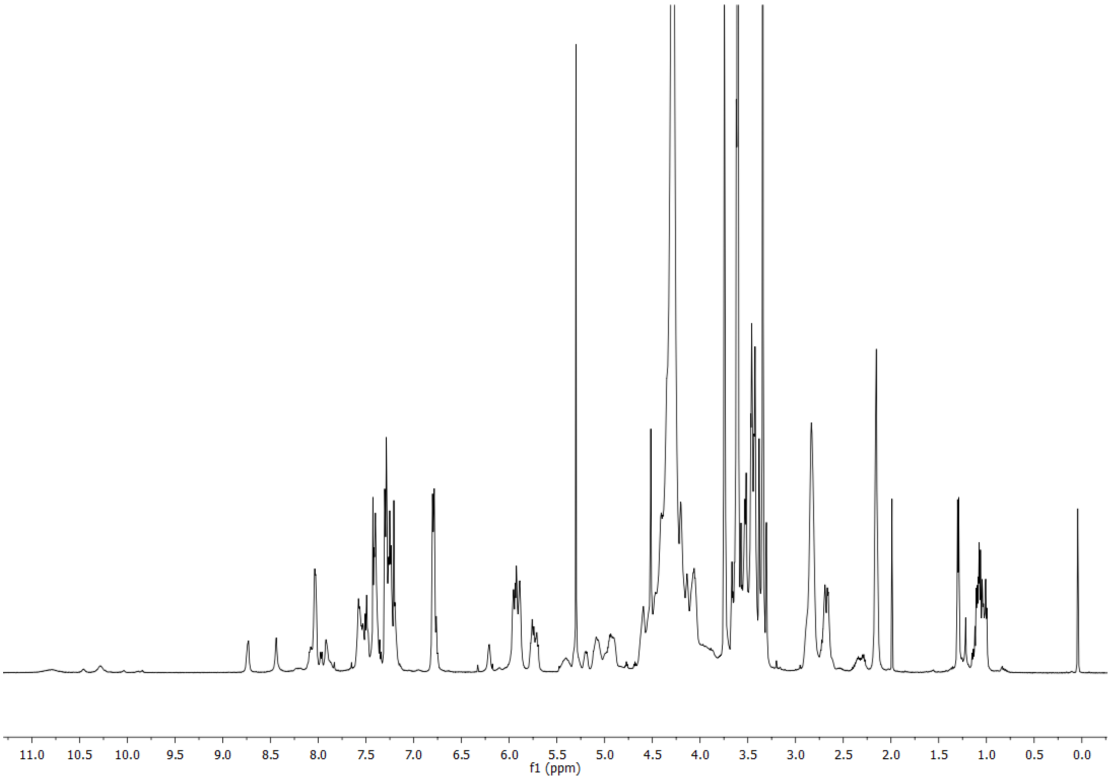

Figure S4.13.1 ^{1}H-NMR spectrum

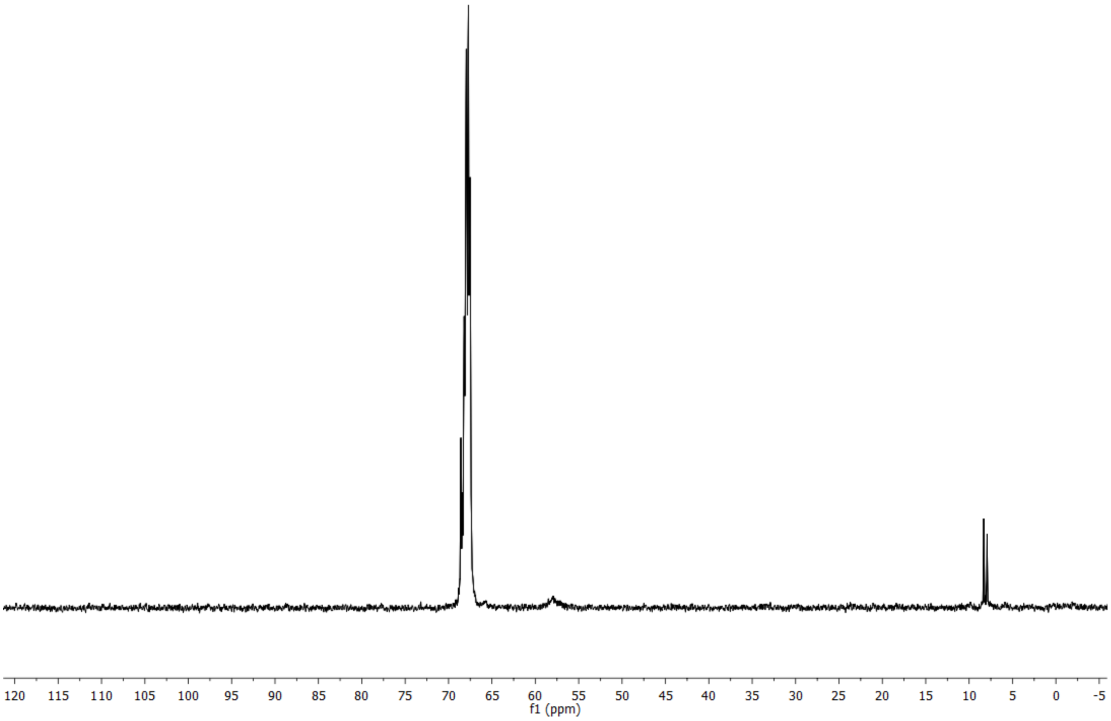

Figure S4.13.2 ^{31}P-NMR spectrum

4.14 Tris(mUp^{Cne}SmC^{Ac}p^{Cne}SmC^{Ac}p^{Cne}SmA^{Bz}p^{Cne}SmUp^{Cne}SmUp^{Cne}SmC^{Ac}p^{Cne}SmG^{iBu}-OH) homostar, 25.

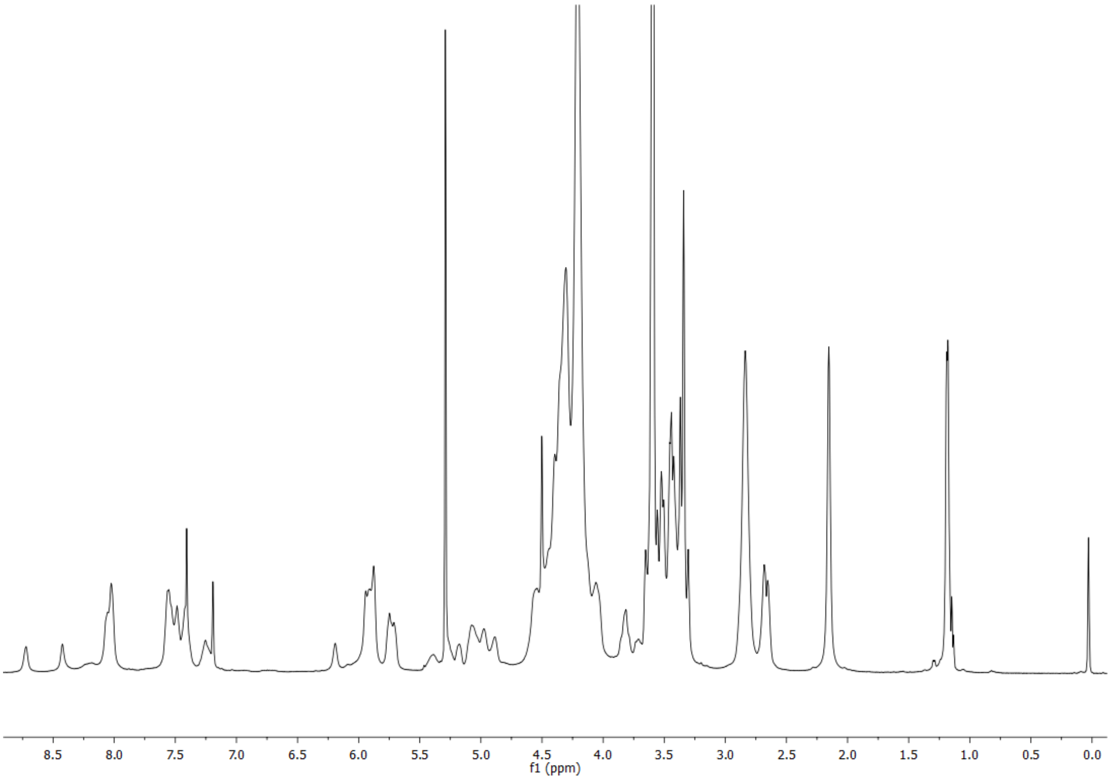

Figure S4.14.1 ^{1}H-NMR spectrum

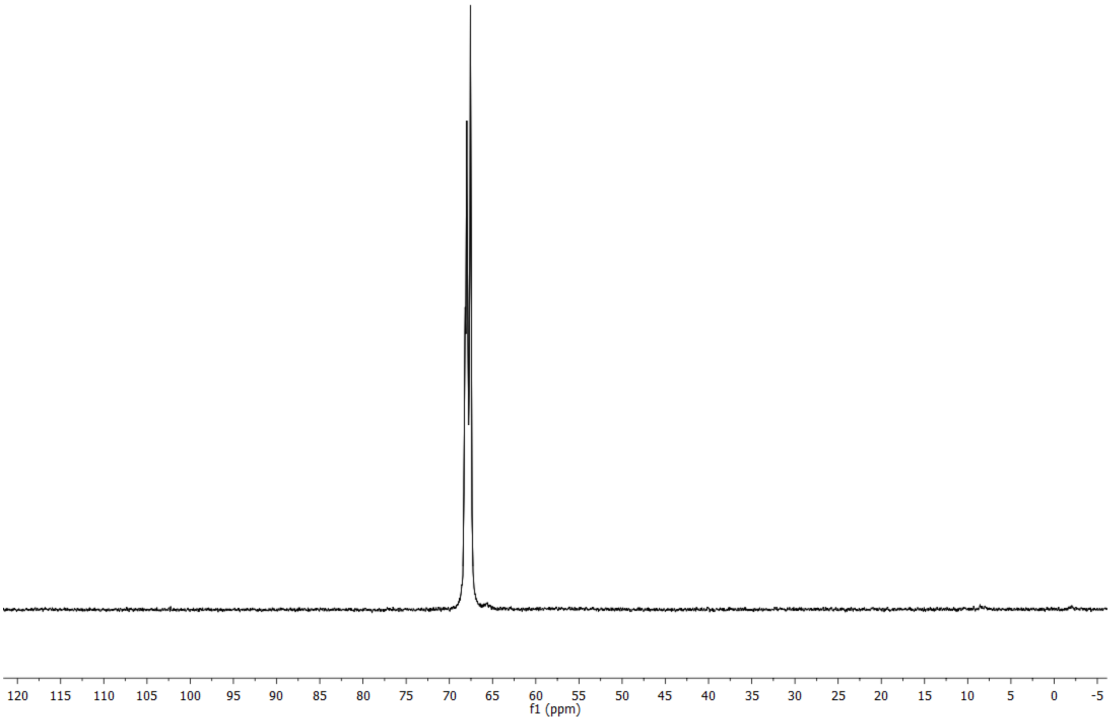

Figure S4.14.2 ^{31}P-NMR spectrum

4.15 Crude tris(mUp^{Cne}SmC^{Ac}p^{Cne}SmC^{Ac}p^{Cne}SmA^{Bz}p^{Cne}SmUp^{Cne}SmUp^{Cne}SmC^{Ac}p^{Cne}S-mG^{iBu}p^{Cne}SmG^{iBu}ODmtr) homostar, 26.

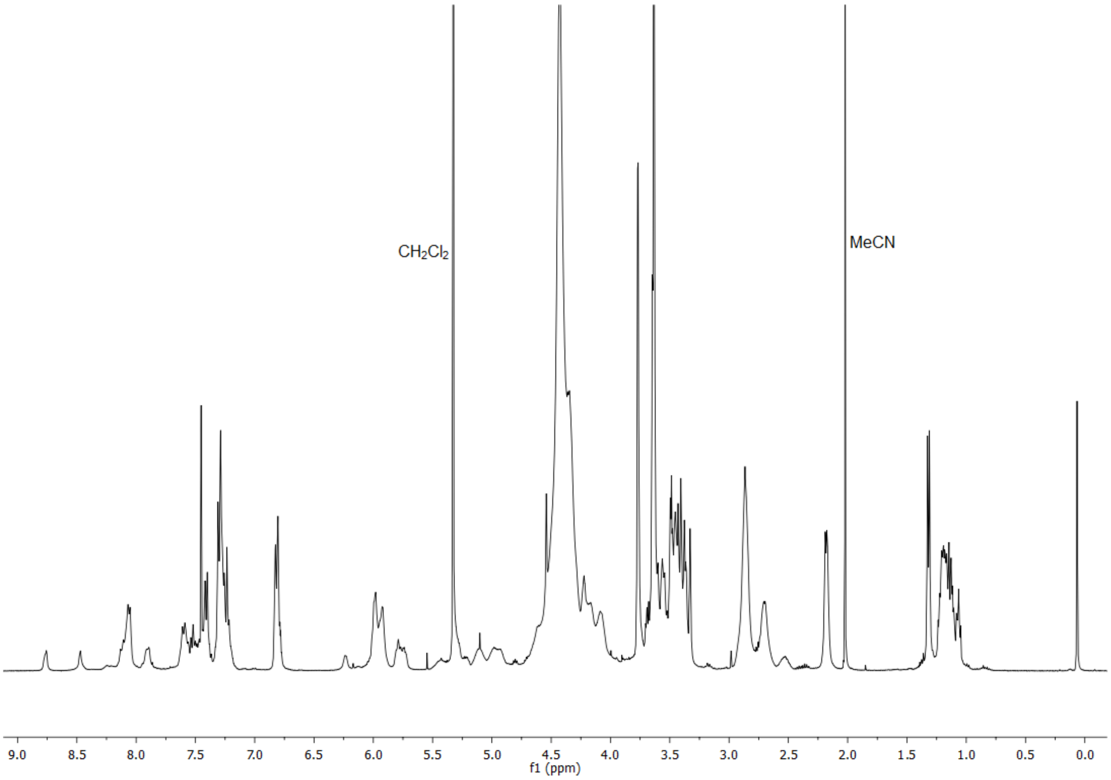

Figure S4.15.1 ^{1}H-NMR spectrum

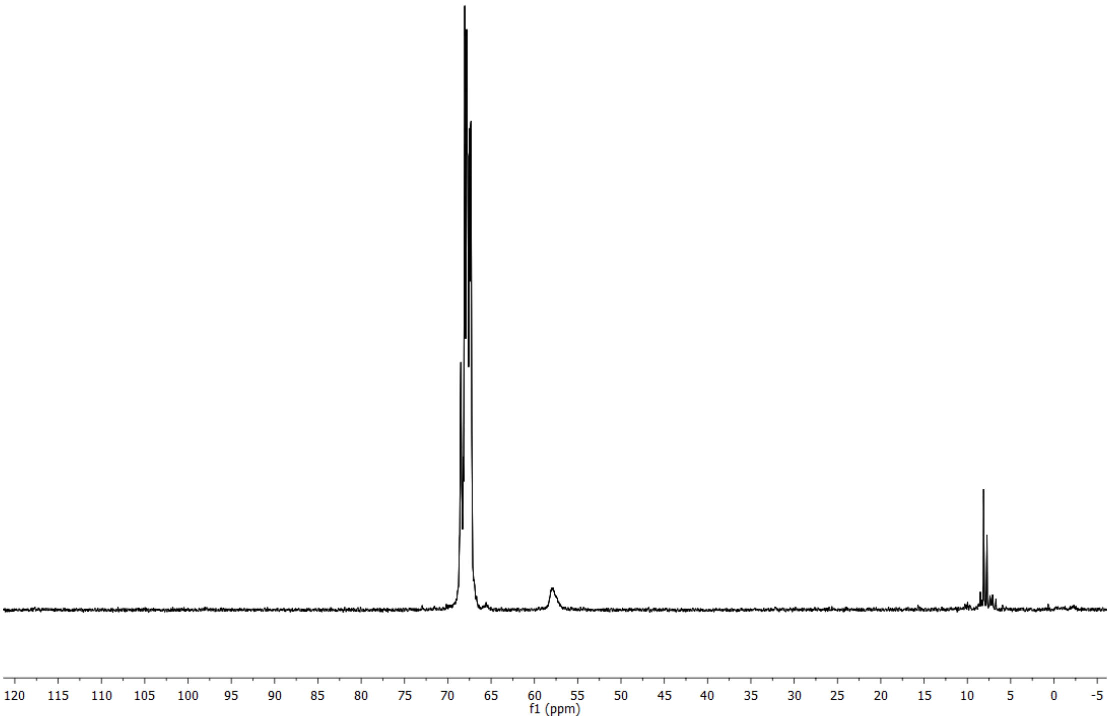

Figure S4.15.2 ^{31}P-NMR spectrum

4.16 Tris(mUp^{Cne}SmC^{Ac}p^{Cne}SmC^{Ac}p^{Cne}SmA^{Bz}p^{Cne}SmUp^{Cne}SmUp^{Cne}SmC^{Ac}p^{Cne}SmG^{iBu}p^{Cne}SmG^{iBu}-OH) homostar, 27.

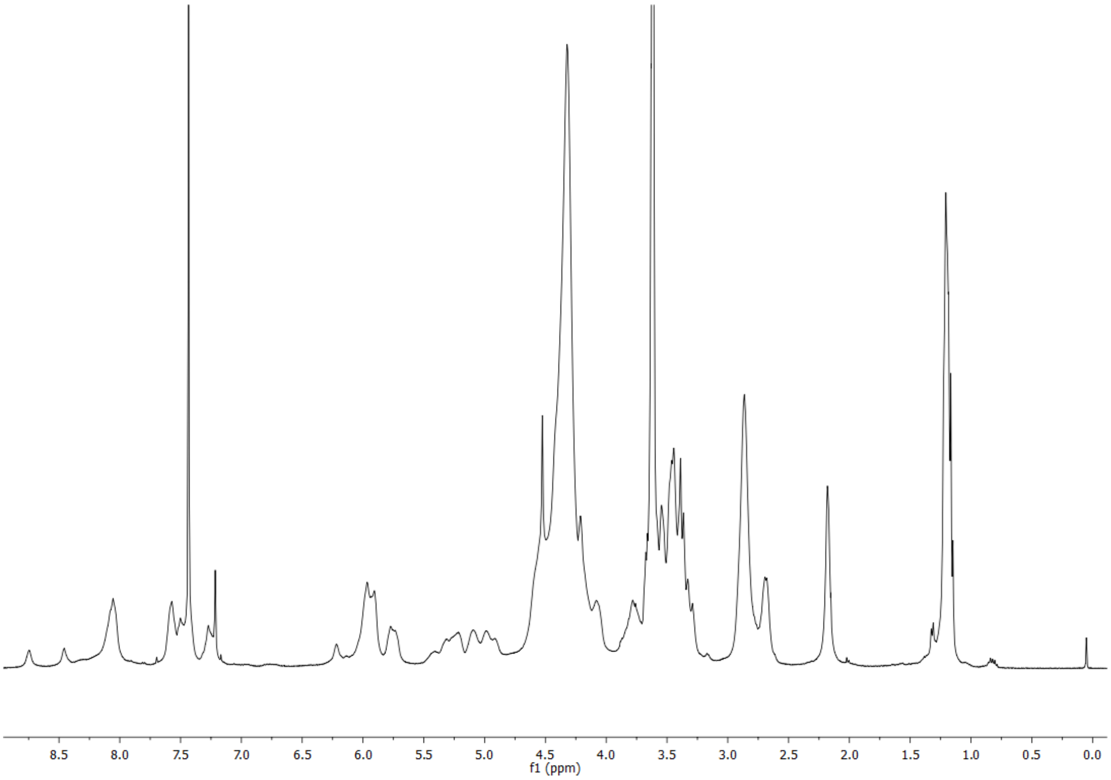

Figure S4.16.1 ^{1}H-NMR spectrum

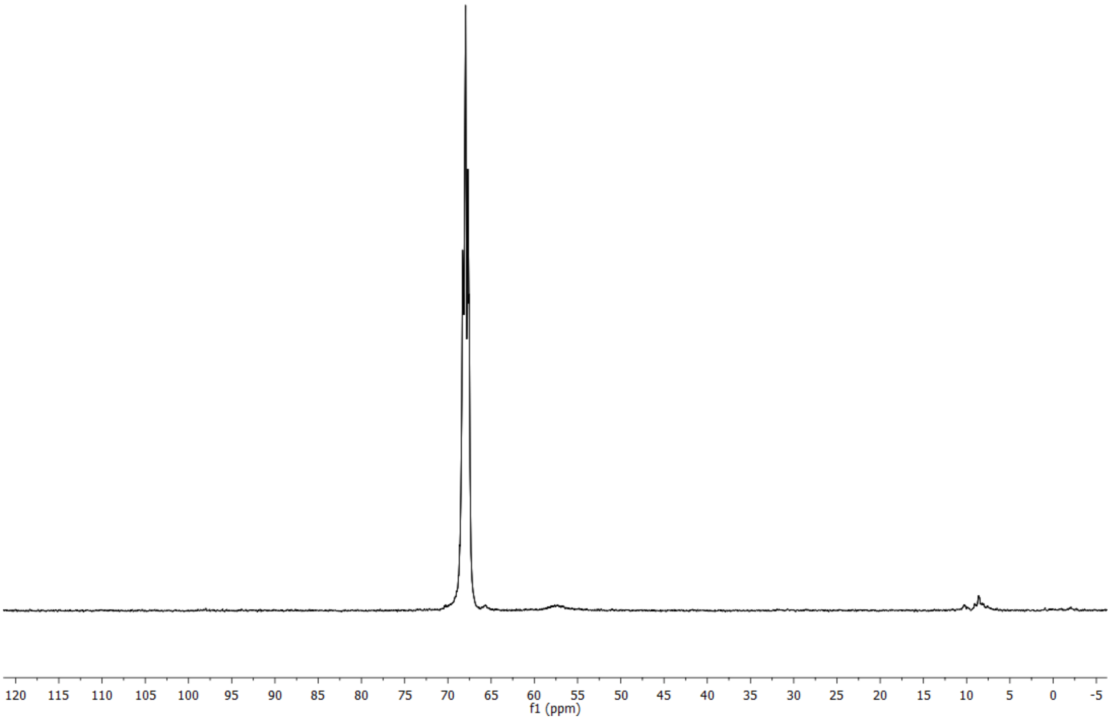

Figure S4.16.2 ^{31}P-NMR spectrum

5. Mass Spectra of homostars

Matrix assisted laser desorption ionisation (MALDI) of oligonucleotidyl homostars was attempted with a wide range of matrices and some variation of the solvent. Initially, oligonucleotidyl homostar samples were dissolved in MeCN-MeOH (1:4 v/v), and then mixed with an equal volume of aqueous matrix solution, but the performance was very variable. Currently we find that best results are achieved by dissolving oligonucleotidyl homostars in MeCN-MeOH, then mixing with a DMF solution of 6-aza-2-thiothymine, and slow evaporation at room temperature to give the target spot.

5.1 Tris(mU-ODmtr) homostar, 3.

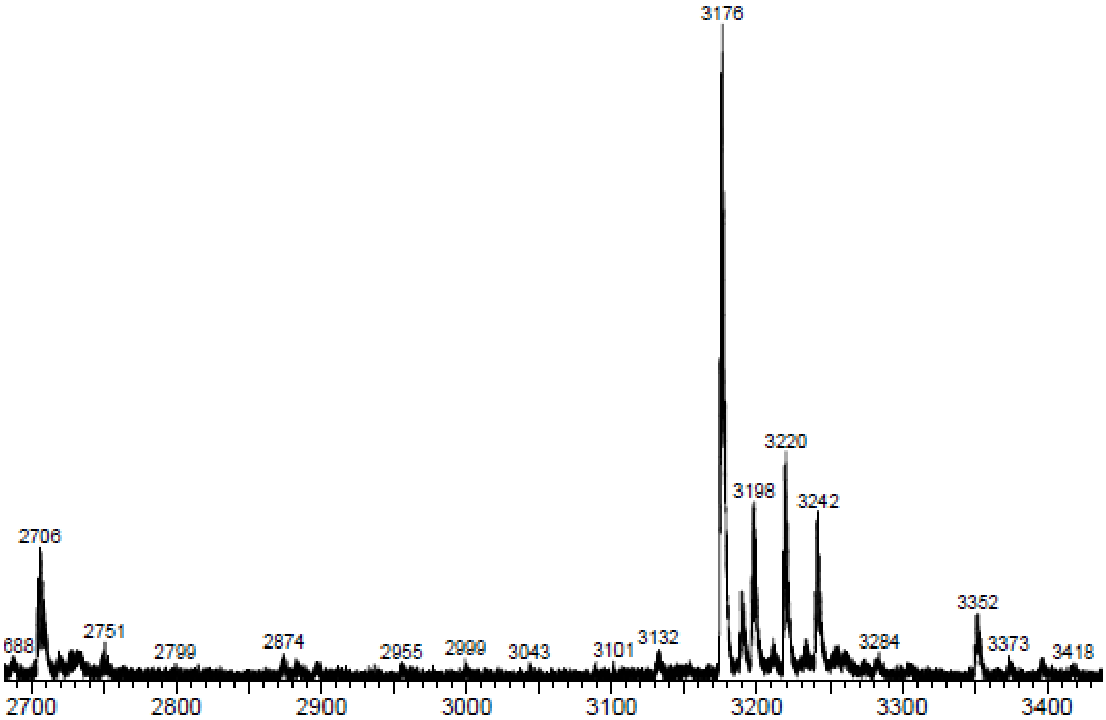

Figure S5.1 *m/z* (MALDI-ToF+) [**3**+Na]$^+$ = 3176, calc. $C_{162}H_{210}N_6NaO_{57}{}^+$ = 3175.4. [**3**–H+2Na]$^+$ = 3198, [**3**–2H+3Na]$^+$ = 3220, and [**3**–3H+4Na]$^+$ = 3242. Peak at *m/z* = 2706 corresponds to Dcb ester on one arm of the homostar which is not separated until after detritylation. Peak at *m/z* = 3352 may corresponds Dcb acylation of uracil, but is not observed after detritylation.

5.2 Tris(mU-OH) homostar, 4.

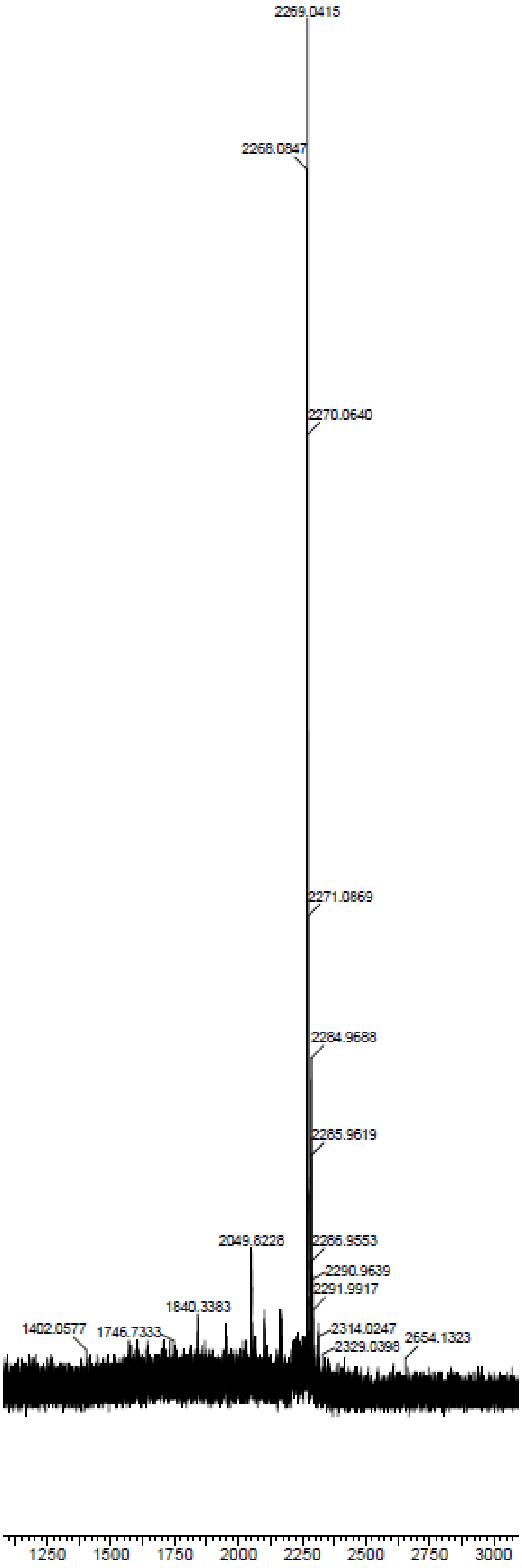

Figure S5.2 *m/z* (MALDI-ToF+) [**4**+Na]$^+$ = 2269.0, calc. $C_{99}H_{156}N_6NaO_{51}^+$ = 2268.97. Peak at *m/z* = 2049.8 corresponds to hydrolysis of succinyl mU ester sodium salt, probably during ionisation, calc. $C_{89}H_{143}N_4Na_2O_{46}^+$ = 2049.88.

5.3 Bis(mU-OH)Dcb homostar, 5.

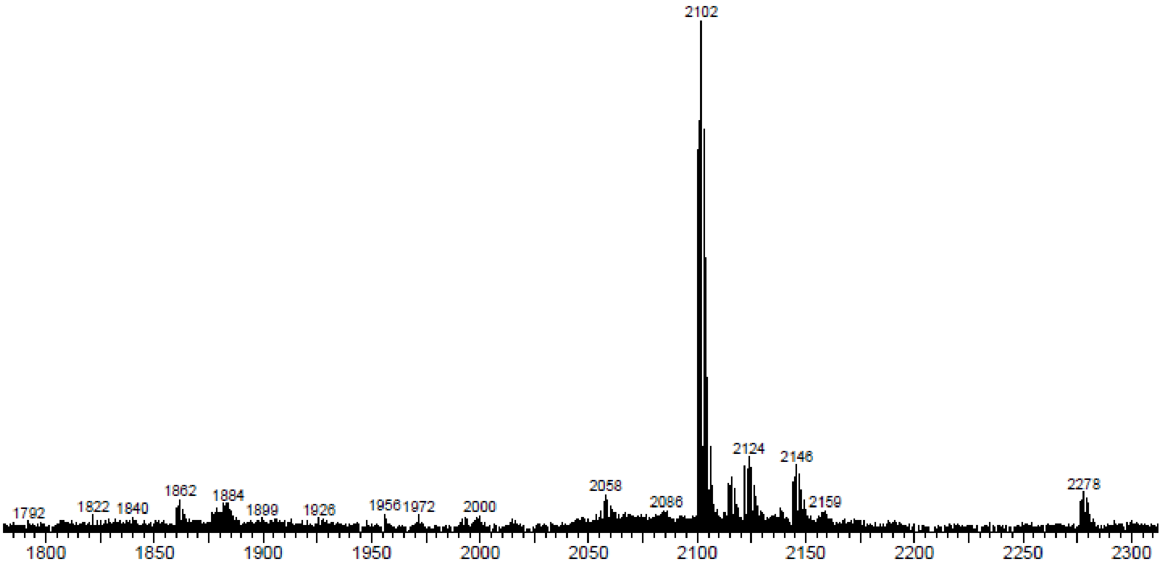

Figure S5.3 *m/z* (MALDI-ToF+) [**5**+Na]$^+$ = 2102, calc. $C_{92}H_{142}Cl_2N_4NaO_{44}^+$ = 2099.8. [**5**−H+2Na]$^+$ = 2124, and [**5**−2H+3Na]$^+$ = 2146.

5.4 Tris(mUp^{Cne}SmC^{Ac}-ODmtr) homostar, 7.

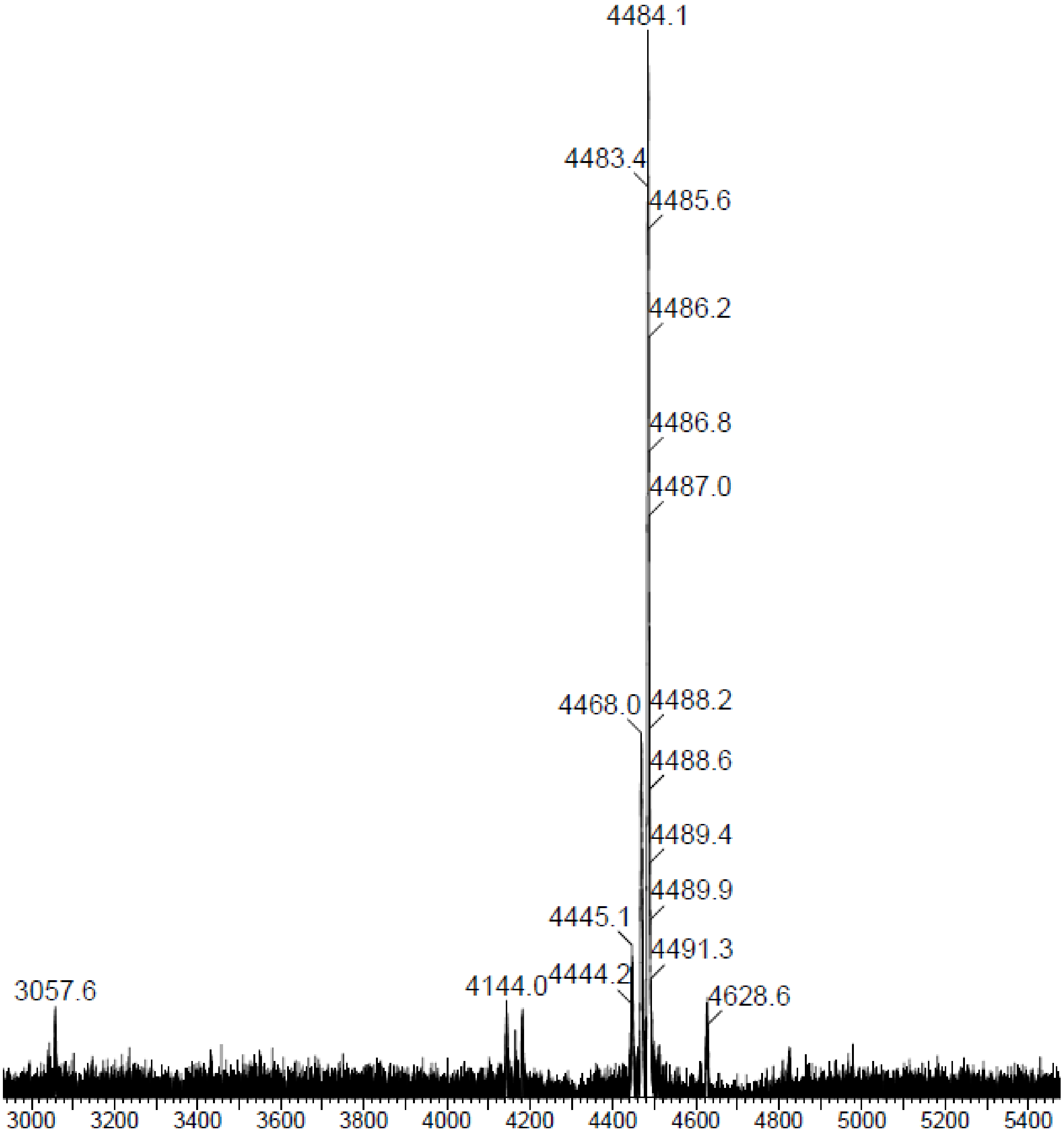

Figure S5.4 *m/z* (MALDI-ToF+) [**7**+Na+H_2O]$^+$ = 4484.1, calc. $C_{207}H_{269}N_{18}NaO_{79}P_3S_3^+$ = 4484.59; [**7**+Na]$^+$ = 4468.0, and [**7**+H]$^+$ = 4445.1. Peak at *m/z* = 4144.0 corresponds to detritylation during ionisation, calc. $C_{188}H_{250}N_{18}O_{76}P_3S_3^+$ = 4142.47, and peak at *m/z* = 3057.6 corresponds to a trace of benzyl ether hydrolysis, probably during ionisation, calc. $C_{141}H_{182}KN_{12}O_{53}P_2S_2^+$ = 3057.05.

5.5 Tris(mUp^{Cne}SmC^{Ac}-OH) homostar, 10.

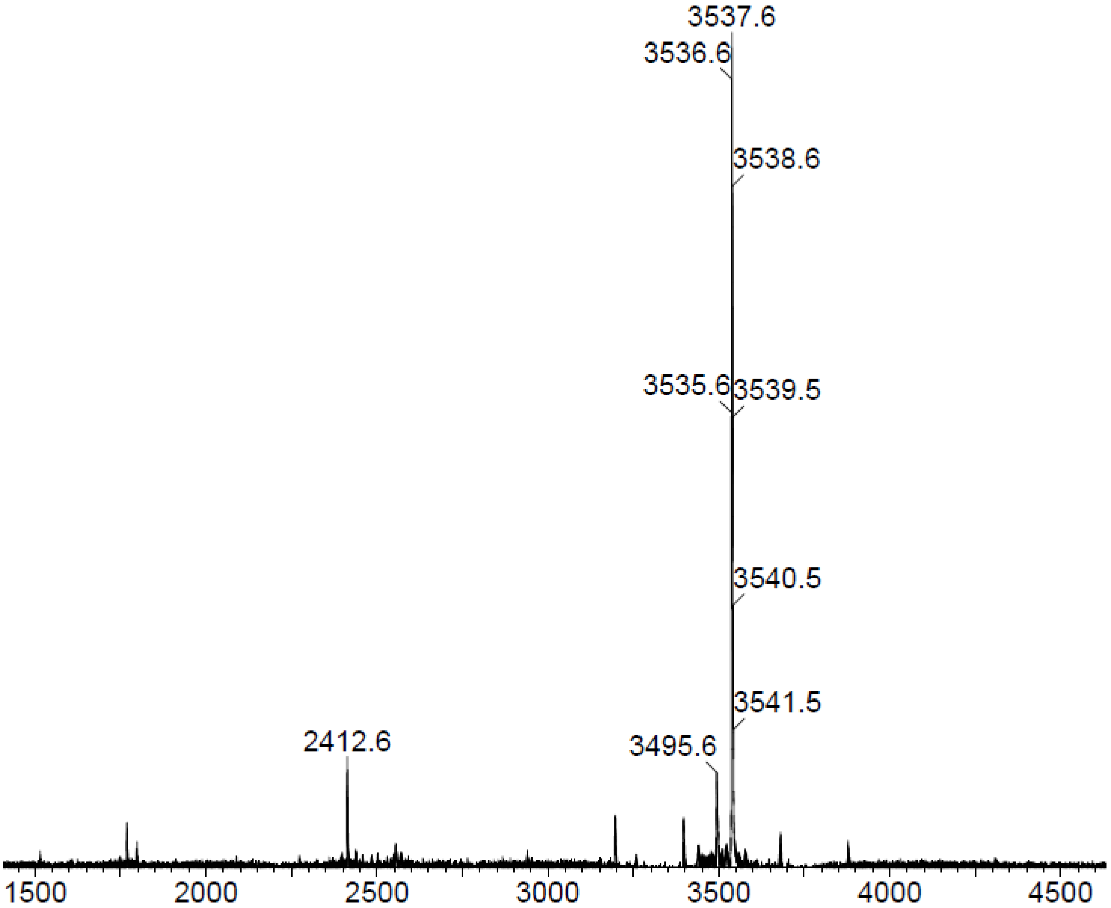

Figure S5.5 *m/z* (MALDI-ToF+) [**10**+H]$^+$ = 3538.3, calc. $C_{144}H_{214}N_{18}NaO_{72}P_3S_3^+$ = 3537.20; [**10**−Ac+2H]$^+$ = 3495.6, calc. $C_{142}H_{212}N_{18}O_{71}P_3S_3^+$ = 3495.19. Peak at *m/z* = 2412.6 corresponds to a trace of benzyl ether hydrolysis, probably during ionisation, calc. $C_{99}H_{147}N_{12}O_{49}P_2S_2^+$ = 2414.83. Notably there is no trace of incomplete chain extension as no peaks can be seen around [**10**−C^{Ac}*p*^{Cne}S+H]$^+$ calc. $C_{129}H_{195}N_{14}O_{65}P_2S_2^+$ = 3107.13.

5.6 LC-MS of tris(mUp^{Cne}SmC^{Ac}-OH) homostar, 10

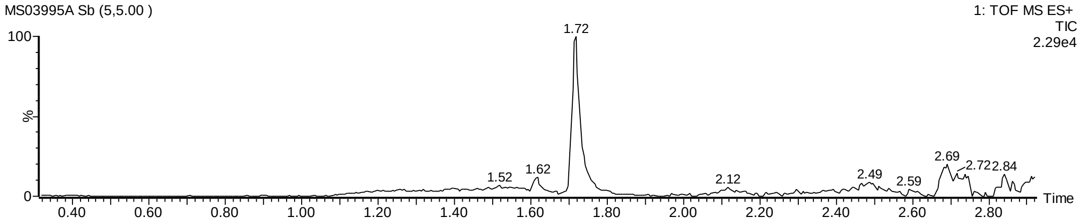

Figure S5.6.1 Ion chromatogram.

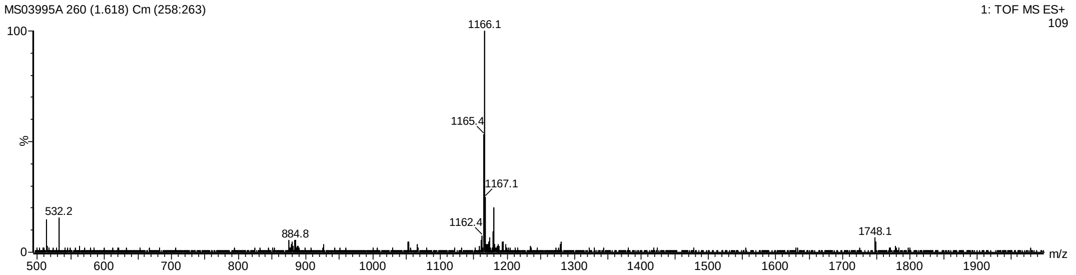

Figure S5.6.2 MS (ESI+) of peak at 1.62 min. Peak at *m/z* = 1166.1 corresponds to deacetylation of homostar **10**, [**10**−Ac+K+2H]$^{2+}$, calc = $C_{142}H_{212}KN_{18}O_{71}P_3S_3{}^{2+}$ = 1167.08.

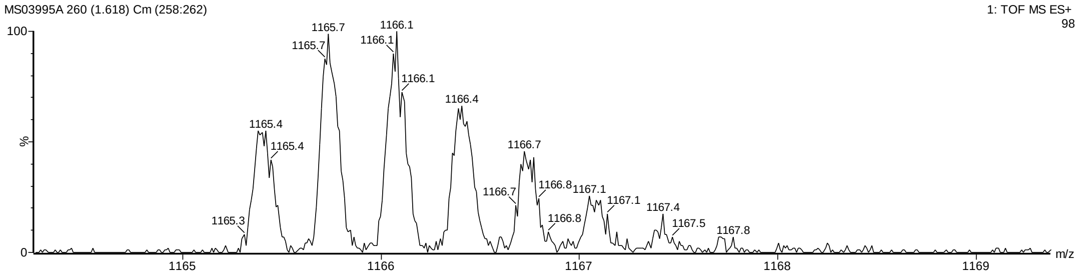

Figure S5.6.3 Expansion of impurity peak at 1.62 min, [**10**−Ac+K+H]$^{2+}$.

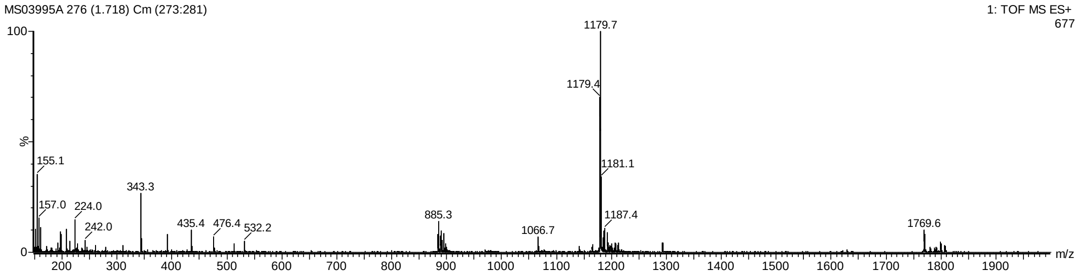

Figure S5.6.4 MS (ESI+) of main peak at 1.72 min, *m/z* = 1179.7 corresponds to tris(2-mer-OH) homostar [**10**+Na+H]$^{2+}$, calc. $C_{144}H_{214}N_{18}NaO_{72}P_3S_3{}^{2+}$ = 1780.09.

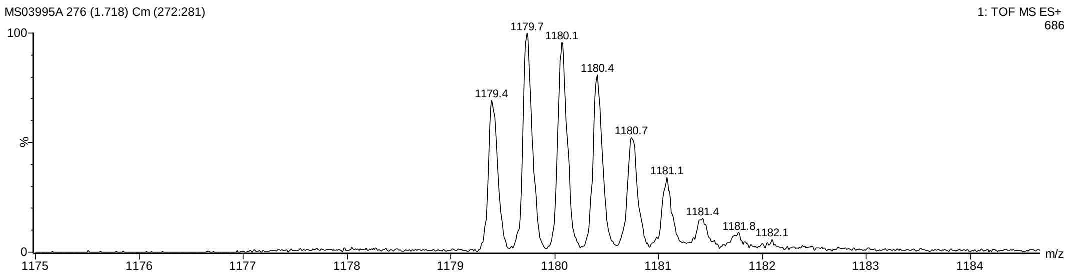

Figure S5.6.5 Expansion of molecular ion at 1.72 min, [**10**+Na+H]$^{2+}$.

5.7 Tris(mUp^{Cne}SmC^{Ac}p^{Cne}SmC^{Ac}-ODmtr) homostar, 13.

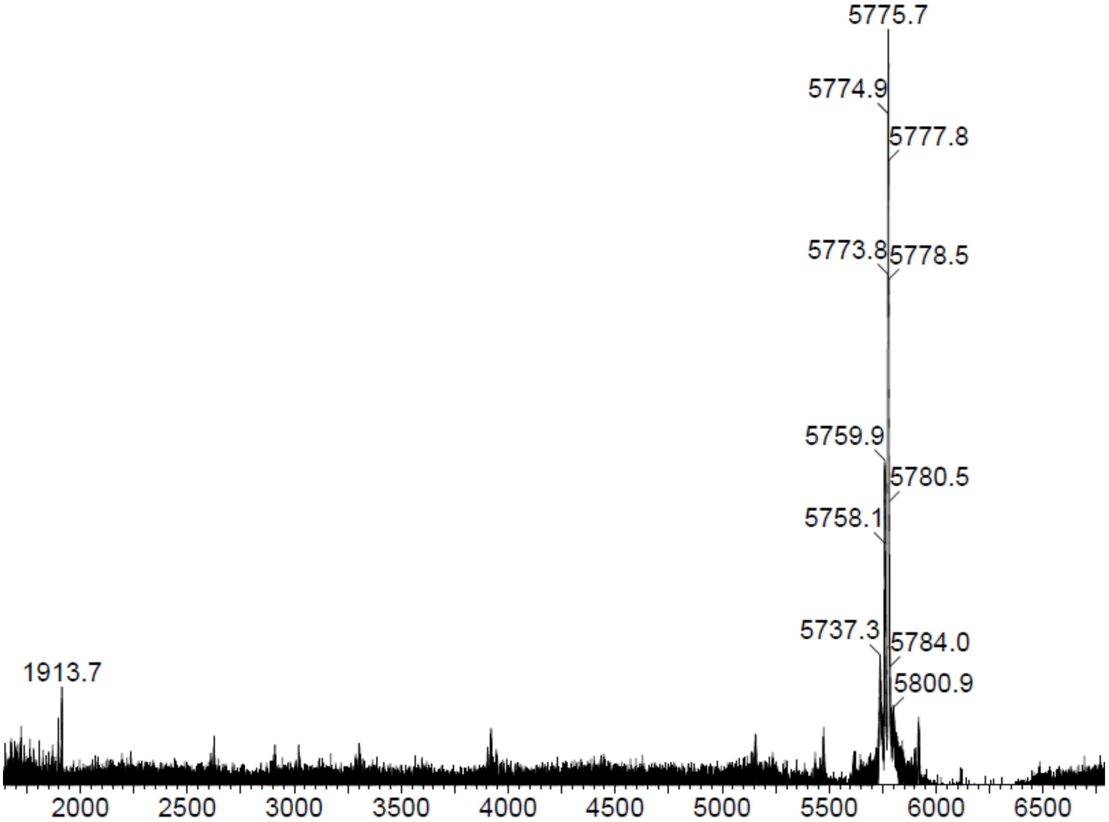

Figure S5.7 *m/z* (MALDI-ToF+) [**13**+Na+H_2O]$^+$ = 5775.7, calc. $C_{252}H_{326}N_{30}NaO_{100}P_6S_6^+$ = 5775.81; [**13**+Na]$^+$ = 5759.9. Peak at *m/z* = 3918.9 corresponds to a trace of benzyl ether hydrolysis, probably during ionisation, calc. $C_{171}H_{220}KN_{20}O_{67}P_4S_4^+$ = 3917.19, and *m/z* = 1913.7 is the corresponding trinucleotidyl Eg_8 species, calc. $C_{81}H_{106}N_{10}KO_{33}P_2S_2^+$ = 1911.55.

5.8 Tris(mUp^{Cne}SmC^{Ac}p^{Cne}SmC^{Ac}-OH) homostar, 14.

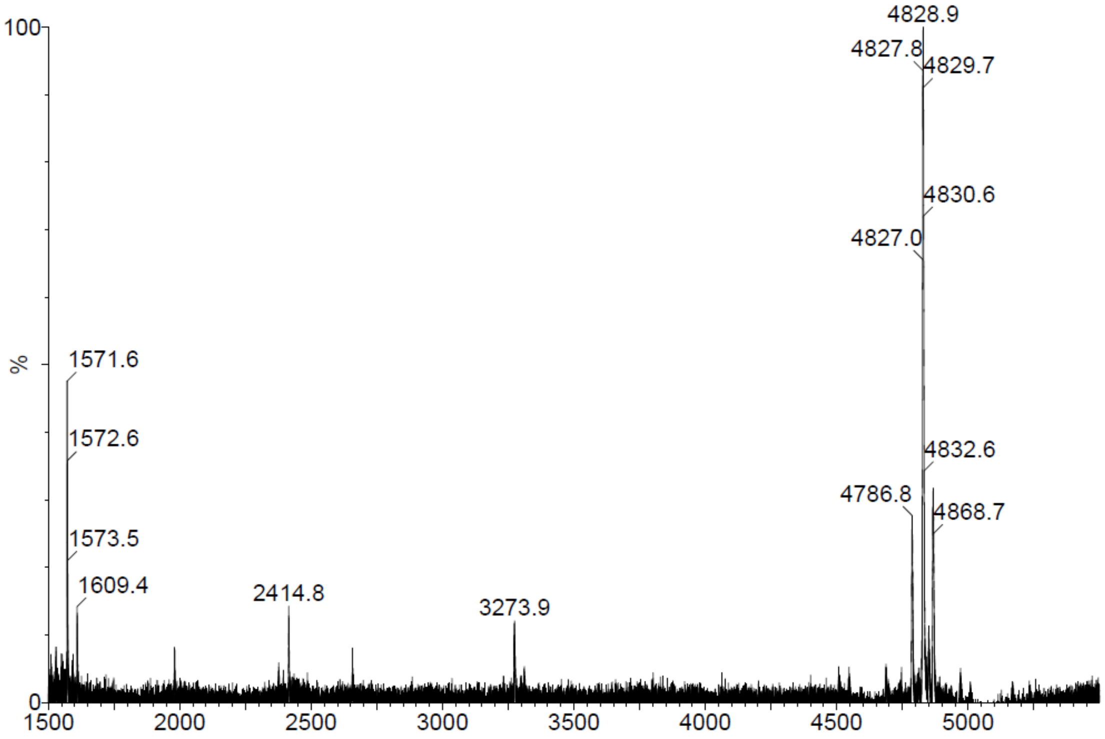

Figure S5.8 *m/z* (MALDI-ToF+) [**14**+H]$^+$ = 4828.9, calc. $C_{189}H_{271}N_{30}O_{93}P_6S_6^+$ = 4828.42; [**14**+Na+H_2O]$^+$ = 4868.7; [**14**+Na+H_3O]$^{2+}$ = 2414.8. [**14**−Ac+2H]$^+$ = 4886.8, calc. $C_{187}H_{269}N_{30}O_{92}P_6S_6^+$ = 4786.4. Peak at *m/z* = 3273.9 corresponds to a trace of benzyl ether hydrolysis, probably during ionisation, calc. $C_{129}H_{185}N_{20}O_{63}P_4S_4^+$ = 3274.98, and *m/z* = 1571.6 is the corresponding trinucleotidyl Eg_8 species, calc. $C_{60}H_{89}N_{10}O_{31}P_2S_2^+$ = 1571.46. NB: There are no peaks around *m/z* = 5216 which would indicate branching on cytosine unprotected 6-NH_2, nor again around *m/z* = 4397 which would indicate incomplete coupling.

5.9 Tris(mUp^{Cne}SmC^{Ac}p^{Cne}SmC^{Ac}p^{Cne}SmA^{Bz}-ODmtr) homostar, 15.

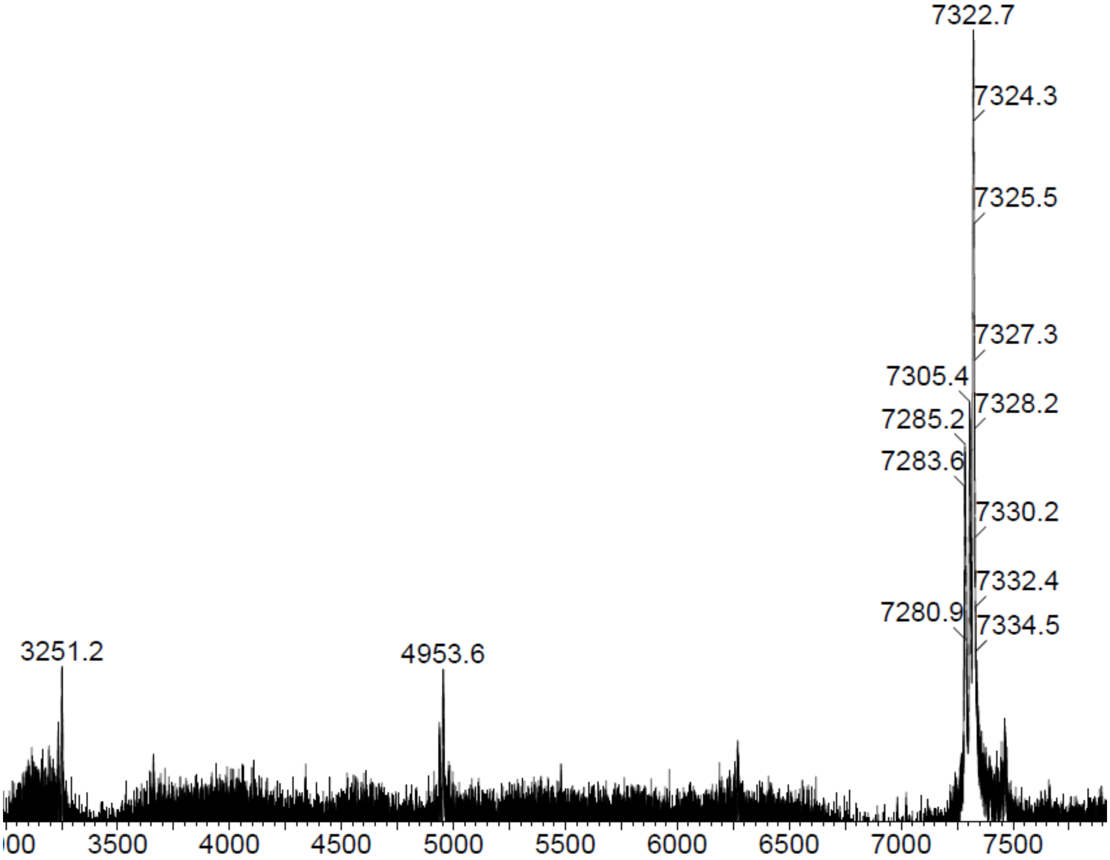

Figure S5.9 *m/z* (MALDI-ToF+) [**15**+K]$^+$ = 7322.7, calc. $C_{315}H_{387}N_{48}KO_{117}P_9S_9^+$ = 7323.07; [**15**+Na]$^+$ = 7305.4; [**15**+H]$^+$ = 7285.2. Peak at *m/z* = 4953.6 corresponds to a trace of benzyl ether hydrolysis, probably during ionisation, calc. $C_{213}H_{264}N_{32}NaO_{80}P_6S_6^+$ = 4953.43.

5.10 Tris(mUp^{Cne}SmC^{Ac}p^{Cne}SmC^{Ac}p^{Cne}SmA^{Bz}-OH) homostar, 16.

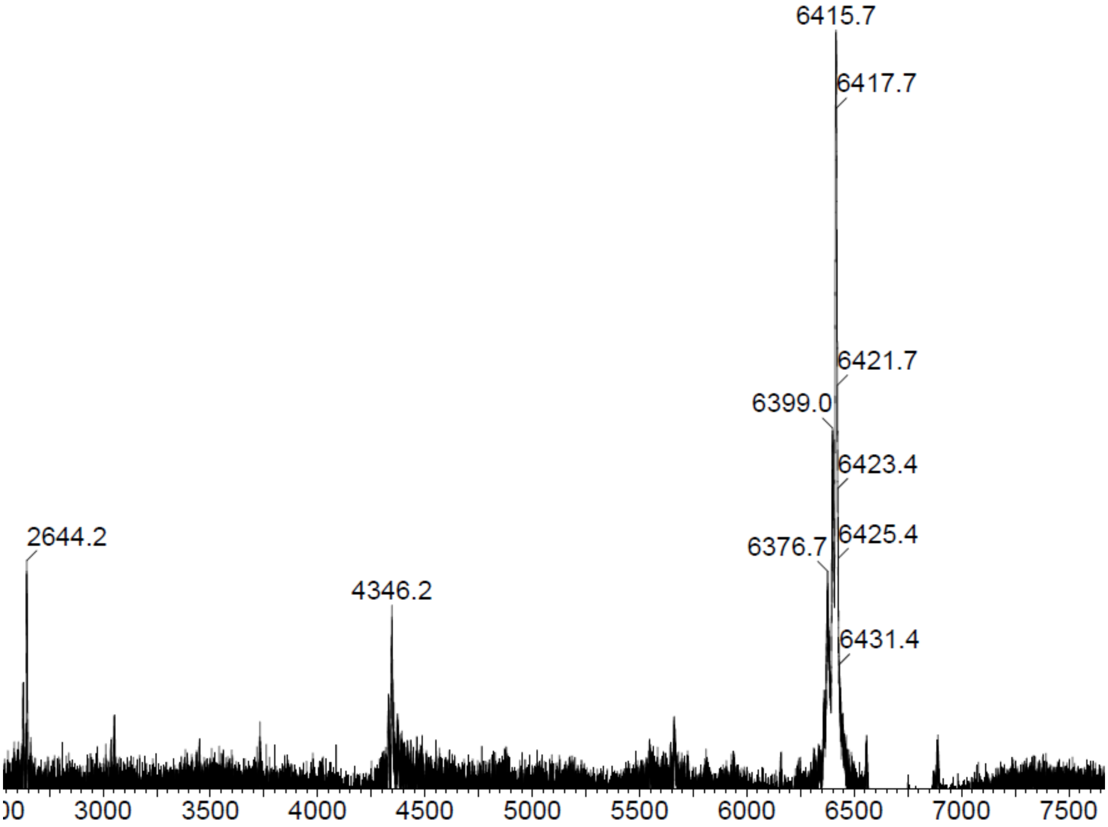

Figure S5.10 *m/z* (MALDI-ToF+) [**16**+K]$^+$ = 6416.7, calc. $C_{252}H_{333}KN_{48}O_{111}P_9S_9^+$ = 6414.67. Peak at *m/z* = 4346.2 corresponds to a trace of benzyl ether hydrolysis, probably during ionisation, calc. $C_{171}H_{225}N_{32}KO_{75}P_6S_6^+$ = 4346.13. NB: There around no peaks *m/z* = 5899 which would indicate incomplete coupling.

5.11 Tris(mUp^{Cne}SmC^{Ac}p^{Cne}SmC^{Ac}p^{Cne}SmA^{Bz}p^{Cne}SmU-ODmtr) homostar, 17.

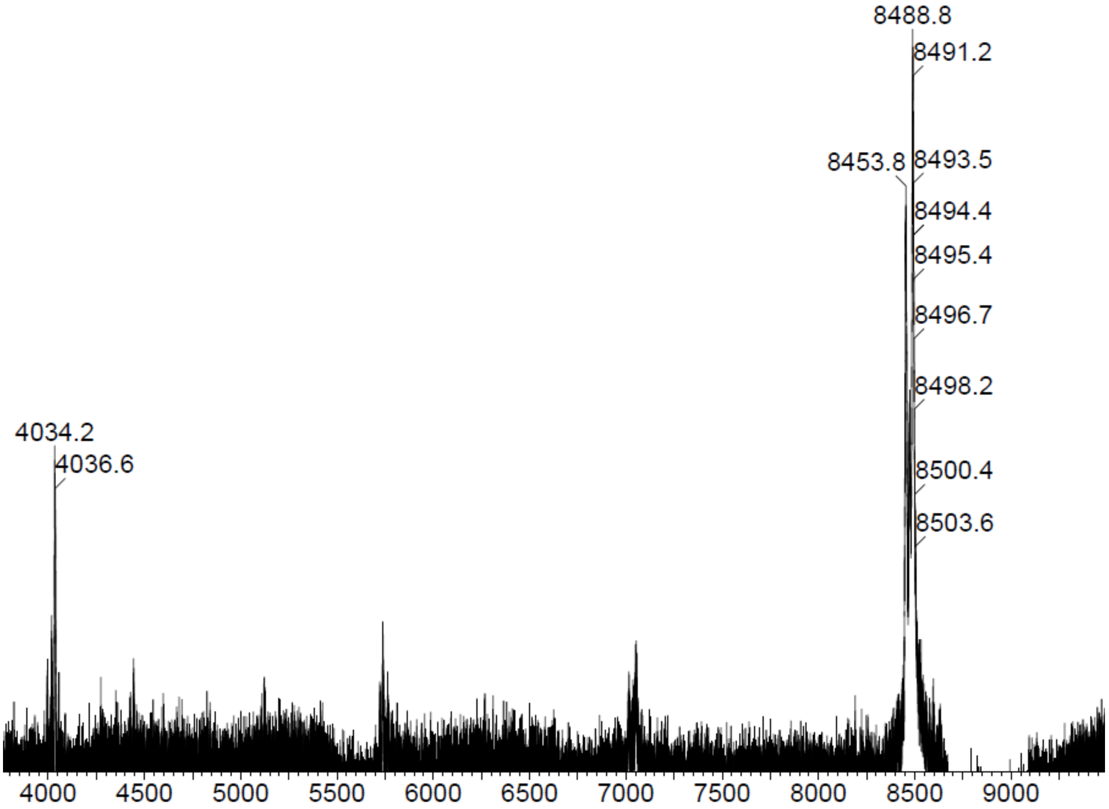

Figure S5.11 *m/z* (MALDI-ToF+) [**17**+K]$^{+}$ = 8488.8, calc. $C_{354}H_{435}KN_{57}O_{138}P_{12}S_{12}^{+}$ = 8489.20; [**17**+H]$^{+}$ = 8453.8.

5.12 Tris(mUp^{Cne}SmC^{Ac}p^{Cne}SmC^{Ac}p^{Cne}SmA^{Bz}p^{Cne}SmU-OH) homostar, 18.

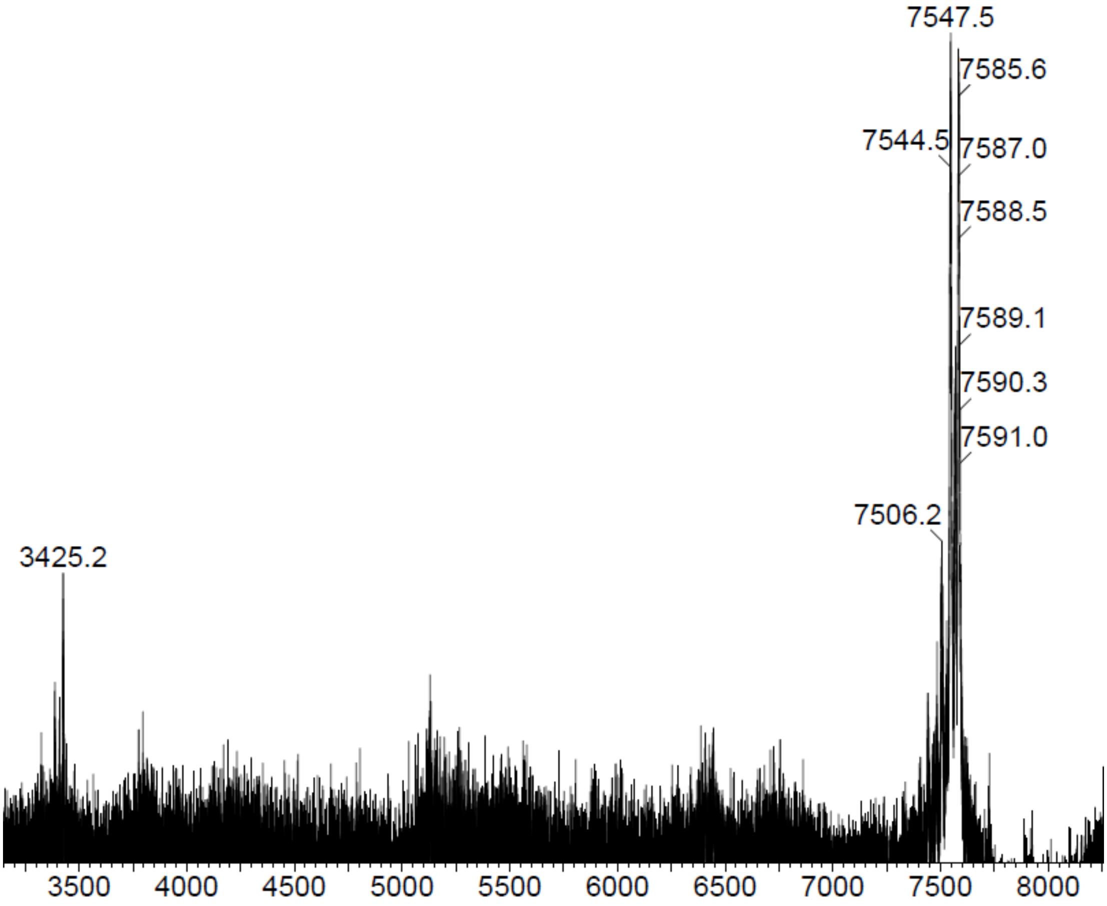

Figure S5.12 *m/z* (MALDI-ToF+) [**18**+H]$^+$ = 7547.5, calc. $C_{291}H_{382}N_{57}O_{132}P_{12}S_{12}^+$ = 7544.85; [**18**+K]$^+$ = 7585.6. NB: There no peaks around *m/z* = 7193 which would indicate incomplete coupling.

5.13 LC-MS of crude HO-mUpSmCpSmCpSmApSmUp-OH, 19.

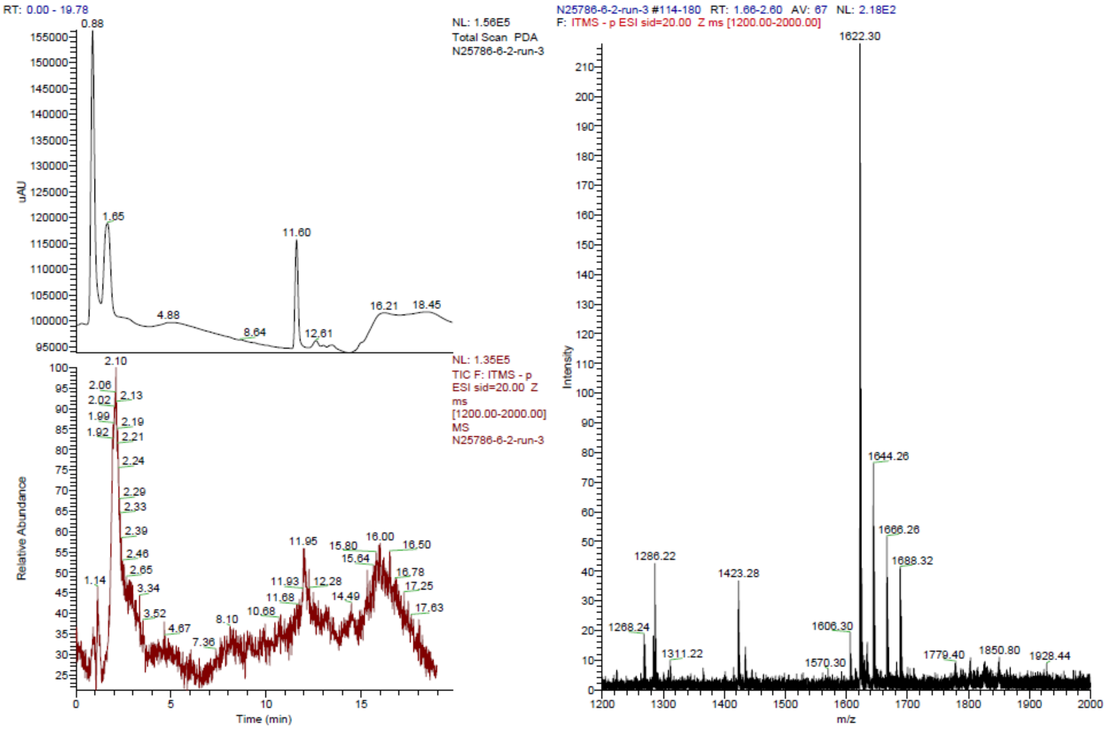

Figure S5.13 Top left) HPLC trace; bottom left) ion chromatogram; right) wide range total MS (ESI+) of peak from 1.66 to 2.60 min, *m/z* [**19**+H]$^+$ = 1622.30, calc. $C_{51}H_{70}N_{15}O_{30}P_4S_4^+$ = 1624.22.

5.14 Tris(mUp^{Cne}SmC^{Ac}p^{Cne}SmC^{Ac}p^{Cne}SmA^{Bz}p^{Cne}SmUp^{Cne}SmU-ODmtr) homostar, 20.

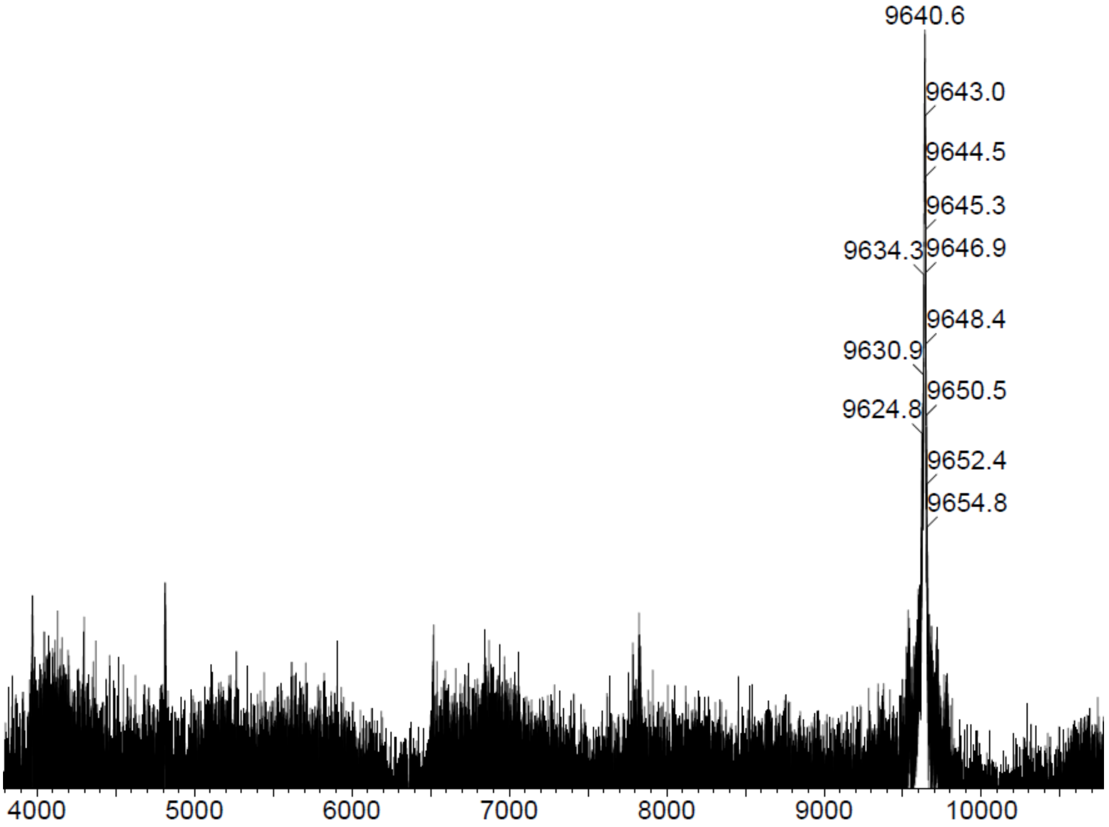

Figure S5.14 *m/z* (MALDI-ToF+) [**20**+Na]$^+$ = 9644, calc. $C_{393}H_{483}N_{66}NaO_{159}P_{15}S_{15}^+$ = 9641.4.

5.15 Tris(mUp^{Cne}SmC^{Ac}p^{Cne}SmC^{Ac}p^{Cne}SmA^{Bz}p^{Cne}SmUp^{Cne}SmU-OH) homostar, 21.

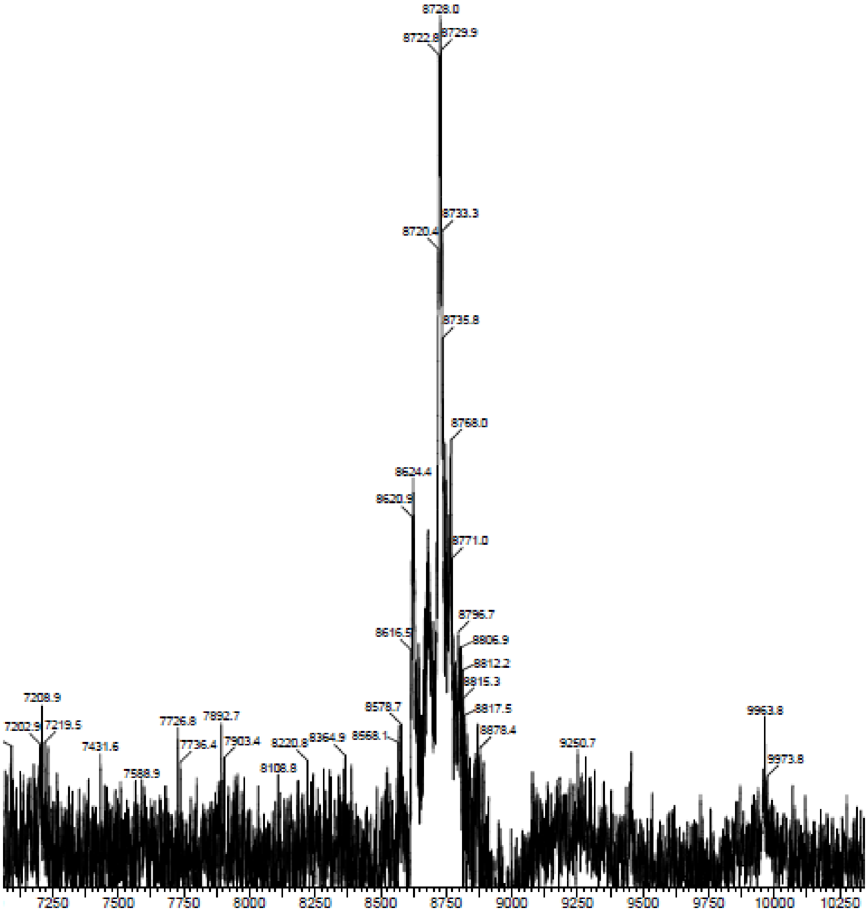

Figure S5.15 *m/z* (MALDI-ToF+) [**21**+Na]$^{+}$ = 8728, calc. $C_{330}H_{429}N_{66}NaO_{153}P_{15}S_{15}^{+}$ = 8734.0.

5.16 Tris(mUp^{Cne}SmC^{Ac}p^{Cne}SmC^{Ac}p^{Cne}SmA^{Bz}p^{Cne}SmUp^{Cne}SmUp^{Cne}SmC^{Ac}-ODmtr) homostar, 22.

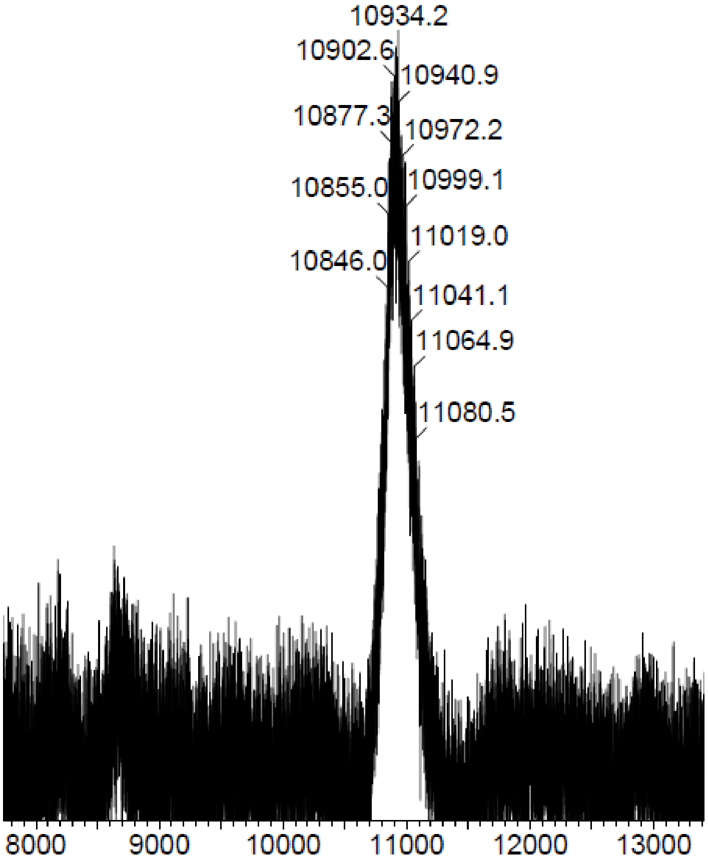

Figure S5.16 *m/z* (MALDI-ToF+) [**22**+Na]$^+$ = 10934, calc. $C_{438}H_{540}N_{78}NaO_{180}P_{18}S_{18}^+$ = 10932.6.

5.17 Tris(mUp^{Cne}SmC^{Ac}p^{Cne}SmC^{Ac}p^{Cne}SmA^{Bz}p^{Cne}SmUp^{Cne}SmUp^{Cne}SmC^{Ac}-OH) homostar, 23.

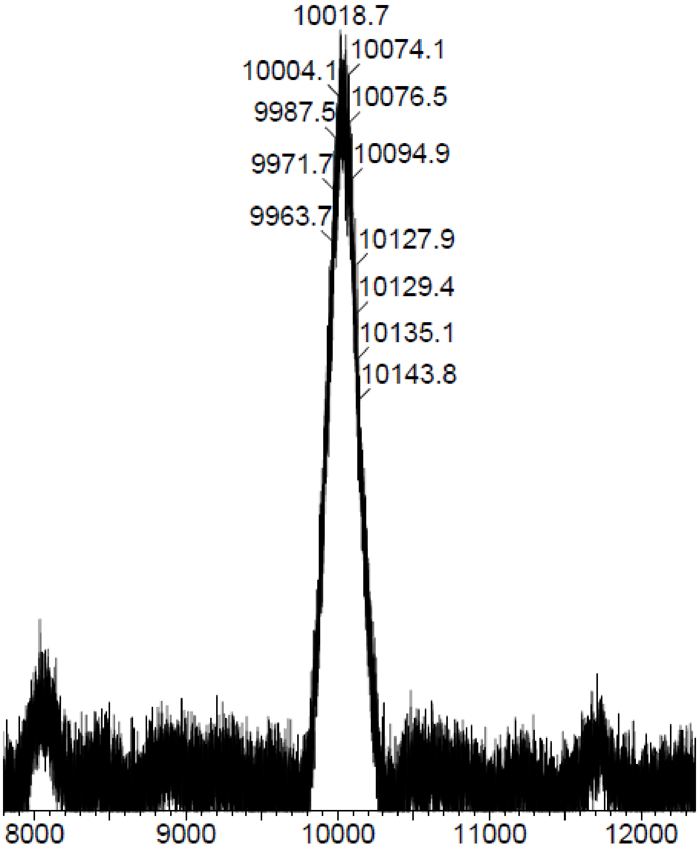

Figure S5.17 *m/z* (MALDI-ToF+) [**22**+Na]$^+$ = 10019, calc. $C_{375}H_{486}N_{78}NaO_{174}P_{18}S_{18}^+$ = 10024.2

5.18 Tris(mUp^{Cne}SmC^{Ac}p^{Cne}SmC^{Ac}p^{Cne}SmA^{Bz}p^{Cne}SmUp^{Cne}SmUp^{Cne}SmC^{Ac}p^{Cne}SmG^{iBu}-ODmtr) homostar, 24.

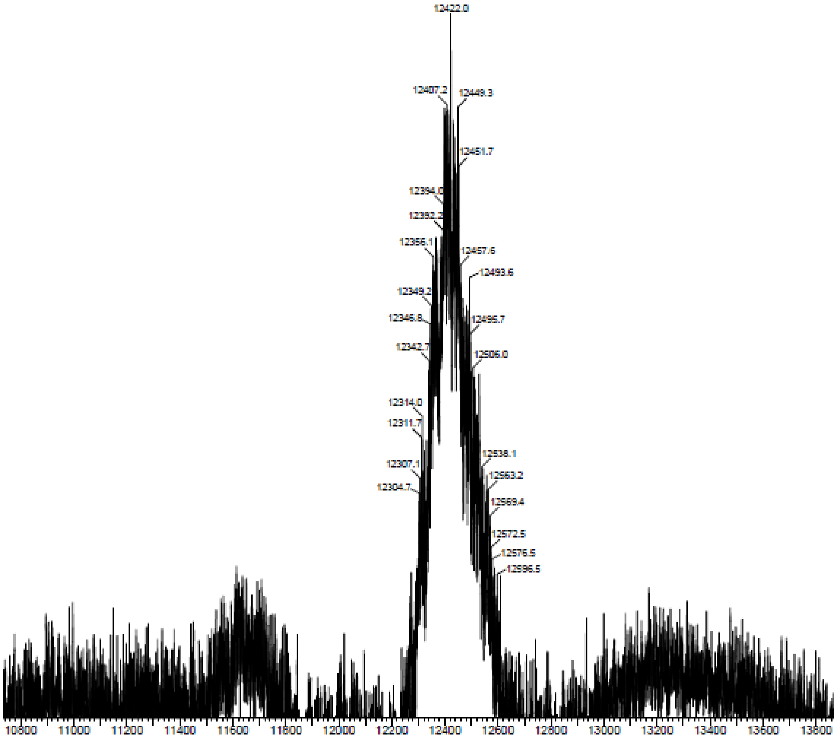

Figure S5.18 *m/z* (MALDI-ToF+) [**24**+Na]$^+$ = 12422, calc. $C_{492}H_{609}N_{96}NaO_{201}P_{21}S_{21}{}^+$ = 12426.9.

5.19 Tris(mUp^{Cne}SmC^{Ac}p^{Cne}SmC^{Ac}p^{Cne}SmA^{Bz}p^{Cne}SmUp^{Cne}SmUp^{Cne}SmC^{Ac}p^{Cne}SmG^{iBu}-OH) homostar, 25.

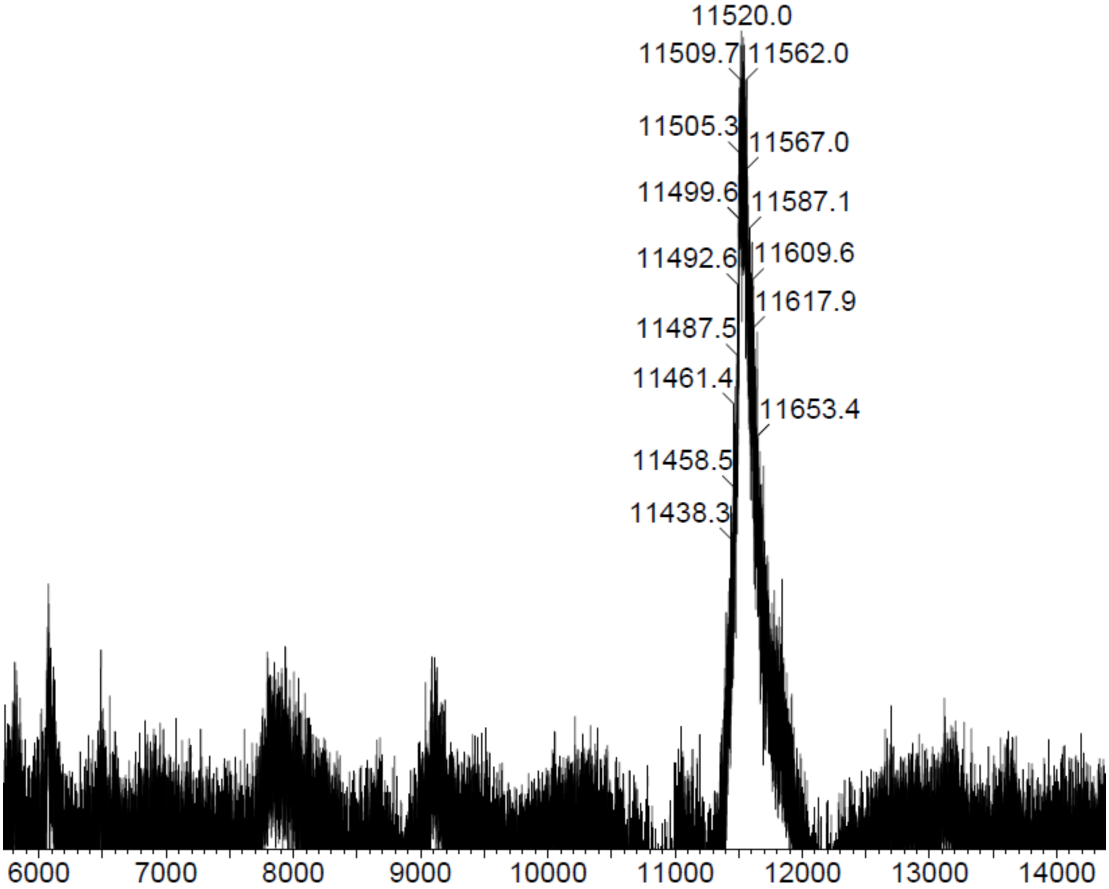

Figure S5.19 *m/z* (MALDI-ToF+) [**25**+Na]$^{+}$ = 11520.0, calc. $C_{429}H_{555}N_{96}NaO_{195}P_{21}S_{21}^{+}$ = 11520.52.

5.20 Tris(mUp^{Cne}SmC^{Ac}p^{Cne}SmC^{Ac}p^{Cne}SmA^{Bz}p^{Cne}SmUp^{Cne}SmUp^{Cne}SmC^{Ac}p^{Cne}SmG^{iBu}p^{Cne}SmG^{iBu}-ODmtr) homostar, 26.

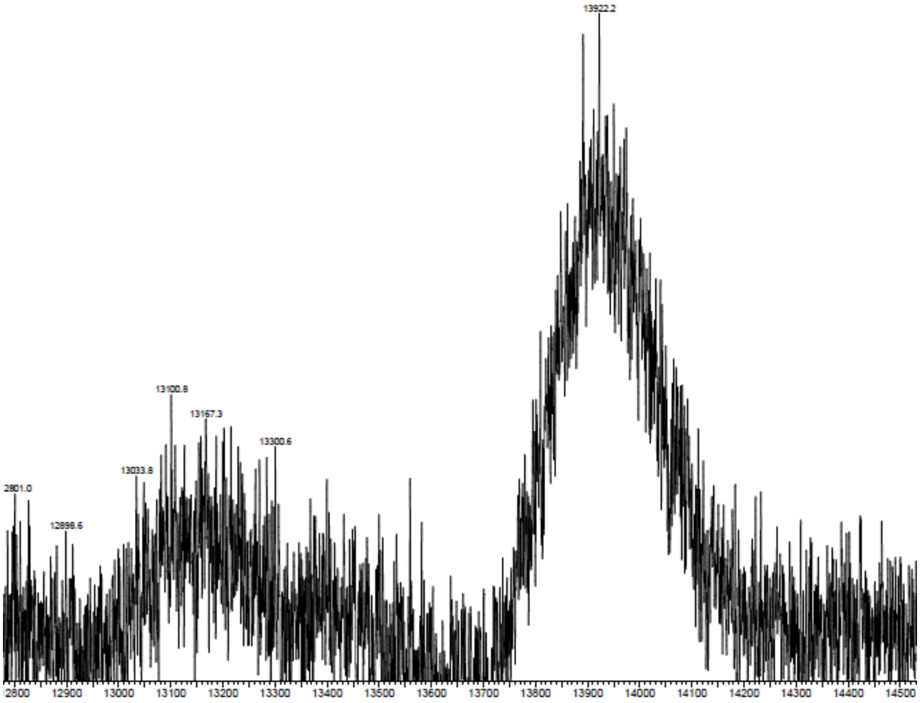

Figure S5.20 *m/z* (MALDI-ToF+) [**26**+Na]$^+$ = 13922, calc. $C_{546}H_{678}N_{114}NaO_{222}P_{24}S_{24}^+$ = 13921.2. Peak at *m/z* = 13101 corresponds to incomplete coupling on one arm of the homostar, $C_{507}H_{637}N_{108}NaO_{213}P_{23}S_{23}^+$ = 13121.

5.21 Tris(mUp^{Cne}SmC^{Ac}p^{Cne}SmC^{Ac}p^{Cne}SmA^{Bz}p^{Cne}SmUp^{Cne}SmUp^{Cne}SmC^{Ac}p^{Cne}SmG^{iBu}p^{Cne}SmG^{iBu}-OH) homostar, 27.

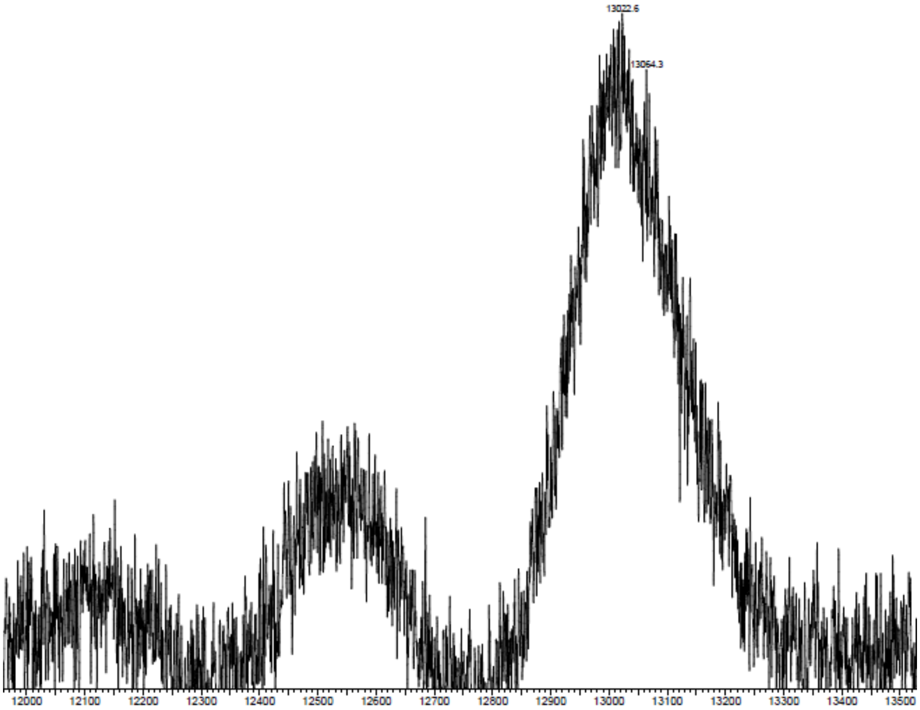

Figure S5.21 *m/z* (MALDI-ToF+) [**27**+Na]$^{+}$ = 13023, calc. $C_{483}H_{624}N_{114}NaO_{216}P_{24}S_{24}^{+}$ = 13014.8. Peak at *m/z* = 12505 corresponds to incomplete coupling on one arm of the homostar, $C_{465}H_{601}N_{108}NaO_{209}P_{23}S_{23}^{+}$ = 12516.7.

5.22 LC-MS of purified HO-mUpSmCpSmCpSmApSmUpSmUpSmCpSmGpSmG-OH, 28.

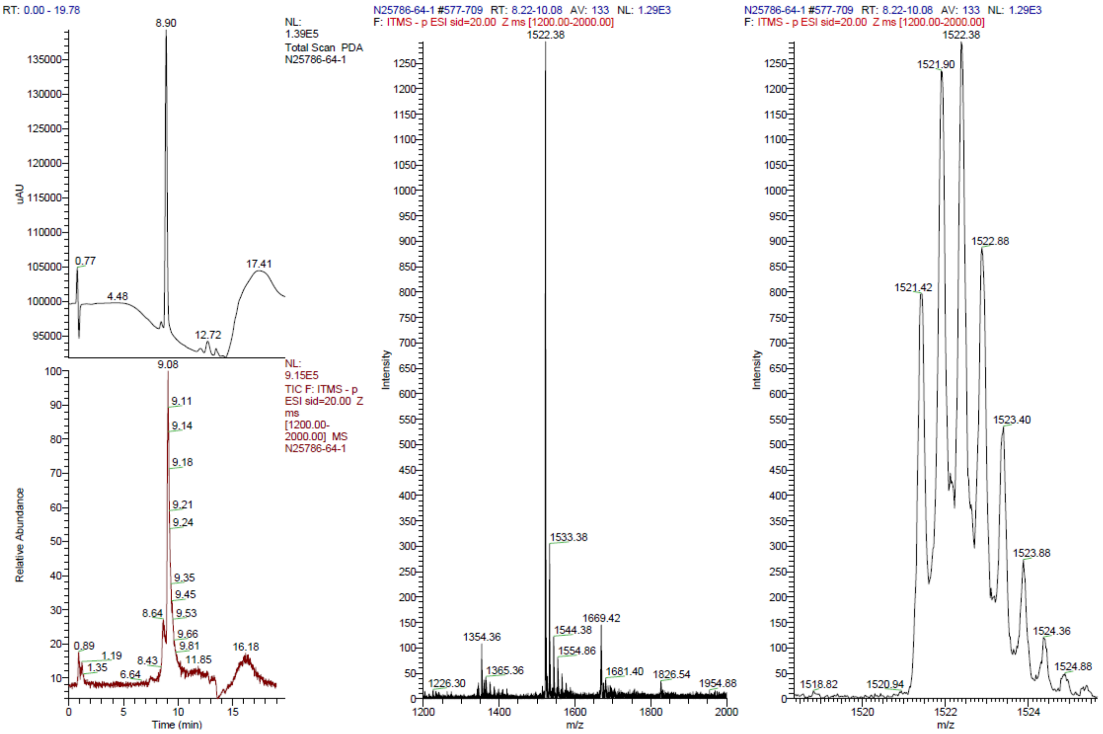

Figure S5.22 Top left) HPLC trace; bottom left) ion chromatogram; middle) wide range total MS (ESI+) of peak from 8.22 to 10.08 min – *m/z* = 1354.36 corresponds to 8-mer impurity [**29**+2H]$^{2+}$, calc. $C_{82}H_{112}N_{25}O_{49}P_7S_7^{2+}$ = 1335.66, but the peak centred on 1669 is broad and not assignable to a single charge state, or single species; right) expansion of molecular ion, [**28**+2H]$^{2+}$, calc. $C_{93}H_{126}N_{30}O_{55}P_8S_8^{2+}$ = 1523.68.

6. HPLC of Homostars

HPLC of oligonucleotidyl homostars

HPLC analyses were performed using an *Agilent* 1100 Series model, equipped with a UV detector, plus an ELSD detector (*Varian, UK*, 385-LC). A gradient of MeOH and water, buffered with 100 mM ammonium acetate, was eluted through an ACE C18 RP column (*Hichrom Ltd, UK*). The injection volume was 30 µL, the pump flow-rate was 1 mLl.min^{-1}, the column temperature was fixed at 30 °C, and the UV detector wavelength was set at 260 nm. The ELSD evaporation temperature was 40 °C, nebulization temperature was 55 °C, and the nitrogen gas flow rate at 1.5 SLM (standard liter per minute).

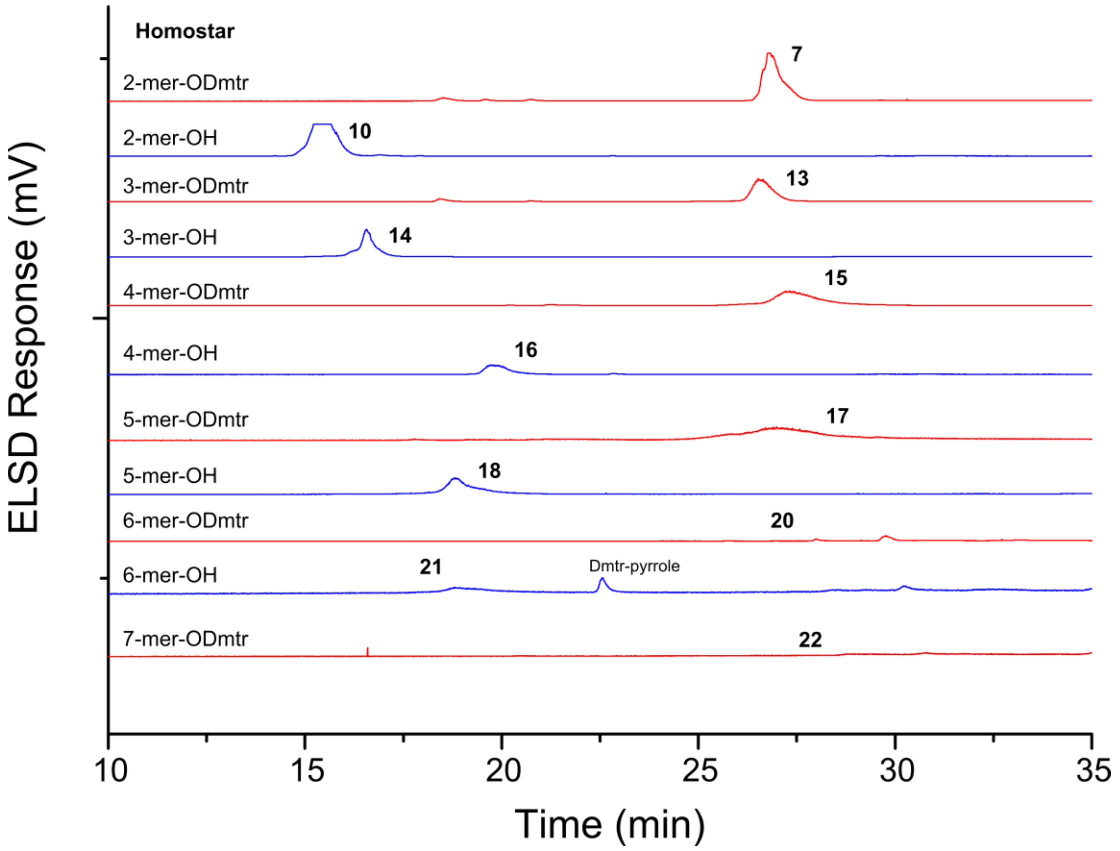

Figure S6 HPLC-ELSD chromatograms after OSN of oligonucleotidyl homostar chain extension (blue) and detritylation (red) retentates.

The HPLC chromarograms became progressively broader with increasing oligo length, until around 7-mer it became impossible to monitor the reaction directly. In future we will employ degradative analysis.
